# Syntheses and Investigations of Conformationally Restricted, Linker-Free α-Amino Acid–BODIPYs via Boron Functionalization

**DOI:** 10.1021/acs.joc.1c02328

**Published:** 2021-11-22

**Authors:** Maodie Wang, Guanyu Zhang, Petia Bobadova-Parvanova, Kevin M. Smith, M. Graça H. Vicente

**Affiliations:** † Department of Chemistry, Louisiana State University, Baton Rouge, Louisiana 70803, United States; ‡ Department of Chemistry and Fermentation Sciences, Appalachian State University, Boone, North Carolina 28607, United States

## Abstract

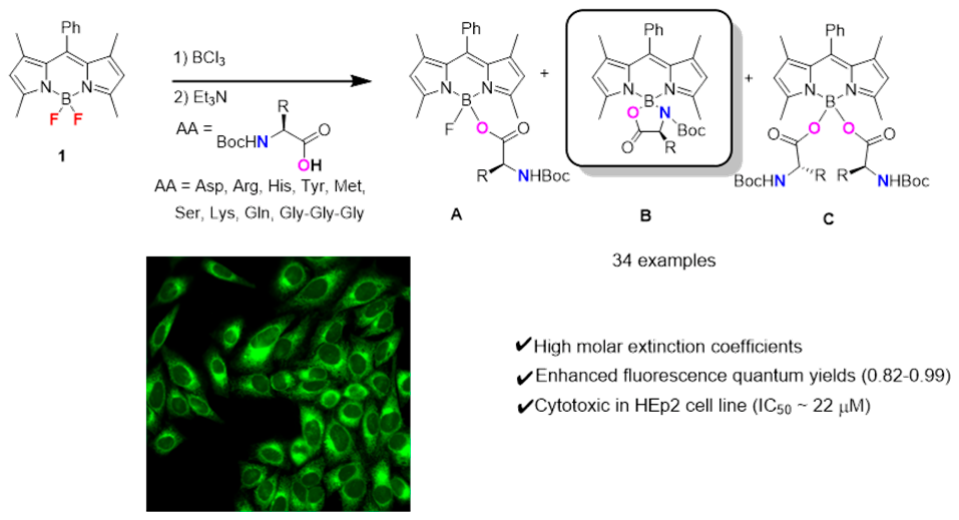

A series of α-amino acid–BODIPY derivatives were synthesized using commercially available *N*-Boc-l-amino acids, via boron functionalization under mild conditions. The mono-linear, mono-spiro, and di-amino acid–BODIPY derivatives were obtained using an excess of basic (histidine, lysine, and arginine), acidic (aspartic acid), polar (tyrosine, serine), and nonpolar (methionine) amino acid residues, in yields that ranged from 37 to 66%. The conformationally restricted mono-spiro- and di-amino acid–BODIPYs display strong absorptions in the visible spectral region with high molar extinction coefficients and significantly enhanced fluorescence quantum yields compared with the parent BF_2_–BODIPY. Cellular uptake and cytotoxicity studies using the human HEp2 cell line show that both the presence of an *N*,*O*-bidentate spiro-ring and basic amino acids (His and Arg) increase cytotoxicity and enhance cellular uptake. Among the series of BODIPYs tested, the spiro-Arg- and spiro-His-BODIPYs were found to be the most cytotoxic (IC_50_ ∼ 22 μM), while the spiro-His-BODIPY was the most efficiently internalized, localizing preferentially in the cell lysosomes, ER, and mitochondria.

## Introduction

Boron dipyrrin or boron dipyrromethene (BODIPY) dyes^1−6^ are a class of boron-coordinated organic dyes that display a multitude of highly desirable properties for various applications, including good solubility in various solvents, large molar extinction coefficients, high fluorescence quantum yields, relatively sharp absorption and emission bands, low cytotoxicity, and structural tunability. With these extraordinary properties, BODIPY dyes have been widely applied in, for example, live-cell bioimaging,^7,8^ photodynamic therapy,^9,10^ fluorescent sensing,^11^ dye-sensitized solar cells,^12^ and viscosity detection.^13^

Several methodologies for the post-functionalization of BODIPYs have been developed over the past 2 decades.^12,14^ The absorption and emission properties of BODIPY dyes can be fine-tuned from ca. 400^15,16^ to 750 nm through functionalization reactions at the dipyrrin core.^14^ In contrast, modifications at the boron center generally have little or no effect on the absorption and emission wavelengths, although the 3D structures of the boron-functionalized BODIPY molecules often change significantly upon the introduction of various groups at the boron atom. As a result, the fluorescence quantum yields, laser efficiencies,^17^ redox behavior, aqueous solubility, aggregation behavior, and chemical stability^18−20^ of BODIPYs can be conveniently fine-tuned for specific applications. Nucleophilic substitution of one or both fluorides at the boron position provides the possibility for application in positron emission tomography–optical dual imaging by replacing the nonradiative fluorine atom(s) by ^18^F.^21^ Other investigations of C-, O-, and N-BODIPYs^22−25^ have led to enhanced materials for applications in energy-transfer cassettes, fluorescence imaging, and chiral recognition, among others. However, only a few reports have explored bidentate B-spiro-BODIPYs.^26−28^

Amino acid–fluorophore conjugates are important building blocks for the construction of bioactive fluorescent peptides and proteins. The synthesis of BODIPY–amino acid conjugates has been reported through C–H activation^29,30^ and nucleophilic aromatic substitution reactions.^31^ We have previously reported the synthesis of *N*,*O*-bidentate spiro-Gly-BODIPY derivatives by direct functionalization at the boron atom^32^ and extended this methodology to the preparation of near-IR aza-BODIPY-Gln derivatives with potential application in photodynamic therapy.^33^

In the past few decades, the biological activities of boron-containing compounds have been investigated for their antifungal, antibacterial, antiviral, anti-inflammatory, and antiprotozoal activities.^34−37^ Notably, tavaborole (Kerydin), bortezomib (Velcade), and crisaborole (Eucrisa) have been studied for the treatment of onychomycosis, multiple myeloma, and atopic dermatitis, respectively.^38^ Recently, in a study of NLRP3 inflammasome inhibitors, Freeman et al. reported that conformationally restricted analogues of a diarylboronic acid motif and an oxazaborine ring possessed enhanced anti-inflammatory activity.^39,40^ Motivated by these studies, we set out to synthesize and investigate a series of conformationally restricted mono-spiro- and di-amino acid–BODIPY derivatives, bearing basic (histidine, lysine, and arginine), acidic (aspartic acid), polar (tyrosine, serine, and glutamine), and nonpolar (methionine) amino acid residues for potential therapeutic and/or imaging applications. In addition, a tripeptide (Gly)_3_–BODIPY derivative was also prepared, showing that this methodology can be extended to the synthesis of linker-free BODIPY–peptide derivatives.

## Results and Discussion

### Synthesis

**Scheme 1:**
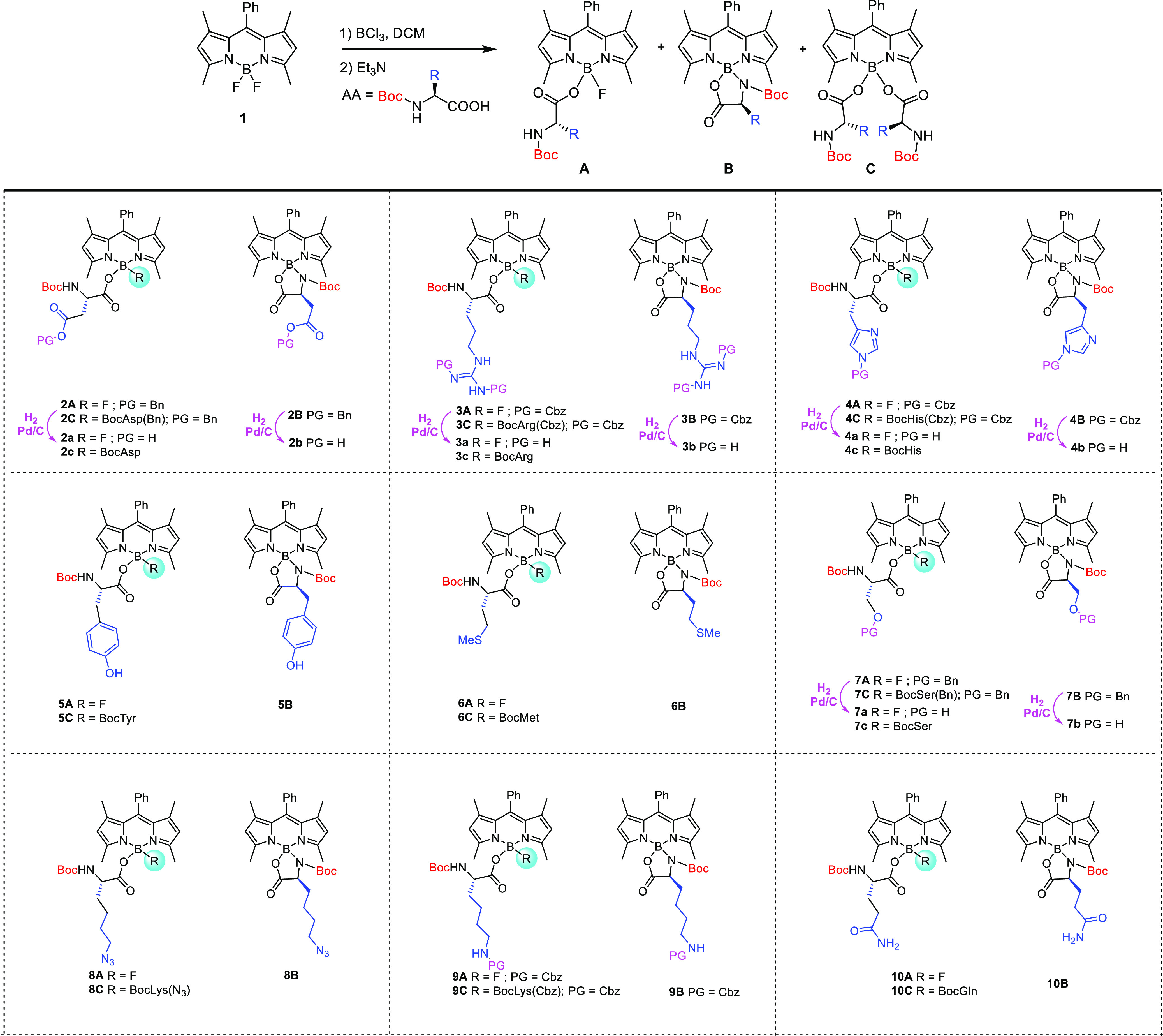
Synthesis of α-Amino Acid–BODIPY Derivatives

BODIPY **1** was synthesized through a one-pot three-step method using 2,4-dimethylpyrrole and benzaldehyde as the starting materials following a reported procedure.^41^ The reaction of BODIPY **1** with commercially available *N*-Boc l-amino acids, in the presence of boron trichloride, has wide substrate scope, as shown in Scheme 1. α-Amino acids bearing polar, nonpolar, cationic, and anionic side chains were used to produce the mono-(**A**), *N*,*O*-bidentate spiro-(**B**), and di-(**C**) α-amino acid BODIPY derivatives, in combined yields ranging from 37.3 to 65.9%. While BODIPYs **2** (Asp), **6** (Met), and **9** (Lys) were obtained in moderate yields (>60%), BODIPYs **4** (His) and **5** (Tyr) were isolated in lower yields (∼40%), possibly due to the poor solubility of the amino acids in dichloromethane, as well as potential attack from the side chain on the boron center. A two-step one-pot reaction, previously reported using Gly^32^ and Gln,^33^ using two equivalents of boron trichloride and four equivalents of l-amino acid in dry dichloromethane at room temperature, was employed to favor the formation of conformationally restricted products **B** and **C**. The *tert*-butyloxycarbonyl (Boc) electron-withdrawing group on the N-terminus of the amino acid is necessary for decreasing the charge on boron, therefore leading to stable N(sp^3^) conjugates. Some amino acids bearing nucleophilic side chains also required the use of side chain protecting group(s) to avoid byproduct formation via a side chain attack on the boron atom. However, the widely used 9-fluorenylmethyloxycarbonyl (Fmoc) and trifluoroacetyl protecting groups require harsh conditions for deprotection,^42^ which could also remove the *N*-Boc group and/or cause BODIPY degradation.^20^ Therefore, the carboxylbenzyl (Cbz) or benzyl (Bn) groups were employed to protect the amino acids bearing nucleophilic side chains. The advantages of these two protecting groups are as follows: (1) the Cbz and Bn deprotection can be achieved under mild, neutral conditions in quantitative yields; (2) this strategy eliminates the formation of byproducts during the linker-free conjugation reaction; (3) the generated toluene product from Cbz or Bn deprotection is easily removed via vacuum evaporation; (4) Cbz or Bn protection can be applied to a variety of *O*,*N*-nucleophilic side chains; and (5) deprotection of the Cbz or Bn group does not affect the Boc group on the nitrogen adjacent to boron. Catalytic hydrogenation was performed to deprotect the Cbz or Bn side group(s) of the BODIPY derivatives (Scheme 1). The disappearance of the benzyl group (2H at ca. 5.0 ppm and 5H at ca. 7.5 ppm in CDCl_3_) could be clearly seen by ^1^H NMR, as shown in the Supporting Information. This methodology was successfully applied to the Asp-, Arg-, His-, and Ser-BODIPY conjugates, producing the corresponding **a–c** analogues. However, in the case of the Lys-BODIPY derivatives **9A–C**, upon the removal of the Cbz protecting group, the products decomposed within a few hours at room temperature, likely due to a nucleophilic attack of the amine upon the boron atom. The Arg-BODIPY was also observed to slowly decompose within 24 h; however, the His-BODIPY and all other derivatives bearing acidic, polar, and nonpolar amino acid side chains are stable in both solution and solid forms for at least 1 week at room temperature.

Presumably, the reaction of BODIPY **1** in the presence of boron trichloride proceeds via either the formation of a boronium cation intermediate,^41^ followed by the nucleophilic attack of the amino acid carboxylate group on boron to produce product **A**, or via the in situ formation of a Cl_2_-BODIPY intermediate^43−45^ leading to products **B** and **C**.

**Scheme 2:**
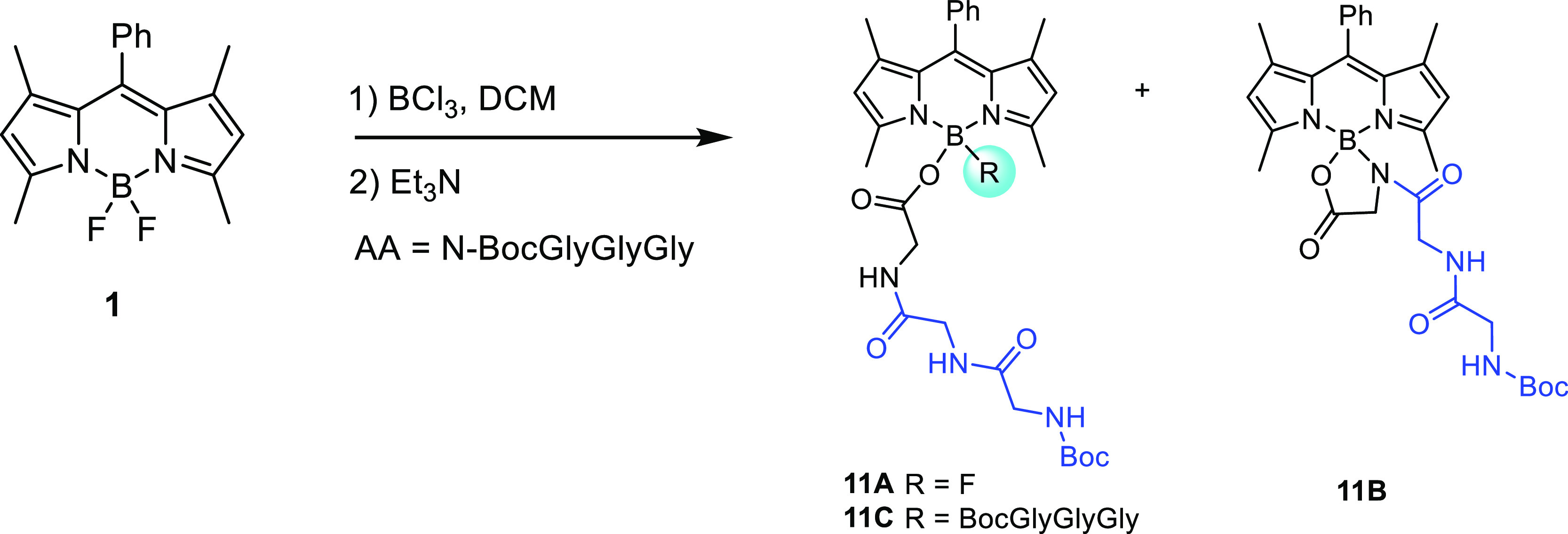
Synthesis of (Gly)_3_–BODIPY Conjugates

The above methodology was extended to the synthesis of BODIPY derivative **8** bearing an azide-functionalized lysine residue suitable for further conjugation with alkyne-containing compounds via click chemistry. In addition, the tripeptide, *N*-Boc-GlyGlyGly was also prepared using this methodology, as shown in Scheme 2, producing *N*,*O*-bidentate (Gly)_3_-BODIPY **11B** as the major product in 26.0% yield, followed by **11C** in 19.5% yield.

The structures of all α-amino acid-BODIPY conjugates were confirmed by ^1^H, ^13^C, ^11^B-NMR, and high-resolution mass spectroscopy (HRMS) (see the Supporting Information). In all cases, the 9H in the Boc protecting groups can be seen at 1.4 ppm in the ^1^H NMR spectra in the CDCl_3_ solvent. The characteristic methyl protons at the 3,5-positions of the BODIPYs appear at ca. 2.2 ppm, while the protons at the 1,7-positions are shifted upfield to 1.3 ppm due to the shielding effect of the 8-phenyl ring. In the ^1^H NMR, the protons at the α-carbon of the amino acid moiety are in the range of 4.5–5.0 ppm in CDCl_3_, while the protons at the β-carbons are in the range of 3.0–4.0 ppm, depending on the side chain. Interestingly, the three products, **A/a**, **B/b**, and **C/c**, could be easily distinguished by ^11^B-NMR as the spiro-(**B/b**) BODIPYs show one singlet at ∼1.9 ppm, while the mono-(**A/a**) BODIPYs show a doublet centered at ∼0.4 ppm and the di-(**C/c**) amino acid–BODIPYs show a singlet at ∼0.0 ppm.

### Computational Studies

Our previous computational and NMR studies^32^ showed that the spiro-Gly-BODIPY derivatives exist as two conformers (up and down) at room temperature, depending on the orientation of the Boc group with respect to the meso-phenyl, with the up-conformer being slightly more stable (Δ*G* < 3 kcal/mol). A similar trend is observed in the current series of amino acid-BODIPY derivatives. All the calculated data shown in Table S1 of the Supporting Information are for the up-conformers of the respective *N*,*O*-bidentate BODIPYs **B**. The mono-(**A**) and di-(**C**) amino acid–BODIPYs also exist in the form of two conformers with small energy differences (Δ*G* < 5 kcal/mol). All data discussed below refer to the more stable conformers.

Analysis of the calculated relative Gibbs energies of BODIPYs **2a–7a**, **2b–7b**, and **2c–7c** in dichloromethane demonstrates that the mono-substituted **a** is the most stable of the three forms, while the conformationally restricted spiro-compound **b** and the di-substituted **c** have similar Gibbs energies (see Table S1). The effect of the side chain is small and the energy differences range between 14 and 19 kcal/mol. However, experimentally BODIPYs bearing amino acids with strongly basic side chains, such as Arg and Lys, slowly decomposed in solution over a 24 h period, while all others, including His-BODIPY, were stable in solutions for over 1 week at room temperature. These results are consistent with previous observations of BODIPY instability under strongly basic conditions, such as in the presence of ^*t*^BuOK^46^ or NH_4_OH.^18^ The lower stability of the di-substituted BODIPYs **c** compared with the mono-derivatives **a** is mostly due to entropy effects. On the other hand, the lower stability of the *N*,*O*-bidentate BODIPYs **b** is probably due to their relatively weaker B–N(Boc) bond compared with the B–O and B–F bonds. The B–N(Boc) bonds range between 1.541 and 1.547 Å with **3b** having the longest bond of the series (see Table S1). The B–N and B–O bond lengths are similar in all the investigated *N*,*O*-bidentate spiro-BODIPYs and do not change significantly when different amino acids are introduced. However, for all compounds in the series, the B–N(dipyrrin) bonds in the spiro-BODIPYs **b** are consistently 0.01–0.02 Å longer than the ones in the mono **a** and di-substituted **c** BODIPYs. Our previous studies on the stability of BODIPYs^16^ suggest that the spiro-conjugates will therefore be slightly less stable than the di-substituted compounds. Furthermore, our calculations show that the *N*,*O*-bidentate **b** compounds are consistently more polar than the mono **a** and di-substituted **c** analogues throughout the entire series, with **2b** spiro-Asp being the most polar of all (see Table S1). The higher polarity of the *N*,*O*-bidentate spiro-compounds explains their observed higher solubility in polar solvents.

### Spectroscopic Properties

The spectroscopic properties of the selected conformationally restricted BODIPYs **2b,c**, **3b,c**, **4b,c**, **5B,C**, **6B,C**, and **7b,c** were measured and calculated in acetonitrile. The experimental results are shown in Table 1, and the calculated parameters are shown in the Supporting Information, Table S2. The bidentate spiro-(**B**) and the di-(**C**) amino acid-BODIPY derivatives have similar absorption and fluorescence profiles, with maximum absorption and emission bands centered at ca. 503 and 513 nm, respectively. These maximum absorption and emission wavelengths are slightly red-shifted (4–5 nm) compared with the parent BF_2_-BODIPY **1**. This shift is in agreement with the performed TD-DFT calculations (Table S2). The predicted red shift is 3–5 nm and is due to the slightly smaller HOMO–LUMO gap for the α-amino acid–BODIPYs.

**Table 1:** Spectroscopic Properties of α-Amino Acid–BODIPYs in Acetonitrile at Room Temperature^a^

BDP	λ_abs_ (nm)	log ε (M^–1^ cm^–1^)	λ_em_ (nm)	Φ_f_^b^	Stokes’ shift (nm)
**1**	498	4.92	509	0.61	11
**2b**	503	4.56	512	0.99	9
**2c**	503	4.80	515	0.98	12
**3b**	503	4.58	513	0.83	10
**3c**	503	4.32	513	0.90	10
**4b**	503	4.83	514	0.92	11
**4c**	503	4.76	514	0.89	11
**5B**	503	4.71	513	0.82	10
**5C**	503	4.78	513	0.93	10
**6B**	502	4.85	513	0.92	11
**7b**	503	4.43	512	0.95	9
**7c**	502	4.81	513	0.94	11

a According to our TD-DFT M06-2X/6-31+G(d,p) calculations in acetonitrile, the leading transition is HOMO → LUMO. All calculated data can be found in Table S2, Supporting information.

b Fluorescein (0.91 in 0.1 M NaOH) was used as the standard.

**Figure 1:**
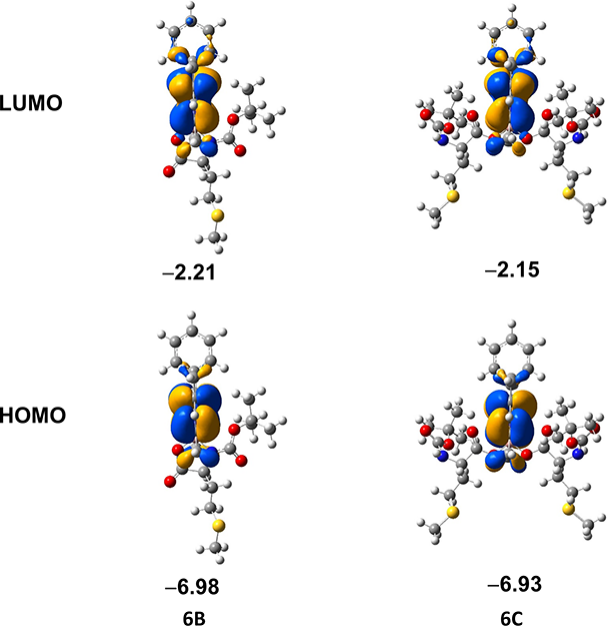
Frontier orbitals of BODIPYs **6B** and **6C**. Orbital energies in eV. The frontier orbitals of all conformers of all BODIPYs studied are given the Supporting Information (Figure S103).

The similar absorption and emission profiles observed for this series of α-amino acid–BODIPYs are consistent with the calculated similar HOMO–LUMO energies and gaps, as shown in Figure 1 and the Supporting Information, Figure S103. Figure 1 represents the frontier orbitals for the *N*,*O*-bidentate spiro and di-substituted BODIPYs **6B** and **6C**. Both HOMO and LUMO are localized on the BODIPY core with no significant contribution from the amino acid nor the Boc protecting group. This is also consistent with our previous findings^32^ and is observed for all other α-amino acid–BODIPYs in the series. The performed TD-DFT calculations show that HOMO → LUMO is the leading transition for all BODIPYs studied. Given the fact that the forms and energies of these two orbitals are essentially independent of the amino acid, it is not surprising that the maximum absorption and emission wavelengths are very similar among the entire series.

Remarkably, all newly synthesized α-amino acid–BODIPYs display higher fluorescence quantum yields (Φ ∼ 0.9) compared with the parent BF_2_–BODIPY **1** (Φ = 0.61), making them promising candidates for fluorescence labeling of bioactive peptides. As also suggested by our previous findings,^32^ this result is probably due to the increased rigidity around the boron center in these conformationally restricted BODIPYs, which minimizes vibrations in-and-out of the dipyrrin plane. Interestingly, the calculated oscillator strengths of all α-amino acid–BODIPYs are smaller than that of parent BF_2_-BODIPY **1**. However, the increased rigidity around the boron center most likely reduces the nonradiative vibrational relaxation, resulting in increased quantum yields.

### Cellular Properties

#### Cytotoxicity

The cytotoxicity of the selected conformationally restricted α-amino acid–BODIPY **2b,c**, **3b,c**, **4b,c**, **5B**, **6B**, and **7b** were evaluated in human carcinoma HEp2 cells exposed to the increasing concentrations of each BODIPY up to 200 μM; the results are shown in the Supporting Information, Figures S104 and S105 and summarized in Table 2. Among the series (Figure S105), the spiro-Arg **3b** and spiro-His **4b** are the most toxic BODIPYs with IC_50_ values of 22 and 23 μM, respectively. These results indicate that both a spiro-5-membered ring on boron (as often observed in boron-based therapeutics, such as spiro-borates), and the presence of basic amino acids, such as Arg and His, increase the cytotoxicity of the compound. Indeed, a 3–5-fold increase in cytotoxicity was observed for the spiro-BODIPYs in comparison with the corresponding di-amino acid derivatives (Figure S105), possibly due to the more rigid *N*,*O*-bidentate spiro-ring structure. On the other hand, basic amino acids such as Arg and His are capable of interacting with negatively charged groups on cell membranes and proteins, likely enhancing their cellular uptake and cytotoxicity. Previous studies of histidine-containing peptides showed a 2–8-fold increase in cytotoxicity as the solution pH decreased from 7.4 to 5.5,^47^ suggesting that His-based compounds can be useful therapeutics. All the other spiro-BODIPYs evaluated bearing acidic (Asp), polar (Tyr), and nonpolar side chains showed low cytotoxicity with calculated IC_50_ values >142 μM.

**Table 2:** Cytotoxicity of BODIPYs **2b**,**c**, **3b**,**c**, **4b**,**c**, **5B**, **6B**, and **7b** in HEp2 Cells Using the Cell Titer Blue Assay

Compound	Cytotoxicity (μM)
**2b**	58
**2c**	>200
**3b**	22
**3c**	104
**4b**	23
**4c**	68
**5B**	>200
**6B**	>200
**7b**	142

### Cellular Uptake and Intracellular Localization

**Figure 2:**
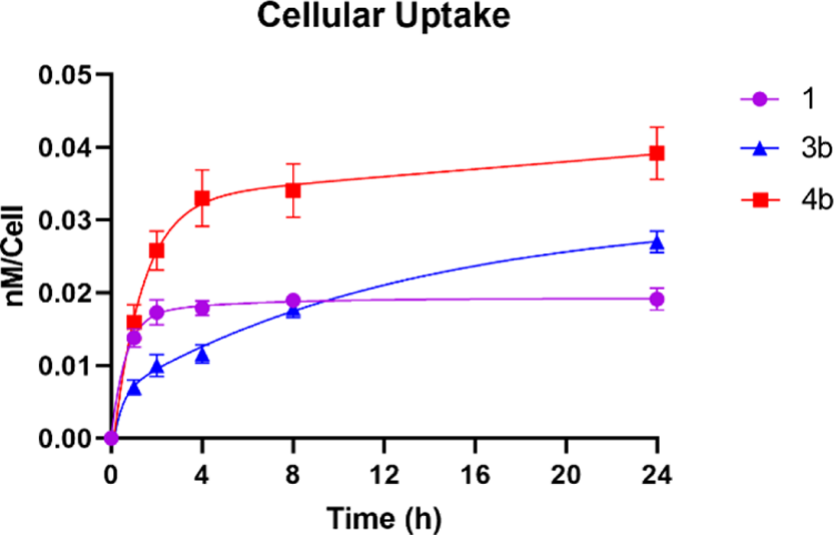
Time-dependent cellular uptake of BODIPYs **1** (purple), **3b** (blue), and **4b** (red) at 10 μM in human HEp2 cells.

The time-dependent cellular uptake of the most cytotoxic basic α-amino acid BODIPYs **3b** and **4b** and of the parent BODIPY **1** at a nontoxic concentration of 10 μM were evaluated in human carcinoma HEp2 cells (Figure 2). The basic amino acids Arg and His are able to interact with phosphate groups on plasma membranes, which enhances their cellular uptake. BODIPY **4b** containing His showed the highest uptake, about 2-fold that of the parent BODIPY **1** at times >2 h. The lower uptake observed for the Arg-BODIPY **3b**, compared with **4b** at all the times investigated, might in part be a result of its relatively lower stability due to the greater basicity of the guanidinium group. Although at times <8 h, BODIPY **3b** showed a lower uptake than parent BODIPY **1**, the positively charged guanidinium group in **3b** favored its continuous uptake over time via interaction with the negatively charged plasma membrane, thus leading to an enhanced uptake at 24 h relative to BODIPY **1**.^48,49^

**Figure 3:**
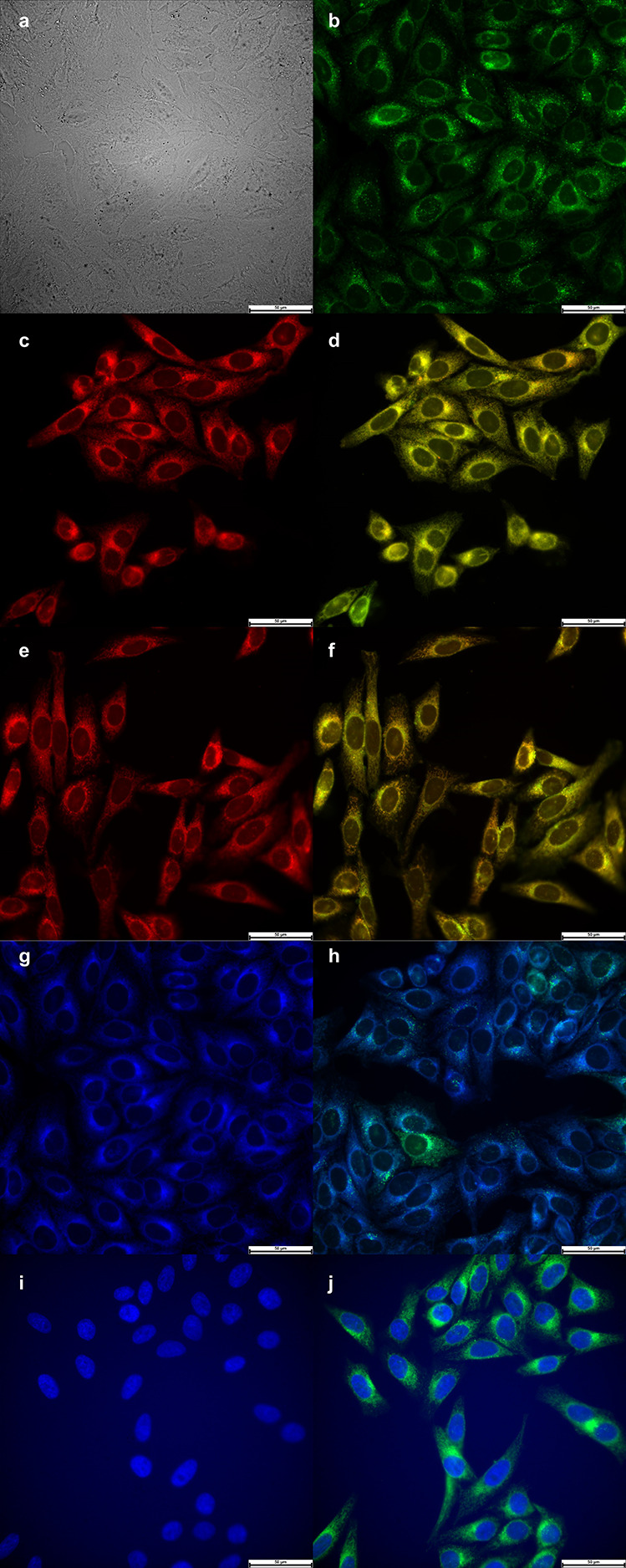
Sub-cellular localization of BODIPY **3b** in HEp2 cells at 10 μM after 6 h of incubation period. (a) Bright field; (b) fluorescence of BODIPY **3b**; (c) LysoTracker deep red; (e) MitoTracker deep red; (g) ER Tracker blue/white; (i) Hoechst 33342; and (d,f,h,j) overlays of tracers with BODIPY fluorescence. Scale bar: 50 μm.

**Figure 4:**
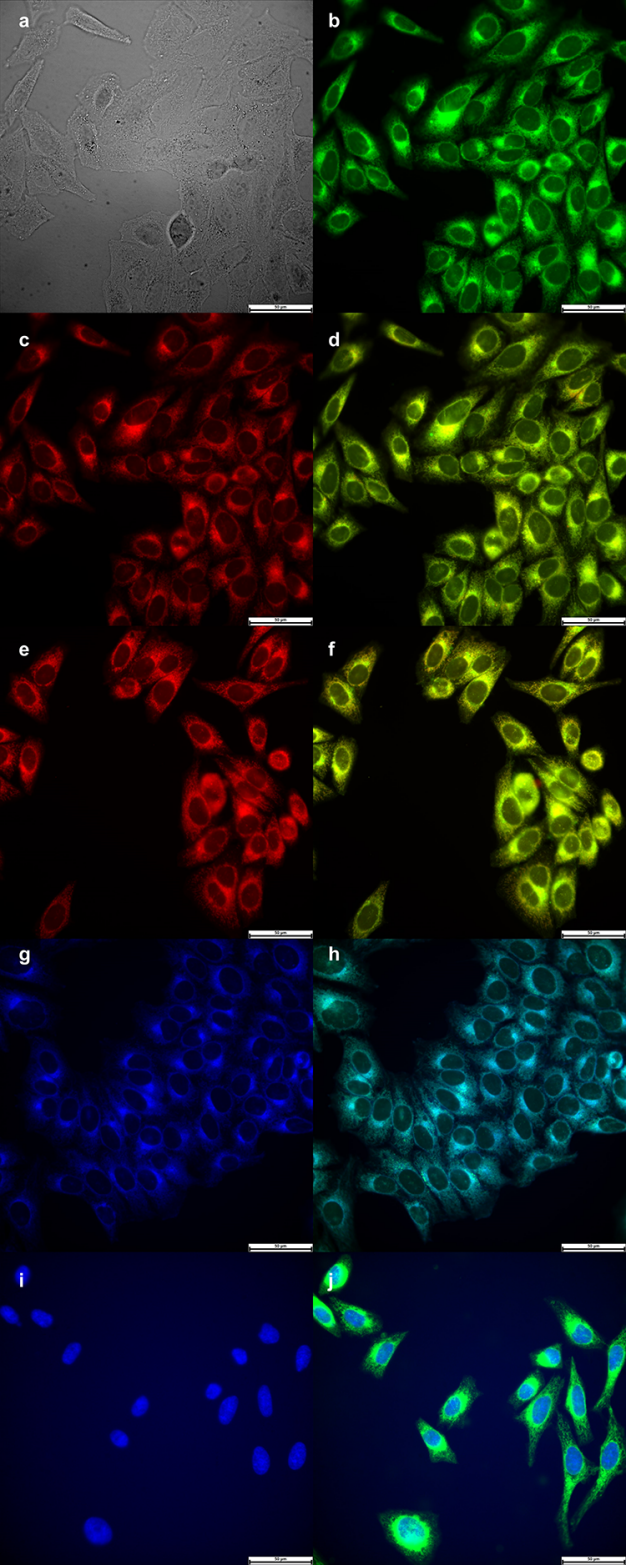
Sub-cellular localization of BODIPY **4b** in HEp2 cells at 10 μM after 6 h incubation period. (a) Bright field; (b) fluorescence of BODIPY **4b**; (c) LysoTracker deep red; (e) MitoTracker deep red; (g) ER Tracker blue/white; (i) Hoechst 33342; and (d,f,h,j) overlays of tracers with BODIPY fluorescence. Scale bar: 50 μm.

The sub-cellular localization of BODIPYs **3b** (Figure 3) and **4b** (Figure 4) were also investigated by fluorescence microscopy upon the exposure of the HEp2 cells to 10 μM of each BODIPY for 6 h. Overlay experiments using the organelle-specific fluorescent probes LysoTracker deep red (lysosomes), MitoTracker deep red (mitochondria), ER Tracker blue/white (ER), and Hoechst 33342 (nuclei) were conducted to evaluate the preferential sites of BODIPY localization. The results obtained are shown in Figures 3 and 4. Both BODIPYs **3b** and **4b** are found to localize in the ER, mitochondria, and lysosomes. We have previously reported that BODIPY **1** accumulates preferentially in the ER, Golgi, and lysosomes.^19^ Our results suggest that BODIPYs bearing positively charged amino acids (Arg, His) also localize in mitochondria, which might account for their observed higher cytotoxicity.

## Conclusions

A series of conformationally restricted α-amino acid–BODIPYs, bearing basic (His, Lys, and Arg), acidic (Asp), polar (Tyr, Ser), and nonpolar (Met) side chains, were synthesized in moderate yields from an in situ prepared BCl_2_–BODIPY and *N*(Boc)-l-amino acids, at room temperature. The stability of these conformationally restricted BODIPYs depends on the amino acid binding mode and the basicity of its side chain, with the acidic, polar, and moderate basic (i.e., His) mono-linear amino acid–BODIPY derivatives being the most stable. The calculated relative Gibbs energies show that the *N*,*O*-bidentate spiro-BODIPYs are slightly less stable than the corresponding di-amino acid derivatives due to the weaker B–N(Boc) bond compared with the B–O bond.

The conformationally restricted amino acid–BODIPYs display similar absorption and emission properties, slightly red-shifted relative to the parent BF_2_–BODIPY due to their slightly smaller HOMO–LUMO gap, and TD-DFT calculations suggest that HOMO → LUMO is the leading transition. Furthermore, all conformationally restricted amino acid–BODIPYs showed enhanced fluorescence quantum yields (Φ ∼ 0.9) compared with the parent BF_2_–BODIPY (Φ = 0.61) due to the increased rigidity around the boron center, which minimizes vibrations in-and-out of the dipyrrin plane.

Investigations in human HEp2 cells showed that the *N*,*O*-bidentate spiro-Arg and spiro-His are the most cytotoxic (IC_50_ ∼ 22 μM), 3–5-fold more toxic than their corresponding di-amino acid BODIPY derivatives. The basic amino acid–BODIPYs are able to interact with negatively charged groups on plasma cell membranes showing an enhanced cellular uptake relative to the parent BF_2_–BODIPY at 24 h. The preferential sites of intracellular localization for the most cytotoxic basic amino acid–BODIPYs were found to be the ER, mitochondria, and lysosomes, which might account for their observed higher cytotoxicity.

## Experimental Section

### General

Commercial grade chemical reagents were purchased from VWR or Sigma-Aldrich and used without further purification. Liquid column chromatography with silica gel (60 Å, 230–400 mesh) or preparative TLC plates (60 Å, 20 × 20 cm^2^, 210–270 μm) were used for all the purifications. NMR spectra were collected using a Bruker AV-400 or AV-500 spectrometer at 300 K (operating at 400 or 500 MHz for ^1^H, 126 MHz for ^13^C NMR, and 128 MHz for ^11^B NMR) in CDCl_3_ (7.26 ppm for ^1^H and 77.0 ppm for ^13^C), CD_2_Cl_2_ (5.30 ppm for ^1^H and 53.4 ppm for ^13^C), CD_3_OD (3.35 ppm for ^1^H and 49.3 ppm for ^13^C), and BF_3_·OEt_2_ was set as a reference (0.00 ppm) for ^11^B NMR. High-resolution mass spectra were collected at the LSU Department of Chemistry Mass Spectrometry Facility on an Agilent 6230-B ESI-TOF spectrometer in the positive mode. UV–vis absorption spectra were collected on a Varian Cary spectrophotometer. The fluorescence spectra were obtained using a PerkinElmer LS55 spectrophotometer. Emission spectra (λ_ex_ = 470 nm) were recorded in quartz cells. Relative fluorescence quantum yields (Φ_f_) were calculated using Fluorescein (Φ_f_ = 0.91 in 0.1 M NaOH) as the standard for all compounds using the equation
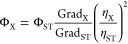
where Φ_X_ and Φ_ST_ are the quantum yields of the sample and standard, Grad_X_ and Grad_ST_ are the gradients from the plot of the integrated fluorescence intensity versus absorbance, and η represents the refractive index of the solvent. BODIPY **1** was prepared as previously reported in the literature.^41^ Larger scale syntheses of **A**–**C** products have been previously reported.^33^

### Computational Methods

The geometries of all compounds and complexes were optimized without symmetry constraints using the B3LYP/6-31+G(d,p) level.^50−52^ The solvent effects were taken into account using the polarized continuum model.^53,54^ The stationary points on the potential energy surface were confirmed with frequency calculations. The UV–vis absorption data were calculated using the TD-DFT method^55^ All calculations were performed using the Gaussian 09 program package.^56^

### Cell Culture

#### Cytotoxicity

All the cell culture media and reagents were purchased from VWR and fisher scientific. The human carcinoma HEp2 cells were purchased from ATCC and maintained in a MEM containing 5% FBS and 1% penicillin/streptomycin antibiotic. The cells were then sub-cultured and maintained twice weekly. Cell toxicity was evaluated using the CellTiter-Blue (Promega) assay.

The HEp2 cells were plated at 7500–10 000 cells per well in a Costar 96-well plate and allowed to grow for 24–48 h. BODIPY samples were dissolved in 100% DMSO to prepare 32 mM stock solutions. Then, a 400 μM working solution was prepared for each sample. Through a 2-fold dilution procedure, HEp2 cells were exposed to concentrations of 0, 6.25, 12.5, 25, 50, 100, and 200 μM solutions and incubated for 24 h at 37 °C. The working solutions were then removed, and the cells were washed with 1 × PBS buffer triple times. The cells were exposed to the medium containing 20% CellTiterBlue and incubated for 4 h at 37 °C. The viability of the cells is measured by reading the fluorescence of the medium at 570/615 nm using a BMG FLUOstarOptima microplate reader. The fluorescence signal of the untreated cells in media only was normalized to 100%.

#### Cellular Uptake

Costar 96-well plates were prepared as described above. Each BODIPY solution (10 μM) was added to the cells and incubated for 0, 1, 2, 4, 8, and 24 h. At the end of the incubation period, the BODIPY solution was removed, and the cells were washed with 1 × PBS buffer triple times, followed by solubilizing the cells with 0.25% Triton X-100 in 1 × PBS buffer. The compound standard curve of each BODIPY was prepared by using BODIPY solutions with concentrations of 0, 0.31, 0.63, 1.25, 2.5, 5, and 10 μM in 1 × PBS buffer containing 0.25% X-100. The standard curve of the cell number was prepared using 10 000, 20 000, 40 000, 60 000, 80 000, and 100 000 cells per well and quantified by using a CyQuant Cell Proliferation Assay. The BODIPY concentration in cells at the end of each incubation period was determined using a BMG FLUOstar Optima microplate reader at the excitation wavelength and emission wavelength of each BODIPY. The time-dependent cellular uptake was evaluated as nM/cell.

#### Microscopy

The human HEp2 cells were incubated in a 35 mm tissue culture dish (CELLTREAT) and allowed to grow overnight. The cells were exposed to each compound at a concentration of 10 μM and incubated for 6 h (5% CO_2_, 37 °C). The following organelle trackers (Invitrogen) were added to the cells: ER Tracker Blue/White (1 mM), Hoechst 33342 (16 mM), MitoTracker deep red (1 μM), and LysoSensor deep red (1 μM). The cells were incubated with each compound and trackers for half an hour, washed with PBS solution three times, and followed by fixing with 4% paraformaldehyde before imaging. A Leica DM6B upright microscope equipped with a 40 × water immersion objective, and DAPI, GFP, and Texas Red filter cubes (Chroma Technologies) were used to acquire the images.

### Synthesis

#### General Procedure for the Preparation of α-Amino Acid–BODIPY Derivatives

BODIPY **1** (50 mg, 0.154 mmol) was dissolved in 5 mL of dry dichloromethane, followed by the addition of 2 equivalents of BCl_3_ (1 M in toluene, 0.3 mL, 0.308 mmol). The reaction was stirred for 1 h at room temperature. Subsequently, a few drops of dry triethylamine were added to the reaction mixture. The N-protected amino acids (4 equivalents) were dissolved in a vial with dry dichloromethane and a few drops of dry triethylamine. The amino acid solutions were then added dropwise and stirred at room temperature for 1 h. The reaction mixture was then poured into a saturated NaHCO_3_ solution and extracted with dichloromethane (10 mL × 3). The organic layers were combined and dried over anhydrous Na_2_SO_4_. The organic solvent was removed under reduced pressure, and the residue was purified by column chromatography on silica gel and recrystallized to afford the desired BODIPYs.

BODIPY **2** was synthesized using *N*-Boc-l-aspartic acid 4-benzyl ester (199.5 mg, 0.616 mmol) and purified by column chromatography using ethyl acetate/hexane (1:4). Compound **2A** was obtained as an orange solid (8.0 mg, 0.013 mmol, 8.4%): ^1^H NMR (400 MHz, CDCl_3_): δ 7.51–7.27 (m, 10H), 5.93 (d, *J* = 10.2 Hz, 2H), 5.51 (d, *J* = 7.5 Hz, 1H), 5.08 (q, *J* = 12.4 Hz, 2H), 4.45 (bs, 1H), 3.11 (dd, *J* = 16.4 Hz, 1H), 2.92 (dd, *J* = 16.8, 5.0 Hz, 1H), 2.48 (s, 3H), 2.36 (s, 3H), 1.41 (s, 9H), 1.38 (s, 6H). ^13^C{^1^H}NMR (126 MHz, CDCl_3_): δ 170.9, 170.6, 170.5, 155.4, 155.1, 154.7, 143.4, 143.1, 142.2, 135.7, 135.0, 132.2, 129.2, 128.9, 128.9, 128.5, 128.3, 128.2, 127.9, 121.3, 79.5, 66.5, 51.0, 37.1, 28.3, 14.6, 14.5. ^11^B NMR (128 MHz, CDCl_3_): δ 0.40 (d, *J* = 23.2 Hz). HRMS (ESI-TOF) *m*/*z* [M + H]^+^ calcd for C_35_H_40_BFN_3_O_6_, 628.2995; found, 628.3001. Compound **2B** was obtained as an orange solid (25.0 mg, 0.041 mmol, 26.6%): ^1^H NMR (400 MHz, CDCl_3_): δ 7.55–7.28 (m, 10H), 6.00 (d, *J* = 3.1 Hz, 2H), 5.28–5.18 (m, 2H), 4.97 (s, 1H), 3.19 (dd, *J* = 15.7, 4.8 Hz, 1H), 3.00 (dd, *J* = 15.8, 6.8 Hz, 1H), 2.34 (s, 3H), 2.30 (s, 3H), 1.39 (s, 3H), 1.37 (s, 3H), 1.19 (s, 9H). ^13^C{^1^H} NMR (126 MHz, CDCl_3_): δ 175.8, 171.1, 156.0, 155.5, 154.6, 143.8, 143.5, 142.0, 136.1, 134.9, 133.2, 132.9, 129.6, 129.2, 129.1, 128.5, 128.4, 128.4, 128.2, 128.1, 127.4, 122.7, 122.7, 78.7, 66.8, 57.0, 36.5, 28.3, 16.4, 15.3, 14.7. ^11^B NMR (128 MHz, CDCl3): δ 1.87 (s). HRMS (ESI-TOF) m/z [M + H]^+^ calcd for C_35_H_39_BN_3_O_6_, 608.2933; found, 608.2945. Compound **2C** was obtained as an orange solid (41.0 mg, 0.044 mmol, 28.6%): ^1^H NMR (400 MHz, CDCl_3_): δ 7.50–7.28 (m, 15H), 5.89 (s, 2H), 5.50 (d, *J* = 7.4 Hz, 2H), 5.14–5.03 (m, 4H), 4.47 (s, 2H), 3.15–3.03 (m, 2H), 3.00–2.88 (m, 2H), 2.30 (s, 6H), 1.41 (s, 18H), 1.38 (s, 6H). ^13^C{^1^H} NMR (126 MHz, CDCl_3_): δ 170.8, 170.6, 155.4, 153.9, 143.2, 135.6, 135.1, 132.6, 129.0, 128.9, 128.6, 128.3, 128.2, 121.5, 79.6, 66.6, 50.9, 36.9, 28.4, 14.7, 14.5. ^11^B NMR (128 MHz, CDCl_3_): δ 0.07 (s). HRMS (ESI-TOF) *m*/*z* [M + Na]^+^ calcd for C_51_H_59_BNaN_4_O_12_, 953.4123; found, 953.4166.

BODIPY **3** was synthesized using *N*_α_-Boc-*N*_δ_,*N*_ω_-di-carboxybenzyl-l-arginine (173.5 mg, 0.617 mmol) and purified by column chromatography using ethyl acetate/hexane (1:4) for elution. Compound **3A** was obtained as an orange solid (26.0 mg, 0.031 mmol, 20.0%): ^1^H NMR (400 MHz, CDCl_3_): δ 9.46 (s, 1H), 9.27 (s, 1H), 7.49–7.44 (m, 3H), 7.42–7.33 (m, 10H), 7.31–7.27 (m, 2H), 5.93 (s, 1H), 5.83 (s, 1H), 4.24 (d, *J* = 6.2 Hz, 1H), 4.05 (dd, *J* = 12.8, 7.5 Hz, 1H), 3.95 (d, *J* = 8.2 Hz, 1H), 2.44 (s, 3H), 2.40 (s, 3H), 1.92–1.82 (m, 1H), 1.58–1.46 (m, 2H), 1.39 (s, 9H), 1.37 (s, 3H), 1.35 (s, 3H). ^13^C{^1^H} NMR (126 MHz, CDCl_3_): δ 172.0, 164.0, 160.6, 156.0, 155.3, 154.7, 143.3, 143.2, 142.3, 136.9, 135.0, 134.8, 132.3, 129.1, 128.9, 128.9, 128.8, 128.8, 128.4, 128.3, 128.2, 128.0, 127.9, 127.8, 121.3, 79.3, 68.9, 67.1, 54.2, 44.3, 31.6, 30.2, 29.7, 28.4, 24.9, 22.6, 14.6, 14.5, 14.4, 14.1. ^11^B NMR (128 MHz, CDCl_3_): δ 0.36 (d, *J* = 21.5 Hz). HRMS (ESI-TOF) *m*/*z* [M + H]^+^ calcd for C_46_H_53_BFN_6_O_8_, 847.4004; found, 847.3984. Compound **3B** was obtained as an orange solid (26.0 mg, 0.032 mmol, 20.4%): ^1^H NMR (400 MHz, CDCl_3_): δ 9.49 (s, 1H), 9.30 (s, 1H), 7.54–7.46 (m, 3H), 7.45–7.38 (m, 6H), 7.38–7.29 (m, 4H), 7.29–7.26 (m, 1H), 7.25–7.20 (m, 1H), 5.94 (s, 2H), 5.26 (s, 2H), 5.15 (s, 2H), 4.15 (d, *J* = 8.1 Hz, 2H), 2.27 (s, 3H), 2.13 (s, 3H), 1.37 (s, 6H), 1.18 (s, 9H). ^13^C{^1^H} NMR (126 MHz, CDCl_3_): δ 177.0, 164.0, 160.8, 156.1, 154.2, 143.7, 143.0, 141.8, 137.2, 135.0, 134.9, 133.1, 132.9, 129.5, 129.1, 129.0, 128.8, 128.6, 128.4, 128.4, 128.2, 127.9, 127.6, 127.5, 122.7, 122.5, 78.3, 68.8, 67.1, 60.0, 44.6, 31.6, 29.5, 28.3, 27.5, 22.6, 16.5, 15.0, 14.7, 14.6, 14.1. ^11^B NMR (128 MHz, CDCl_3_): δ 1.79(s). HRMS (ESI-TOF) *m*/*z* [M + H]^+^ calcd for C_46_H_52_BN_6_O_8_, 827.3942; found, 827.3970. Compound **3C** was obtained as an orange solid (28 mg, 0.021 mmol, 13.2%): ^1^H NMR (400 MHz, CDCl_3_): δ 9.46 (s, 2H), 9.26 (s, 2H), 7.47–7.27 (m, 25H), 5.79 (s, 2H), 5.21 (s, 4H), 5.13 (s, 4H), 4.31–4.24 (m, 2H), 4.05 (dd, *J* = 13.3, 7.8 Hz, 2H), 3.95 (d, *J* = 5.4 Hz, 2H), 2.33 (d, *J* = 4.9 Hz, 2H), 2.30 (s, 6H), 1.89 (bs, 2H), 1.59–1.48 (m, 4H), 1.39 (s, 18H), 1.34 (s, 6H). ^13^C{^1^H} NMR (126 MHz, CDCl_3_): δ 172.2, 164.0, 160.6, 155.9, 155.3, 153.5, 143.1, 137.0, 135.0, 134.7, 132.7, 128.9, 128.8, 128.8, 128.4, 128.3, 128.2, 128.0, 127.8, 121.4, 79.4, 68.9, 67.0, 54.2, 44.3, 31.6, 30.0, 28.4, 24.9, 22.6, 14.6, 14.1. ^11^B NMR (128 MHz, CDCl_3_): δ −0.06 (s). HRMS (ESI-TOF) *m*/*z* [M + H]^+^ calcd for C_73_H_86_BN_10_O_16_, 1369.6328; found, 1369.6315.

BODIPY **4** was synthesized using *N*-Boc-l-histidine (240.2 mg, 0.617 mmol) and purified by column chromatography using ethyl acetate/hexane (1:1) for elution. Compound **4A** (trace, not isolated). Compound **4B** was obtained as an orange solid (27.0 mg, 0.040 mmol, 26.0%): ^1^H NMR (400 MHz, CDCl_3_): δ 8.13 (s, 1H), 7.57–7.34 (m, 10H), 7.24 (bs, 1H), 6.00 (d, *J* = 15.3 Hz, 2H), 5.39 (s, 2H), 4.63 (d, *J* = 7.5 Hz, 1H), 3.49 (d, *J* = 14.2 Hz, 1H), 3.18 (dd, *J* = 14.5, 9.4 Hz, 1H), 2.47 (s, 3H), 2.24 (s, 3H), 1.38 (s, 6H), 1.19 (s, 9H). ^13^C{^1^H} NMR (126 MHz, CDCl_3_): δ 176.4, 156.1, 155.8, 148.8, 143.8, 143.2, 141.9, 141.5, 136.7, 135.0, 134.2, 133.1, 132.9, 129.5, 129.2, 129.1, 129.0, 128.8, 128.7, 128.4, 127.5, 122.7, 122.6, 114.4, 78.5, 69.6, 59.8, 31.6, 30.6, 28.3, 22.7, 16.7, 15.3, 14.7, 14.6, 14.1. ^11^B NMR (128 MHz, CDCl_3_): δ 1.81 (s). HRMS (ESI-TOF) *m*/*z* [M + H]^+^ calcd for C_38_H_41_BN_5_O_6_, 674.3151; found, 674.3145. Compound **4C** was obtained as an orange solid (18.5 mg, 0.017 mmol, 11.3%): ^1^H NMR (400 MHz, CDCl_3_): δ 8.01 (s, 2H), 7.48–7.34 (m, 15H), 7.12 (s, 2H), 5.77 (s, 2H), 5.44 (d, *J* = 8.0 Hz, 2H), 5.37 (s, 4H), 4.53 (s, 2H), 3.20 (d, *J* = 5.0 Hz, 1H), 3.16 (d, *J* = 4.5 Hz, 1H), 3.05 (d, *J* = 6.0 Hz, 1H), 3.01 (d, *J* = 5.8 Hz, 1H), 2.24 (s, 6H), 1.36 (s, 18H), 1.34 (s, 6H). ^13^C{^1^H} NMR (126 MHz, CDCl_3_): δ 171.6, 155.3, 148.5, 139.8, 136.6, 135.1, 134.1, 132.6, 129.2, 128.9, 128.7, 128.3, 121.2, 114.4, 79.3, 69.7, 53.6, 30.7, 28.3, 14.7, 14.6. ^11^B NMR (128 MHz, CDCl_3_): δ −0.01 (s). HRMS (ESI-TOF) *m*/*z* [M + Na]^+^ calcd for C_57_H_63_BN_8_NaO_12_, 1085.4560; found, 1085.4526.

BODIPY **5** was synthesized using *N*-Boc-l-tyrosine (173.5 mg, 0.617 mmol) purified by column chromatography using ethyl acetate/hexanes (1:2) for elution. Compound **5A** was obtained as an orange solid (4.2 mg, 0.007 mmol, 4.6%): ^1^H NMR (400 MHz, CDCl_3_): δ 7.49–7.43 (m, 3H), 7.41–7.35 (m, 1H), 7.30–7.27 (m, 1H), 6.83 (d, *J* = 8.4 Hz, 2H), 6.56 (d, *J* = 8.0 Hz, 2H), 5.97 (s, 1H), 5.96 (s, 1H), 5.00 (d, *J* = 8.3 Hz, 1H), 4.47 (dd, *J* = 13.7, 5.7 Hz, 1H), 3.12 (dd, *J* = 13.9, 5.5 Hz, 1H), 2.90 (dd, *J* = 13.7, 6.0 Hz, 1H), 2.46 (s, 3H), 2.41 (s, 3H), 1.38 (s, 6H), 1.36 (s, 9H). ^13^C{^1^H} NMR (126 MHz, CDCl_3_): δ 172.0, 155.3, 155.1, 154.2, 143.6, 143.2, 142.4, 134.9, 132.4, 130.4, 129.2, 128.9, 128.3, 127.8, 121.4, 121.2, 115.3, 79.5, 55.4, 37.5, 28.3, 21.1, 14.7, 14.5, 14.4, 14.2. ^11^B NMR (128 MHz, CDCl_3_): δ 0.45 (d, *J* = 18.3 Hz). HRMS (ESI-TOF) *m*/*z* [M + Na]^+^ calcd for C_33_H_37_BFNaN_3_O_5_, 608.2708; found, 608.2703. Compound **5B** was obtained as an orange solid (22.6 mg, 0.040 mmol, 25.9%): ^1^H NMR (400 MHz, CDCl_3_): δ 7.59–7.39 (m, 7H), 6.82–6.75 (m, 2H), 6.03 (s, 1H), 5.97 (s, 1H), 4.41–4.33 (m, 1H), 3.42–3.36 (m, 1H), 3.16 (m, 1H), 2.48 (s, 3H), 2.19 (s, 3H), 1.39 (s, 3H), 1.38 (s, 3H), 1.23 (s, 9H). ^13^C{^1^H} NMR (126 MHz, CDCl_3_): δ 177.0, 156.4, 155.9, 154.5, 143.8, 143.2, 141.8, 135.0, 133.0, 131.6, 131.0, 129.6, 129.2, 129.1, 128.4, 127.5, 122.7, 122.6, 115.3, 78.6, 53.8, 37.6, 28.4, 14.7, 14.6; ^11^B NMR (128 MHz, CDCl_3_): δ 1.82 (s). HRMS (ESI-TOF) *m*/*z* [M + H]^+^ calcd for C_33_H_36_BN_3_O_5_, 566.2827; found, 566.2823. Compound **5C** was obtained as an orange solid (10.0 mg, 0.012 mmol, 7.7%): ^1^H NMR (400 MHz, CDCl_3_): δ 7.44 (bs, 3H), 7.39–7.33 (m, 2H), 6.91 (d, *J* = 7.5 Hz, 4H), 6.60 (d, *J* = 7.6 Hz, 4H), 5.92 (s, 2H), 5.01 (d, *J* = 8.0 Hz, 2H), 4.58–4.43 (m, 2H), 3.14–2.82 (m, 4H), 2.23 (s, 6H), 1.37 (bs, 24H). ^13^C{^1^H} NMR (126 MHz, CDCl_3_): δ 172.2, 155.4, 155.1, 153.7, 143.4, 134.9, 132.6, 130.5, 129.0, 128.8, 128.2, 127.9, 121.4, 115.4, 79.8, 55.5, 37.6, 28.3, 14.7, 14.6; ^11^B NMR (128 MHz, CDCl_3_): δ −0.01 (s). HRMS (ESI-TOF) *m*/*z* [M + Na]^+^ calcd for C_47_H_55_BNaN_4_O_10_, 869.3912; found, 869.3925.

BODIPY **6** was synthesized using *N*-Boc-l-methionine (153.8 mg, 0.617 mmol), purified by preparative TLC plates using ethyl acetate/dichloromethane/hexanes (1:1:6) for elution. Compound **6A** (trace product, not isolated). Compound **6B** was obtained as an orange solid (19.1 mg, 0.036 mmol, 23.2%): ^1^H NMR (400 MHz, CD_2_Cl_2_): δ 7.51–7.47 (m, 3H), 7.31–7.27 (m, 1H), 7.24 (bs, 1H), 6.03 (s, 1H), 6.02 (s, 1H), 4.26 (dd, *J* = 8.6, 3.0 Hz, 1H), 3.01–2.94 (m, 1H), 2.92–2.84 (m, 1H), 2.36 (s, 3H), 2.21 (s, 3H), 2.15 (s, 3H), 1.38 (s, 3H), 1.37 (s, 4H), 1.16 (s, 9H). ^13^C{^1^H} NMR (126 MHz, CDCl_3_): δ 176.9, 156.1, 155.9, 154.2, 143.8, 143.2, 141.8, 134.9, 133.0, 132.9, 129.5, 129.1, 129.0, 128.3, 127.4, 122.7, 122.5, 78.4, 59.2, 32.4, 31.6, 28.2, 16.6, 15.3, 15.1, 14.7, 14.6; ^11^B NMR (128 MHz, CDCl_3_): δ 1.83 (s). HRMS (ESI-TOF) *m*/*z* [M + H]^+^ calcd for C_29_H_37_BN_3_O_4_S 534.2598; found, 534.2623. Compound **6C** was obtained as an orange solid (49.2 mg, 0.063 mmol, 40.8%): ^1^H NMR (500 MHz, CDCl_3_): δ 7.50–7.43 (m, 3H), 7.41–7.33 (m, 2H), 5.94 (s, 2H), 5.15 (d, *J* = 7.6 Hz, 2H), 4.37 (d, *J* = 4.1 Hz, 2H), 2.57–2.51 (m, 2H), 2.41 (s, 6H), 2.28–2.18 (m, 2H), 2.09 (s, 6H), 1.93–1.84 (m, 2H), 1.42 (s, 18H), 1.39 (s, 6H). ^13^C{^1^H} NMR (126 MHz, CDCl_3_): δ 171.8, 155.2, 153.3, 143.3, 142.7, 134.8, 132.6, 129.0, 128.9, 128.0, 121.4, 79.5, 53.7, 34.6, 32.8, 31.5, 29.0, 28.3, 25.2, 15.5, 14.8, 14.6, 14.0; ^11^B NMR (128 MHz, CDCl_3_): δ 0.03 (s). HRMS (ESI-TOF) *m*/*z* [M + Na]^+^ C_39_H_55_BNaN_4_O_8_S_2_, calcd for 805.3454; found, 805.3463.

BODIPY **7** was synthesized using *N*-Boc-*O*-benzyl-l-serine (182.2 mg, 0.617 mmol) and purified by preparative TLC plates using ethyl acetate/dichloromethane/hexanes (1:1:6) for elution. Compound **7A** was obtained as an orange solid (4.4 mg, 0.007 mmol, 4.8%): ^1^H NMR (500 MHz, CDCl_3_): δ 7.53–7.46 (m, 3H), 7.45–7.41 (m, 1H), 7.33–7.27 (m, 4H), 7.25–7.21 (m, 2H), 5.95 (s, 1H), 5.89 (s, 1H), 5.50 (d, *J* = 8.1 Hz, 1H), 4.54–4.46 (m, 2H), 4.42–4.34 (m, 1H), 4.05 (dd, *J* = 9.2, 2.3 Hz, 1H), 3.75 (dd, *J* = 9.2, 2.6 Hz, 1H), 2.45 (s, 3H), 2.42 (s, 3H), 1.44 (s, 9H), 1.41 (s, 3H), 1.40 (s, 3H). ^13^C{^1^H} NMR (126 MHz, CDCl_3_): δ 170.2, 170.1, 155.4, 155.2, 154.6, 143.3, 143.0, 142.2, 138.0, 135.1, 132.3, 132.2, 129.2, 128.9, 128.3, 128.2, 127.9, 127.5, 127.4, 121.4, 121.3, 79.4, 73.1, 70.7, 55.0, 53.5, 28.4, 14.6, 14.5. ^11^B NMR (128 MHz, CDCl_3_): δ 0.41 (d, *J* = 24.3 Hz). HRMS (ESI-TOF) *m*/*z* [M + Na]^+^ C_34_H_39_BFNaN_3_O_5_, calcd for 622.2865; found, 622.2860. Compound **7B** was obtained as an orange solid (27.0 mg, 0.047 mmol, 30.2%): ^1^H NMR (400 MHz, CDCl_3_): δ 7.49 (bs, 3H), 7.44–7.38 (m, 2H), 7.38–7.29 (m, 3H), 7.29–7.27 (m, 1H), 7.25–7.22 (m, 1H), 5.98 (s, 1H), 5.94 (s, 1H), 4.70 (s, 2H), 4.47 (s, 1H), 4.20–4.07 (m, 2H), 2.33 (s, 3H), 2.27 (s, 3H), 1.37 (s, 3H), 1.36 (s, 3H), 1.22 (s, 9H). ^13^C{^1^H} NMR (126 MHz, CDCl_3_): δ 175.5, 157.2, 156.2, 153.9, 144.1, 142.9, 141.7, 138.4, 135.0, 133.1, 129.5, 129.2, 129.0, 128.5, 128.2, 127.8, 127.5, 127.4, 122.9, 122.5, 78.6, 73.2, 69.3, 61.5, 28.3, 16.4, 15.2, 14.8, 14.6; ^11^B NMR (128 MHz, CDCl_3_): δ 1.99 (s). HRMS (ESI-TOF) *m*/*z* [M + H]^+^ calcd for C_34_H_39_BN_3_O_5_ 580.2983; found, 580.2987. Compound **7C** was obtained as an orange solid (31.0 mg, 0.036 mmol, 23.2%): ^1^H NMR (400 MHz, CDCl_3_): δ 7.51–7.39 (m, 5H), 7.33–7.27 (m, 5H), 7.24–7.17 (m, 5H), 5.83 (s, 2H), 5.45 (d, *J* = 7.9 Hz, 2H), 4.49 (q, *J* = 12.2 Hz, 4H), 4.37 (d, *J* = 6.9 Hz, 2H), 4.03 (d, *J* = 8.6 Hz, 2H), 3.74 (d, *J* = 8.4 Hz, 2H), 2.27 (s, 6H), 1.42 (s, 18H), 1.39 (s, 6H). ^13^C{^1^H} NMR (101 MHz, CDCl_3_): δ 170.2, 155.4, 154.0, 143.0, 137.8, 135.2, 132.7, 129.0, 128.8, 128.3, 127.6, 127.5, 121.4, 79.4, 73.1, 70.5, 54.9, 28.4, 14.6, 14.5; ^11^B NMR (128 MHz, CDCl_3_): δ 0.04 (s). HRMS (ESI-TOF) *m*/*z* [M + Na]^+^ calcd for C_49_H_59_BN_4_NaO_10_, 897.4225; found, 897.4235.

BODIPY **8** was synthesized using *N*-Boc-l-azidolysine (168.0 mg, 0.617 mmol) and purified by preparative TLC plates using ethyl acetate/dichloromethane/hexanes (1:1:6) for elution. Compound **8A** was obtained as an orange solid (12.0 mg, 0.021 mmol, 13.5%): ^1^H NMR (400 MHz, CDCl_3_): δ 7.52–7.45 (m, 3H), 7.42–7.38 (m, 1H), 7.30–7.26 (m, 1H), 5.96 (s, 2H), 5.19 (d, *J* = 7.5 Hz, 1H), 4.27 (dd, *J* = 11.8, 6.7 Hz, 1H), 3.23 (d, *J* = 7.2 Hz, 2H), 2.47 (s, 6H), 2.00–1.86 (m, 1H), 1.75–1.64 (m, 1H), 1.60–1.51 (m, 2H), 1.41 (s, 9H), 1.39 (s, 6H), 1.23–1.16 (m, 1H). ^13^C{^1^H} NMR (126 MHz, CDCl_3_): δ 172.1, 155.3, 154.9, 154.2, 143.5, 143.3, 142.4, 134.9, 132.3, 129.2, 129.0, 128.2, 127.9, 121.4, 121.2, 79.3, 54.1, 51.4, 32.6, 28.7, 28.4, 22.3, 14.7, 14.7, 14.6, 14.6, 14.5, 14.5; ^11^B NMR (128 MHz, CDCl_3_): δ 0.39 (d, *J* = 24.4 Hz). HRMS (ESI-TOF) *m*/*z* [M + H]^+^ calcd for C_30_H_39_BFN_6_O_4_, 577.3110; found, 577.3114. Compound **8B** was obtained as an orange solid (19.2 mg, 0.035 mmol, 22.4%): ^1^H NMR (400 MHz, CDCl_3_): δ 7.48 (bs, 3H), 7.25–7.19 (m, 2H), 5.98 (d, *J* = 7.0 Hz, 2H), 4.12 (d, *J* = 8.6 Hz, 1H), 3.29 (t, *J* = 18.5, 11.8 Hz, 2H), 2.39 (s, 3H), 2.22 (s, 3H), 2.17–2.10 (m, 1H), 2.04–1.86 (m, 2H), 1.81–1.66 (m, 3H), 1.36 (s, 3H), 1.36 (s, 3H), 1.18 (s, 9H). ^13^C{^1^H} NMR (126 MHz, CDCl_3_): δ 177.2, 156.2, 156.0, 154.1, 143.9, 143.2, 141.9, 134.9, 133.1, 132.9, 129.5, 129.2, 129.1, 128.4, 127.4, 122.7, 122.5, 78.4, 60.3, 51.5, 31.8, 28.9, 28.3, 25.7, 16.6, 15.1, 14.7, 14.6; ^11^B NMR (128 MHz, CDCl_3_): δ 1.80 (s). HRMS (ESI-TOF) *m*/*z* [M + H]^+^ calcd for C_30_H_38_BN_6_O_4_, 557.3047; found, 557.3050. Compound **8C** was obtained as an orange solid (23.1 mg, 0.028 mmol, 18.0%): ^1^H NMR (400 MHz, CDCl_3_): δ 7.51–7.44 (m, 3H), 7.42–7.34 (m, 2H), 5.94 (s, 2H), 5.13 (d, *J* = 7.6 Hz, 2H), 4.36–4.25 (m, 2H), 3.33–3.19 (m, 4H), 2.39 (s, 6H), 1.95 (bs, 2H), 1.70–1.55 (m, 6H), 1.41 (s, 18H), 1.40 (s, 6H), 1.28 (m, 8.1 Hz, 2H). ^13^C{^1^H} NMR (126 MHz, CDCl_3_): δ 172.3, 155.3, 153.3, 143.4, 134.9, 132.7, 129.0, 129.0, 128.2, 121.5, 79.5, 54.1, 51.4, 32.6, 29.3, 28.4, 22.5, 14.7, 14.6; ^11^B NMR (128 MHz, CDCl_3_): δ 0.00 (s). HRMS (ESI-TOF) *m*/*z* [M + Na]^+^ calcd for C_41_H_57_BNaN_10_O_8_, 851.4359; found, 851.4353.

BODIPY **9** was synthesized using *N*-α-Boc-*N*-ε-benzyloxycarbonyl-l-lysine (234.7 mg, 0.617 mmol) and purified by preparative TLC plates using ethyl acetate/hexanes (1:2) for elution. Compound **9A** (trace, not isolated). Compound **9B** was obtained as an orange solid (37.0 mg, 0.056 mmol, 36.1%): ^1^H NMR (500 MHz, CD_2_Cl_2_): δ 7.53–7.46 (m, 3H), 7.36–7.22 (m, 7H), 6.02 (d, *J* = 3.6 Hz, 2H), 5.12 (s, 1H), 5.06 (s, 2H), 4.07 (d, *J* = 6.4 Hz, 1H), 3.27–3.18 (m, 2H), 2.36 (s, 3H), 2.20 (s, 3H), 2.07 (s, 1H), 1.95–1.85 (m, 2H), 1.65–1.57 (m, 3H), 1.38 (s, 3H), 1.37 (s, 3H), 1.16 (s, 9H). ^13^C{^1^H} NMR (126 MHz, CD_2_Cl_2_): δ 177.1, 156.3, 156.2, 156.1, 154.3, 144.0, 143.3, 142.0, 137.2, 134.9, 133.1, 132.9, 129.5, 129.1, 128.4, 127.8, 127.8, 127.6, 122.5, 122.5, 78.2, 66.2, 60.3, 40.8, 29.7, 29.4, 28.0, 25.7, 14.9, 14.5, 14.4; ^11^B NMR (128 MHz, CD_2_Cl_2_): δ 1.81 (s). HRMS (ESI-TOF) *m*/*z* [M + Na]^+^ calcd for C_38_H_45_BNaN_4_O_6_, 687.3336; found, 687.3340. Compound **9C** was obtained as an orange solid (48.0 mg, 0.046 mmol, 29.8%): ^1^H NMR (400 MHz, CDCl_3_): δ 7.51–7.44 (m, 3H), 7.41–7.28 (m, 12H), 5.91 (s, 2H), 5.15 (s, 2H), 5.09 (s, 4H), 4.87 (s, 2H), 4.27 (s, 2H), 3.17 (d, *J* = 6.0 Hz, 4H), 2.36 (s, 6H), 1.91 (s, 2H), 1.67 (s, 5H), 1.53 (s, 5H), 1.40 (s, 18H), 1.37 (s, 6H). ^13^C{^1^H} NMR (126 MHz, CDCl_3_): δ 172.4, 156.4, 155.4, 153.3, 143.4, 142.7, 136.6, 134.9, 132.7, 129.0, 128.9, 128.5, 128.2, 128.1, 121.5, 79.5, 66.6, 54.0, 40.9, 32.8, 28.4, 22.5, 14.7, 14.6; ^11^B NMR (128 MHz, CDCl_3_): δ 0.00 (s). HRMS (ESI-TOF) *m*/*z* [M + Na]^+^ calcd for C_57_H_73_BNaN_6_O_12_, 1067.5281; found, 1067.5288.

BODIPY **10** was synthesized using *N*-Boc-l-glutamine (152.0 mg, 0.616 mmol) and purified by preparative TLC using dichloromethane/methanol/ammonium hydroxide in a 90:9.5:0.5 ratio for elution. Compound **10A** was obtained as an orange solid (7.3 mg, 0.013 mmol, 8.6%). ^1^H NMR (400 MHz, CDCl_3_): δ 7.52–7.45 (m, 3H), 7.40–7.36 (m, 1H), 7.30–7.27 (m, 1H), 6.41 (s, 1H), 5.96 (s, 2H), 5.37–5.28 (m, 1H), 4.27 (d, *J* = 5.6 Hz, 1H), 2.48 (s, 3H), 2.47 (s, 3H), 2.36–2.25 (m, 2H), 1.91–1.80 (m, 1H), 1.41 (s, 9H), 1.39 (s, 6H). ^13^C{^1^H} NMR (126 MHz, CDCl_3_): δ 174.7, 171.6, 156.1, 154.8, 154.6, 143.4, 142.4, 134.9, 132.3, 129.2, 129.0, 129.0, 128.1, 127.9, 121.4, 121.3, 79.8, 53.7, 32.2, 30.1, 28.3, 14.7, 14.5; ^11^B NMR (128 MHz, CDCl_3_): δ 0.39 (d, *J* = 22.9 Hz). HRMS (ESI-TOF) *m*/*z* [M + Na]^+^ calcd for C_29_H_36_BFNaN_4_O_5_ 573.2660; found, 573.2675. Compound **10B** was obtained as an orange solid (0.037 mmol, 24.3%). ^1^H NMR (500 MHz, CDCl_3_): δ 7.54–7.43 (m, 3H), 7.25–7.15 (m, 2H), 6.00 (d, *J* = 9.5 Hz, 2H), 4.20 (dd, *J* = 9.4, 2.4 Hz, 1H), 2.85–2.77 (m, 2H), 2.51–2.42 (m, 1H), 2.40 (s, 3H), 2.29–2.24 (m, 1H), 2.22 (s, 3H), 1.38 (s, 3H), 1.37 (s, 3H), 1.19 (s, 9H). ^13^C{^1^H} NMR (126 MHz, CDCl_3_): δ 176.7, 174.9, 156.7, 156.1, 154.0, 144.1, 143.3, 141.9, 134.9, 133.1, 133.0, 129.6, 129.2, 129.1, 128.4, 127.4, 122.9, 122.6, 78.9, 59.3, 33.7, 28.2, 27.0, 14.7, 14.6; ^11^B NMR (128 MHz, CD_3_OD): δ 1.87 (s). HRMS (ESI-TOF) *m*/*z* [M + Na]^+^ calcd for C_29_H_35_BNaN_4_O_5_ 553.2598; found, 553.2607. Compound **10C** was obtained as an orange solid (30.4 mg, 0.039 mmol, 25.4%). ^1^H NMR (400 MHz, CDCl_3_): δ 7.52–7.43 (m, 3H), 7.40–7.32 (m, 2H), 6.69 (bs, 2H), 5.95 (s, 2H), 5.84 (bs, 2H), 5.39 (d, *J* = 7.8 Hz, 2H), 4.25 (bs, 2H), 2.38 (s, 6H), 2.35–2.24 (m, 4H), 1.95–1.79 (m, 2H), 1.40 (s, 18H), 1.36 (s, 6H). ^13^C{^1^H} NMR (126 MHz, CDCl_3_): δ 175.1, 171.7, 155.9, 153.5, 143.4, 142.7, 134.8, 132.6, 129.0, 128.9, 128.0, 121.6, 79.8, 69.5, 53.7, 32.3, 28.3, 14.8, 14.6; ^11^B NMR (128 MHz, CDCl_3_): δ 0.01 (s). HRMS (ESI-TOF) *m*/*z* [M + Na]^+^ calcd for C_39_H_53_BNaN_6_O_10_ 799.3815; found, 799.3817.

BODIPY **11** was synthesized using *N*-Boc-Gly-Gly-Gly (234.7 mg, 0.617 mmol) and purified by preparative TLC plates using ethyl acetate/hexanes (1:2) for elution. Compound **11A** (trace, not isolated). Compound **11B** was obtained as an orange solid (23.0 mg, 0.040 mmol, 26.0%): ^1^H NMR (400 MHz, CDCl_3_): δ 7.67–7.57 (m, 2H), 7.55–7.47 (m, 2H), 7.32–7.27 (m, 1H), 6.85 (s, 1H), 6.06 (s, 2H), 5.04 (s, 1H), 4.24 (s, 2H), 3.78 (d, *J* = 5.5 Hz, 2H), 3.36 (d, *J* = 3.7 Hz, 2H), 2.27 (s, 6H), 1.44 (s, 9H), 1.42 (s, 6H). ^13^C{^1^H} NMR (126 MHz, CDCl_3_): δ 172.6, 168.8, 155.8, 145.6, 143.5, 134.1, 132.7, 130.0, 129.4, 129.1, 128.3, 127.3, 123.5, 80.3, 50.2, 41.1, 29.7, 28.3, 14.8, 14.7; ^11^B NMR (128 MHz, CDCl3): δ 1.95 (s). HRMS (ESI-TOF) *m*/*z* [M + H]^+^ calcd for C_30_H_37_BN_5_O_6_, 574.2837; found, 574.2828. Compound **11C** was obtained as an orange solid (26.0 mg, 0.030 mmol, 19.5%):^1^H NMR (400 MHz, CDCl3): δ 7.51–7.42 (m, 3H), 7.32 (d, *J* = 7.3 Hz, 2H), 7.25–7.20 (m, 1H), 7.04 (s, 2H), 5.92 (s, 2H), 5.57 (s, 2H), 3.92 (dd, *J* = 16.6, 5.0 Hz, 8H), 3.76 (d, *J* = 3.5 Hz, 4H), 2.50 (s, 2H), 2.34 (s, 6H), 2.15 (s, 1H), 1.39 (s, 18H), 1.36 (s, 6H). ^13^C{^1^H} NMR (126 MHz, CDCl_3_): δ 170.3, 169.2, 169.1, 156.3, 153.7, 143.5, 142.8, 134.8, 132.6, 129.2, 129.0, 128.0, 121.6, 80.3, 69.5, 54.0, 44.1, 42.6, 42.4, 31.7, 31.6, 30.9, 29.7, 29.3, 28.3, 22.6, 14.6, 14.5, 14.1; ^11^B NMR (128 MHz, CDCl_3_): δ −0.23 (s). HRMS (ESI-TOF) *m*/*z* [M + Na]^+^ calcd for C_41_H_55_BNaN_8_O_12_, 885.3930; found, 885.3935.

#### Removal of Side Chain-Protecting Groups of α-Amino Acid-BODIPY Derivatives

To a 10 mL reaction flask were added 2 mL of methanol and 10% Pd/C (10 mg). After purging the solution with hydrogen for 10 min, the amino acid–BODIPY compounds **2–4**, and **7** (10 mg) in a mixture of dichloromethane/methanol (1/1) (1 mL) were added, and the reaction mixture was stirred until the disappearance of the starting material. When the starting material disappeared, the Pd/C was filtered. The mixture was then concentrated under vacuum, giving the corresponding products in quantitative yields.

BODIPY **2b** was obtained as an orange solid (8.4 mg, 0.016 mmol, 98.9%): ^1^H NMR (400 MHz, CDCl_3_): δ 7.54–7.47 (m, 3H), 7.29–7.27 (m, 1H), 7.25–7.19 (m, 1H), 6.03 (s, 2H), 4.74 (bs, 1H), 3.18–3.07 (m, 2H), 2.32 (s, 3H), 2.28 (s, 3H), 1.40 (s, 3H), 1.38 (s, 3H), 1.21 (s, 9H). ^13^C{^1^H} NMR (126 MHz, CDCl_3_): δ 175.5, 155.5, 154.4, 144.2, 143.8, 142.1, 134.7, 133.2, 133.0, 129.6, 129.3, 129.2, 128.3, 127.3, 122.9, 122.8, 80.0, 56.7, 38.6, 28.2, 16.3, 15.2, 14.7; ^11^B NMR (128 MHz, CDCl_3_): δ 1.78 (s). HRMS (ESI-TOF) *m*/*z* [M + H]^+^ calcd for C_28_H_33_BN_3_O_6_, 518.2462; found, 518.2459. Compound **2c** was obtained as an orange solid (7.9 mg, 0.011 mmol, 98.0%): ^1^H NMR (500 MHz, CDCl_3_): δ 7.55–7.44 (m, 3H), 7.36 (d, *J* = 6.1 Hz, 2H), 5.97 (s, 1H), 5.42 (d, *J* = 5.8 Hz, 2H), 4.64 (bs, 2H), 3.26 (d, *J* = 13.3 Hz, 2H), 2.50 (dd, *J* = 14.7, 10.4 Hz, 2H), 2.38 (s, 6H), 1.41 (s, 18H), 1.40 (s, 6H). ^13^C{^1^H} NMR (126 MHz, CDCl_3_): δ 176.7, 175.2, 170.4, 155.2, 154.2, 143.7, 142.7, 134.8, 132.6, 129.2, 129.1, 128.0, 121.9, 80.0, 51.7, 38.0, 28.3, 20.5, 14.7, 14.6; ^11^B NMR (128 MHz, CDCl_3_): δ 0.01 (s). HRMS (ESI-TOF) *m*/*z* [M + Na]^+^ calcd for C_37_H_47_BNaN_4_O_12_, 773.3182; found, 773.3188.

BODIPY **3b** was obtained as an orange solid (6.1 mg, 0.011 mmol, 90.0%): ^1^H NMR (400 MHz, CDCl_3_): δ 7.50 (s, 3H), 7.39–7.34 (m, 1H), 7.26–7.21 (m, 1H), 6.03 (s, 1H), 6.00 (s, 1H), 4.21–4.13 (m, 1H), 3.44–3.29 (m, 2H), 2.40 (s, 3H), 2.24 (s, 3H), 2.16–1.98 (m, 4H), 1.39 (s, 3H), 1.38 (s, 3H), 1.19 (s, 9H). ^13^C{^1^H} NMR (126 MHz, CDCl_3_): δ 177.2, 156.7, 156.5, 153.9, 144.2, 143.1, 141.8, 134.9, 133.0, 129.5, 129.1, 128.4, 127.5, 123.1, 122.6, 79.1, 59.9, 40.8, 29.3, 28.3, 27.5, 16.8, 15.2, 14.8, 14.6; ^11^B NMR (128 MHz, CDCl_3_): δ 1.84 (s). HRMS (ESI-TOF) *m*/*z* [M + H]^+^ calcd for C_30_H_40_BN_6_O_4_, 559.3204; found, 559.3222. Compound **3c** was obtained as an orange solid (6.0 mg, 0.007 mmol, 93%): ^1^H NMR (400 MHz, CD_3_OD): δ 7.59–7.51 (m, 3H), 7.44–7.39 (m, 2H), 6.00 (s, 2H), 4.12 (bs, 2H), 3.24–3.16 (m, 4H), 2.46 (s, 6H), 1.90 (bs, *J* = 8.0 Hz, 2H), 1.72–1.59 (m, 8H), 1.44 (s, 18H), 1.39 (s, 6H). ^13^C{^1^H} NMR (126 MHz, CD_3_OD): δ 172.8, 157.2, 156.7, 153.6, 143.1, 135.2, 132.7, 128.9, 128.8, 128.0, 120.8, 79.2, 69.2, 54.7, 54.3, 40.6, 30.7, 28.6, 28.1, 27.4, 25.2, 13.9, 13.2; ^11^B NMR (128 MHz, CD_3_OD): δ 0.08 (s). HRMS (ESI-TOF) *m*/*z* [M + H]^+^ calcd for C_41_H_62_BN_10_O_8_, 833.4847; found, 833.4842.

BODIPY **4b** was obtained as an orange solid (7.7 mg, 0.014 mmol, 96.1%): ^1^H NMR (400 MHz, CDCl_3_): δ 7.73 (s, 1H), 7.54–7.45 (m, 3H), 7.30–7.27 (m, 1H), 7.23 (bs, 1H), 7.05 (s, 1H), 6.02 (d, *J* = 13.6 Hz, 2H), 4.81 (bs, 1H), 4.36 (d, *J* = 6.4 Hz, 1H), 3.54–3.43 (m, 1H), 3.36–3.25 (m, 1H), 2.42 (s, 3H), 2.18 (s, 3H), 1.39 (s, 6H), 1.22 (s, 9H). ^13^C{^1^H} NMR (126 MHz, CDCl_3_): δ 177.6, 156.5, 155.7, 154.2, 144.3, 143.7, 142.1, 134.8, 133.0, 129.6, 129.3, 129.2, 129.0, 128.3, 127.4, 124.6, 122.9, 122.7, 79.3, 60.8, 28.3, 27.9, 16.5, 15.2, 14.7; ^11^B NMR (128 MHz, CDCl_3_): δ 1.92 (s). HRMS (ESI-TOF) *m*/*z* [M + H]^+^ calcd for C_30_H_35_BN_5_O_4_ 540.2782; found, 540.2795. Compound **4c** was obtained as an orange solid (7.3 mg, 0.009 mmol, 97.3%): ^1^H NMR (400 MHz, CDCl_3_): δ 7.81 (s, 2H), 7.50–7.43 (m, 3H), 7.37–7.33 (m, 2H), 6.89 (s, 2H), 5.91 (s, 2H), 5.43 (d, *J* = 7.1 Hz, 2H), 5.11 (bs, 4H), 4.50 (bs, 2H), 3.26 (dd, *J* = 14.7, 4.5 Hz, 2H), 2.97 (dd, *J* = 13.9, 6.3 Hz, 2H), 2.18 (s, 6H), 1.38 (s, 6H), 1.35 (s, 18H). ^13^C{^1^H} NMR (126 MHz, CDCl_3_): δ 171.6, 155.4, 143.4, 134.9, 134.6, 132.6, 129.1, 129.0, 128.1, 121.6, 79.7, 54.4, 30.3, 29.3, 28.3, 14.6, 14.5; ^11^B NMR (128 MHz, CDCl_3_): δ 0.02 (s). HRMS (ESI-TOF) *m*/*z* [M + H]^+^ calcd for C_41_H_52_BN_8_O_8_ 795.4003; found, 795.3995.

BODIPY **7b** was obtained as an orange solid (8.1 mg, 0.016 mmol, 95.5%): ^1^H NMR (500 MHz, CD_2_Cl_2_): δ 7.61–7.45 (m, 3H), 7.34–7.18 (m, 2H), 6.06 (s, 2H), 5.01 (s, 1H), 4.54–4.39 (m, 1H), 4.25–4.01 (m, 2H), 2.29 (s, 3H), 2.27 (s, 3H), 1.41 (s, 6H), 1.21 (s, 9H). ^13^C{^1^H} NMR (126 MHz, CD_2_Cl_2_): δ 173.7, 158.2, 155.9, 154.5, 144.3, 143.8, 142.3, 134.8, 133.3, 133.1, 129.6, 129.3, 129.2, 128.5, 127.6, 122.8, 122.7, 100.1, 79.6, 64.7, 64.5, 28.1, 16.3, 15.2, 14.5; ^11^B NMR (128 MHz, CD_2_Cl_2_): δ 1.64 (s). HRMS (ESI-TOF) *m*/*z* [M + H]^+^ calcd for C_27_H_33_BN_3_O_5_, 490.2513; found, 490.2532. Compound **7c** was obtained as an orange solid (8.2 mg, 0.011 mmol, 94%): ^1^H NMR (400 MHz, CDCl_3_): δ 7.48 (m, 3H), 7.40–7.31 (m, 2H), 5.95 (s, 2H), 5.50 (d, *J* = 5.5 Hz, 2H), 4.32 (bs, 2H), 4.02 (bs, 4H), 2.42 (s, 6H), 1.42 (s, 9H), 1.40 (s, 6H). ^13^C{^1^H} NMR (126 MHz, CDCl_3_): δ 170.3, 155.9, 153.9, 143.4, 142.6, 134.9, 132.6, 129.1, 129.0, 128.1, 121.7, 80.0, 63.9, 56.8, 28.3, 14.6, 14.5; ^11^B NMR (128 MHz, CDCl_3_): δ 0.08 (s). HRMS (ESI-TOF) *m*/*z* [M + H]^+^ calcd for C_35_H_47_BNaN_4_O_10_, 717.3284; found, 717.3317.

## Supporting Information Available

^1^H, ^13^C, and ^11^B NMR spectra, cytotoxicity, fluorescence imaging, and frontier orbitals of BODIPYs (PDF)
