# Supplementary material for: Syntheses and Investigations of Conformationally Restricted, Linker-Free α-Amino Acid–BODIPYs via Boron Functionalization
Source: J Org Chem. 2021 Nov 22;86(24):18030–41. doi: 10.1021/acs.joc.1c02328 (PMC8689652; doi:10.1021/acs.joc.1c02328)
Supplement: Supplementary file 1 — jo1c02328_si_001.pdf [file jo1c02328_si_001.pdf]

Supporting Information

Syntheses and Investigations of Conformationally Restricted, Linker-free α-amino acid-BODIPYs via Boron Functionalization

Maodie Wang‡[a], Guanyu Zhang‡[a], Petia Bobadova-Parvanova[b], Kevin M. Smith[a], and M. Graça H. Vicente*[a]

[a]Department of Chemistry, Louisiana State University, Baton Rouge, LA 70803, United States

[b]Department of Chemistry and Fermentation Sciences, Appalachian State University, Boone, NC 28607, United States

Content

NMR Spectra S2

Computational Modeling S53

Frontier Orbitals S55

Cytotoxicity S57

NMR Spectra
Compound 2A

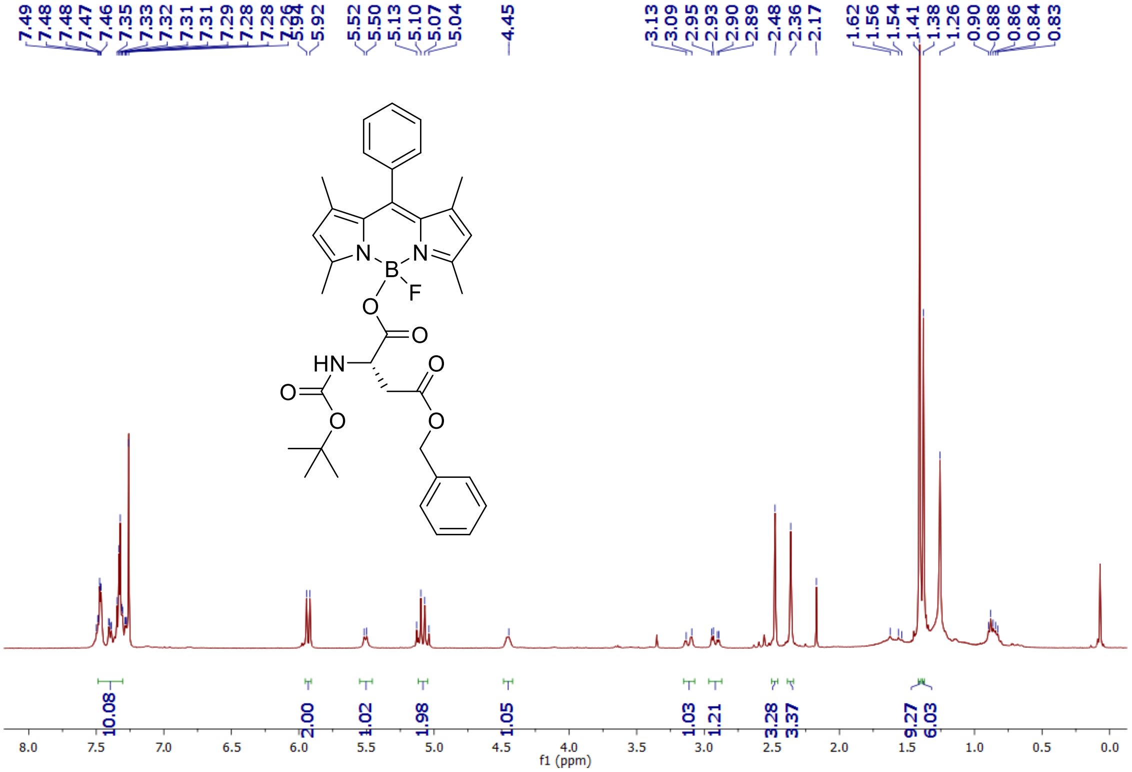

Figure S1. ^{1}H NMR (400 MHz, $CDCl_3$) spectrum of compound **2A**.

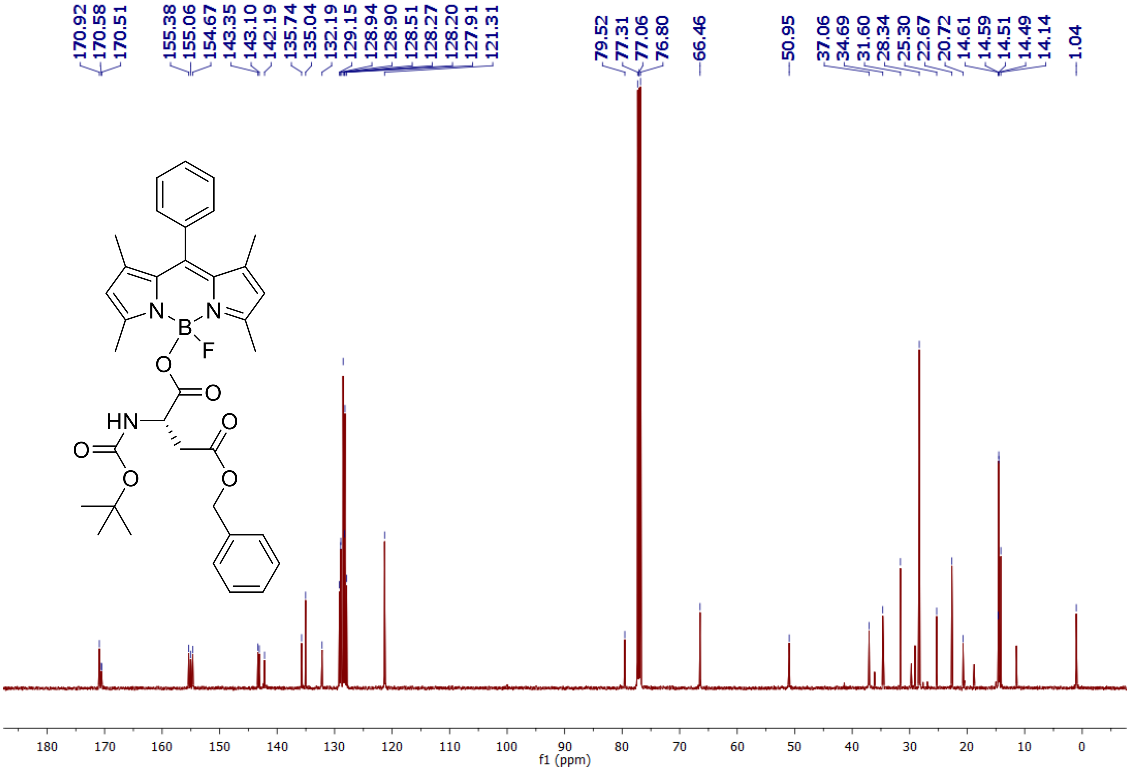

Figure S2. $^{13}C\{^{1}H\}$ NMR (100 MHz, $CDCl_3$) spectrum of compound **2A**.

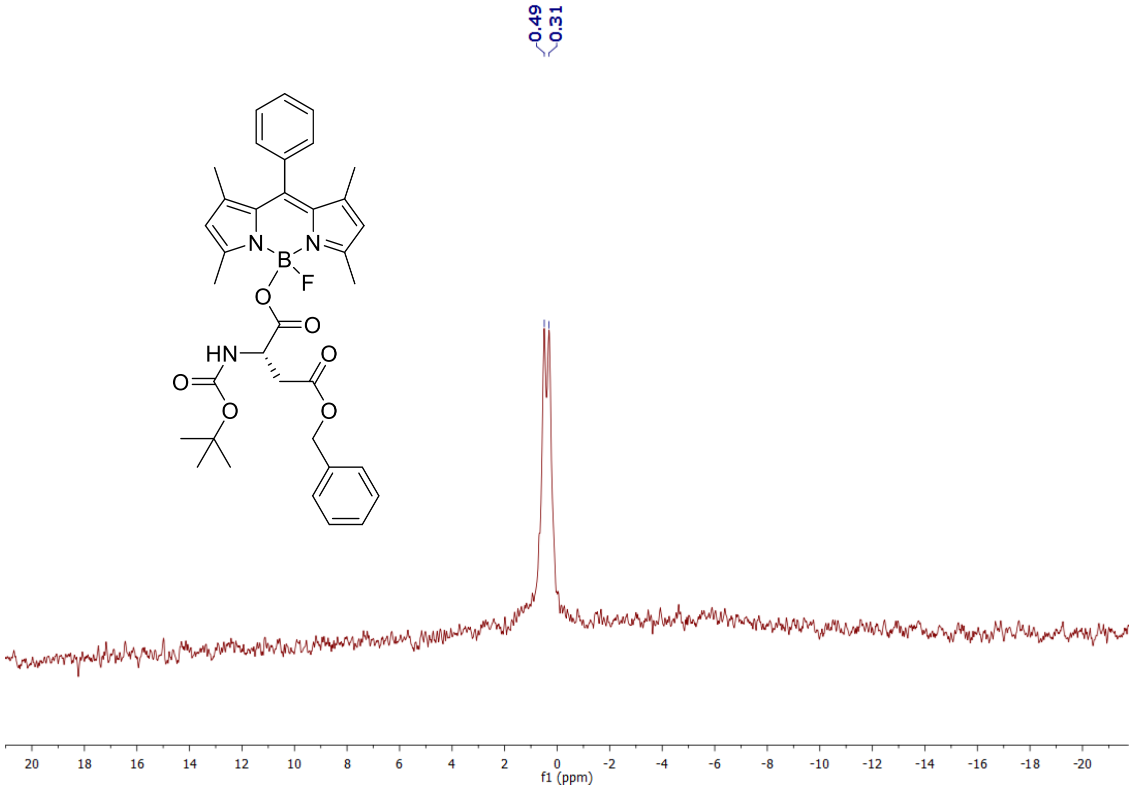

Figure S3. ^{11}B NMR (128 MHz, $CDCl_3$) spectrum of compound **2A**.

Compound 2B

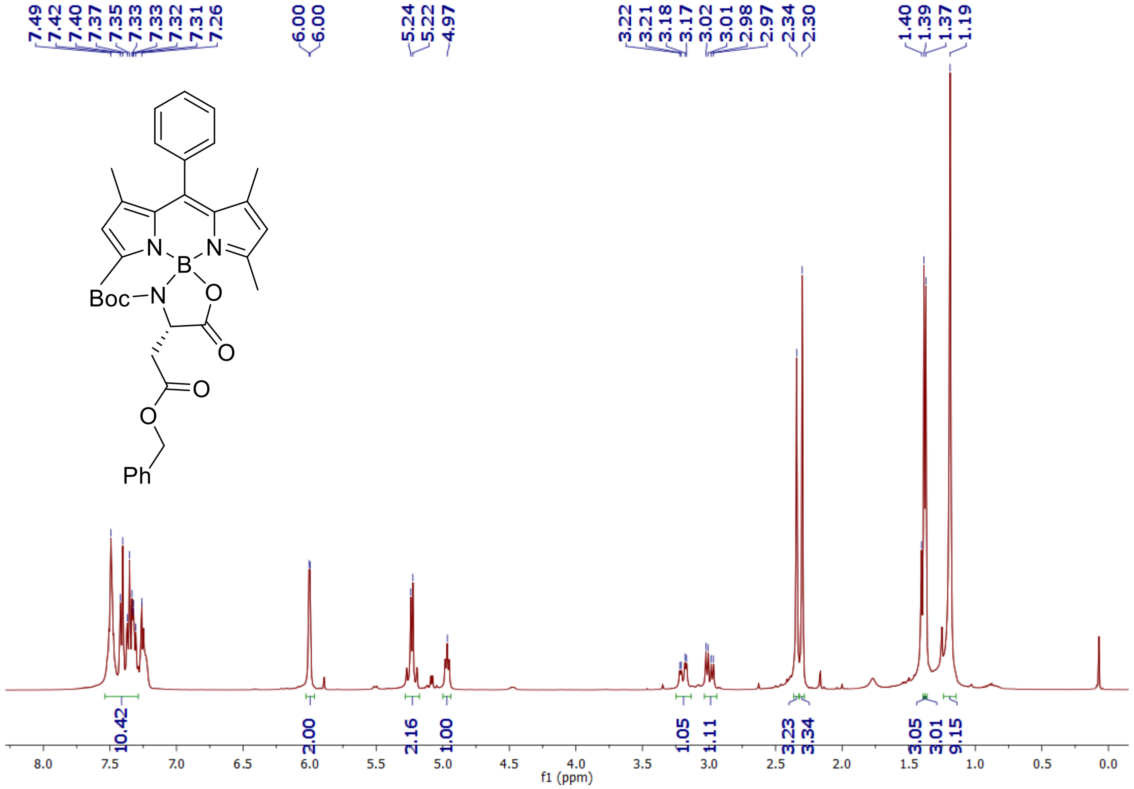

Figure S4. ^{1}H NMR (400 MHz, $CDCl_3$) spectrum of compound **2B**.

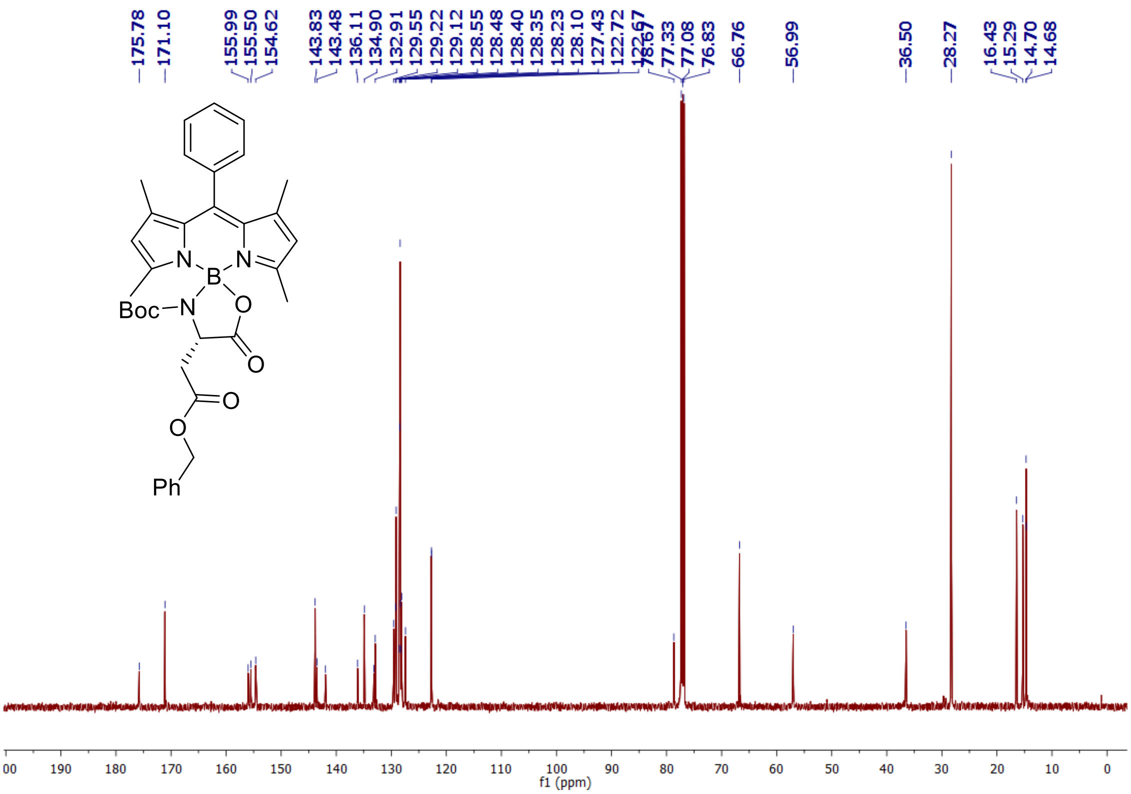

Figure S5. $^{13}C\{^{1}H\}$ NMR (100 MHz, $CDCl_3$) spectrum of compound **2B**.

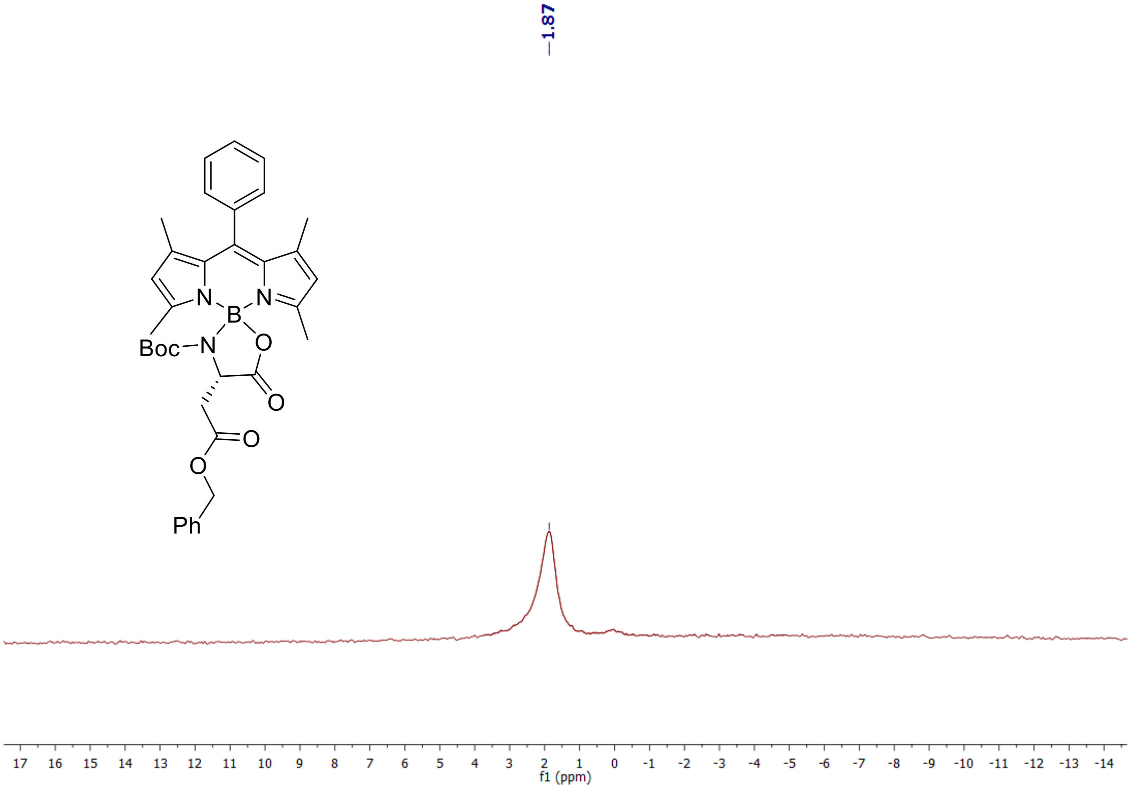

Figure S6. ^{11}B NMR (128 MHz, $CDCl_3$) spectrum of compound **2B**.

Compound 2C

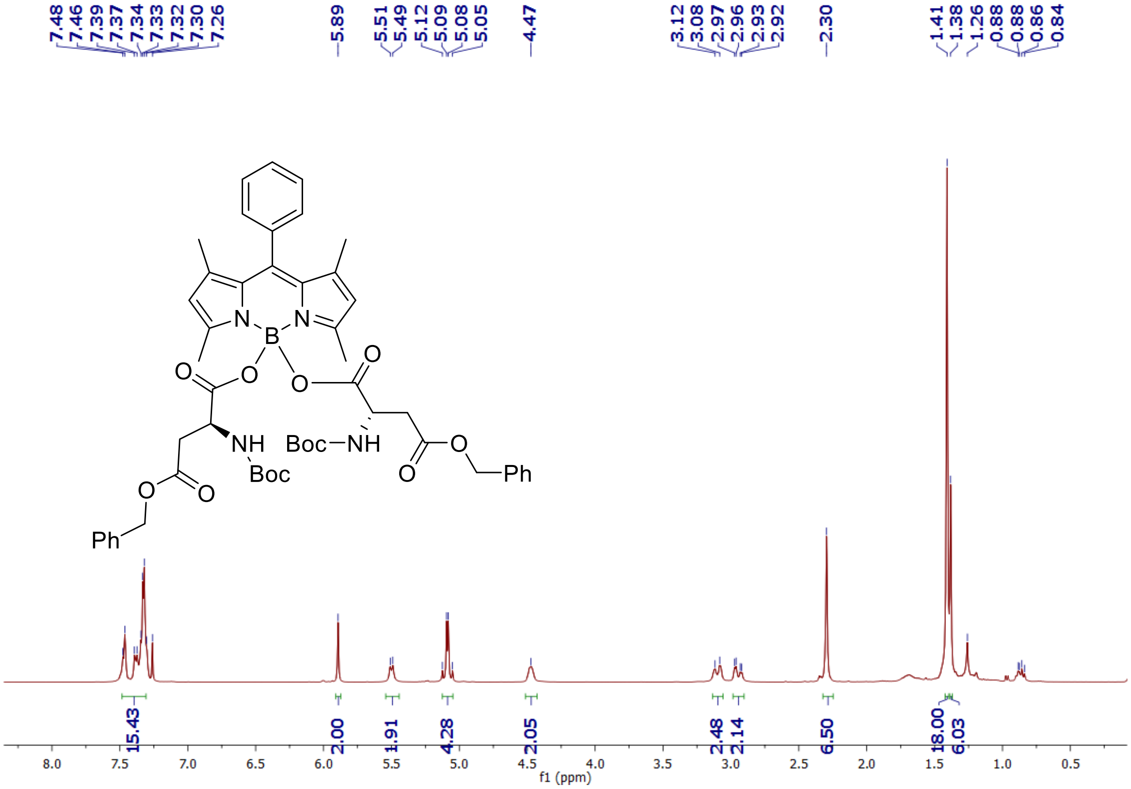

Figure S7. ^{1}H NMR (400 MHz, $CDCl_3$) spectrum of compound **2C**.

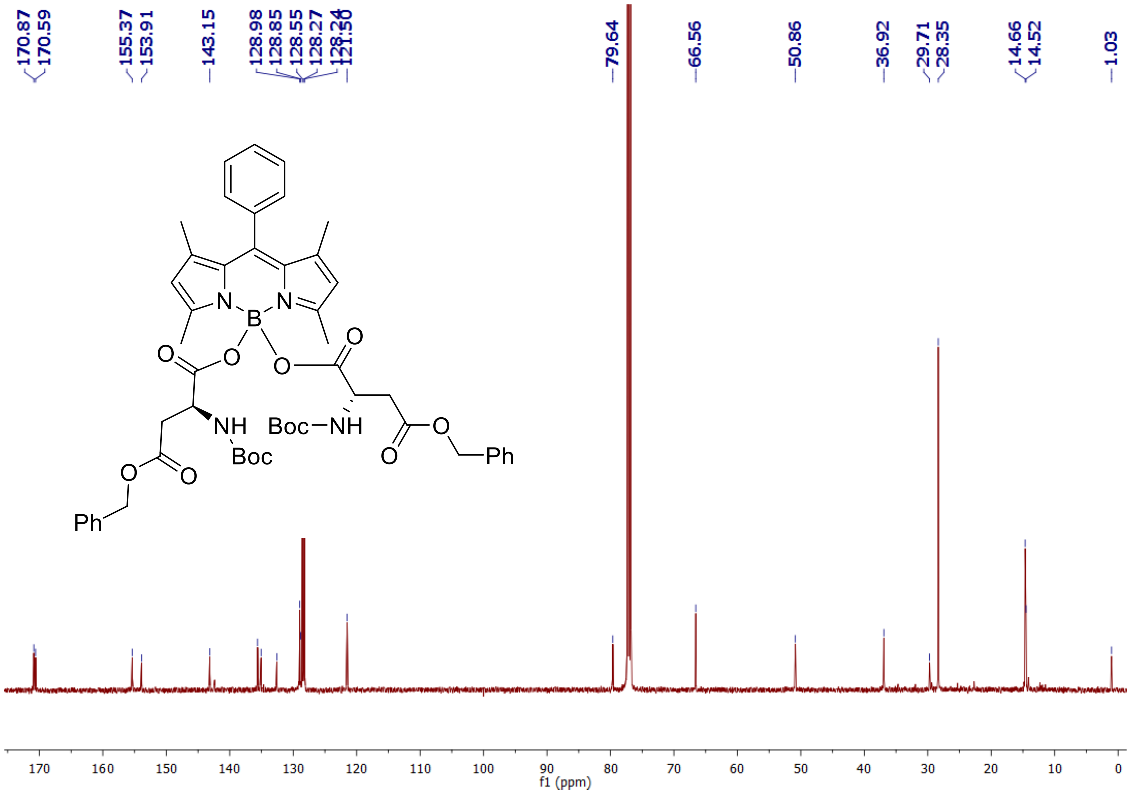

Figure S8. $^{13}C\{^1H\}$ NMR (100 MHz, $CDCl_3$) spectrum of compound **2C**.

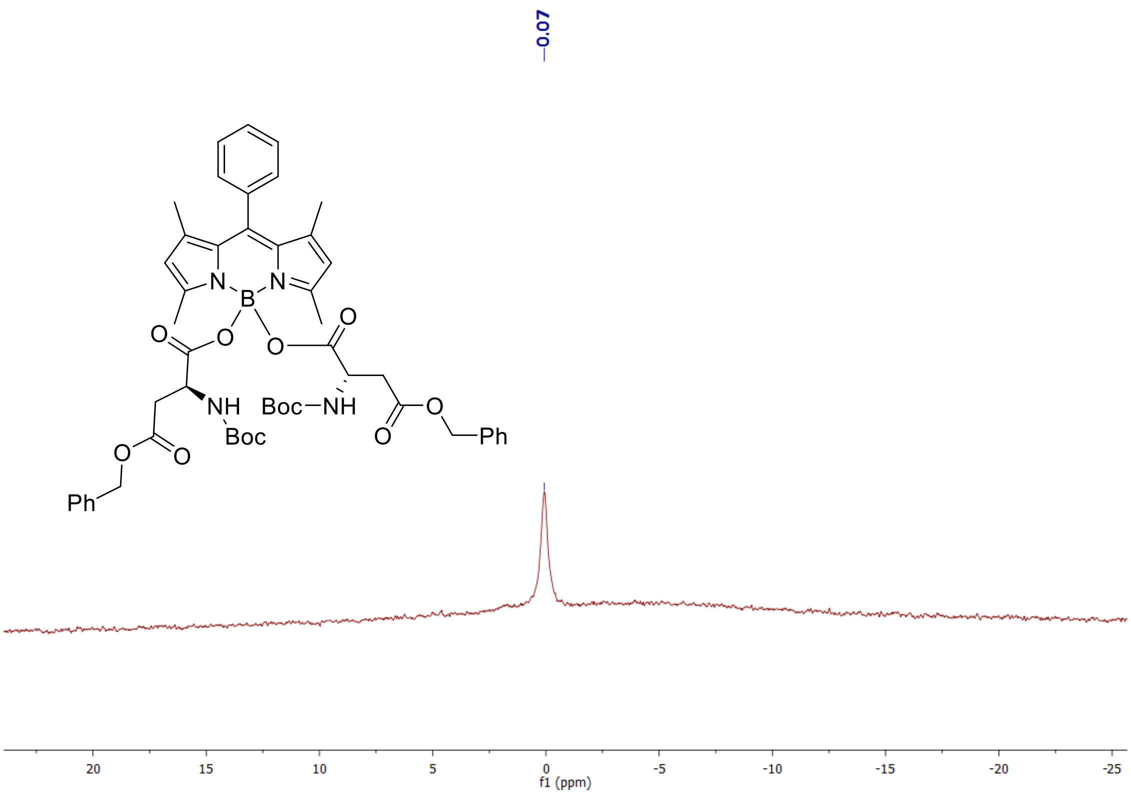

Figure S9. ^{11}B NMR (128 MHz, $CDCl_3$) spectrum of compound **2C**.

Compound 3A

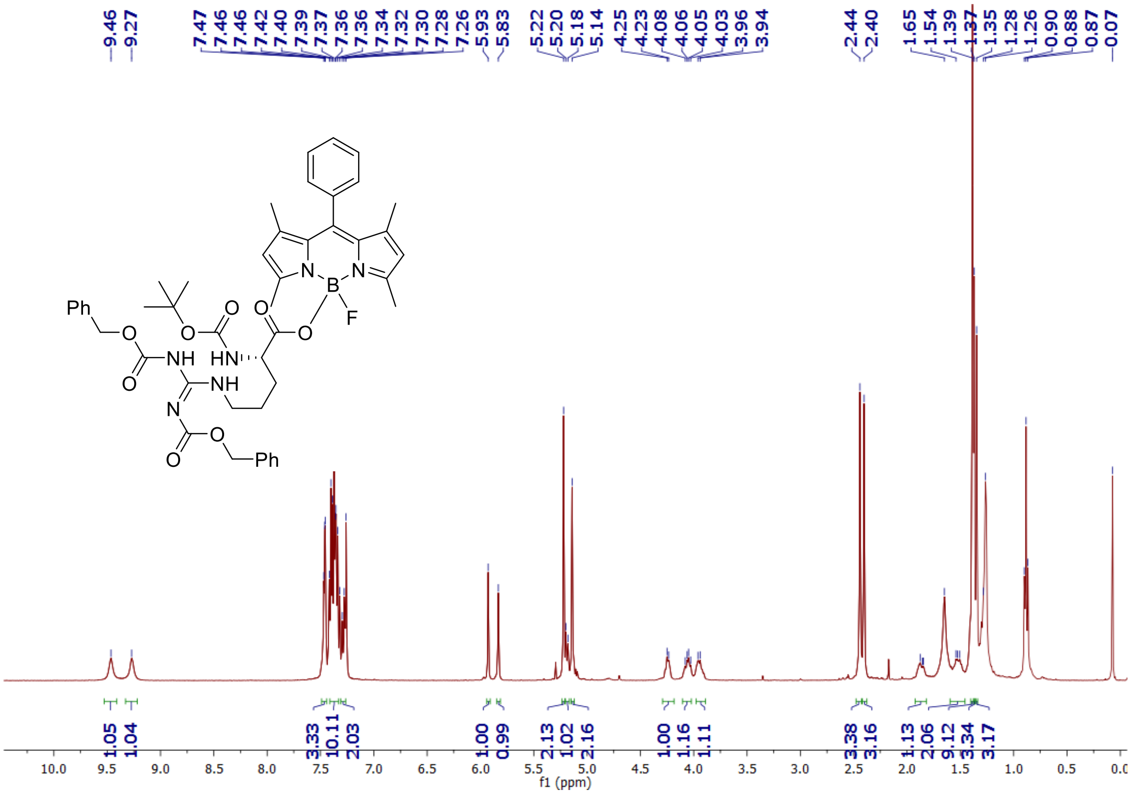

Figure S10. ^{1}H NMR (400 MHz, $CDCl_3$) spectrum of compound **3A**.

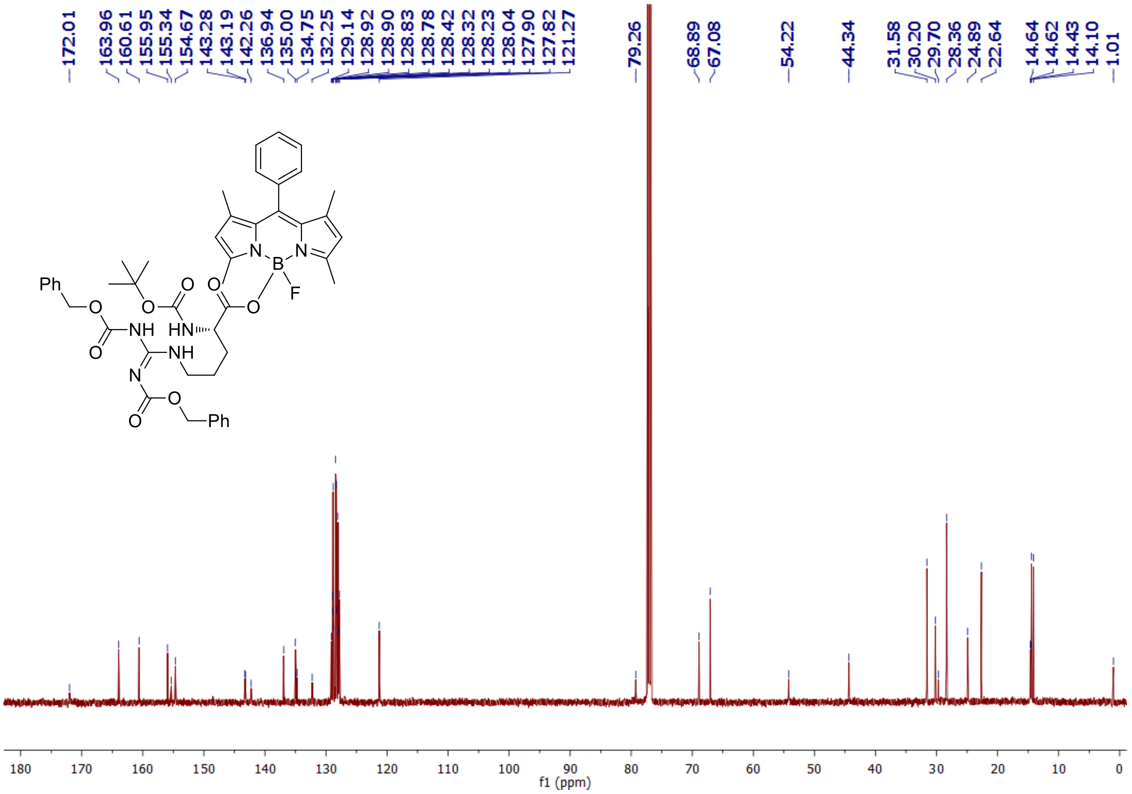

Figure S11. $^{13}C\{^{1}H\}$ NMR (100 MHz, $CDCl_3$) spectrum of compound **3A**.

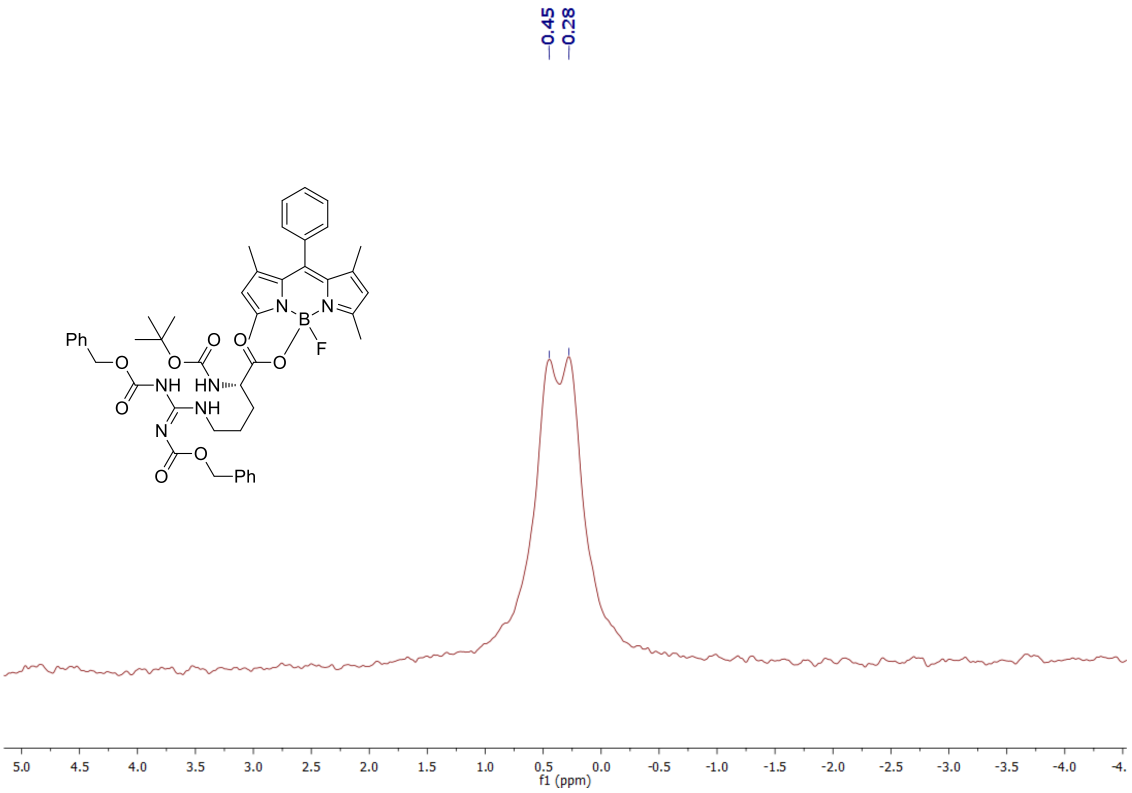

Figure S12. ^{11}B NMR (128 MHz, $CDCl_3$) spectrum of compound **3A**.

Compound 3B

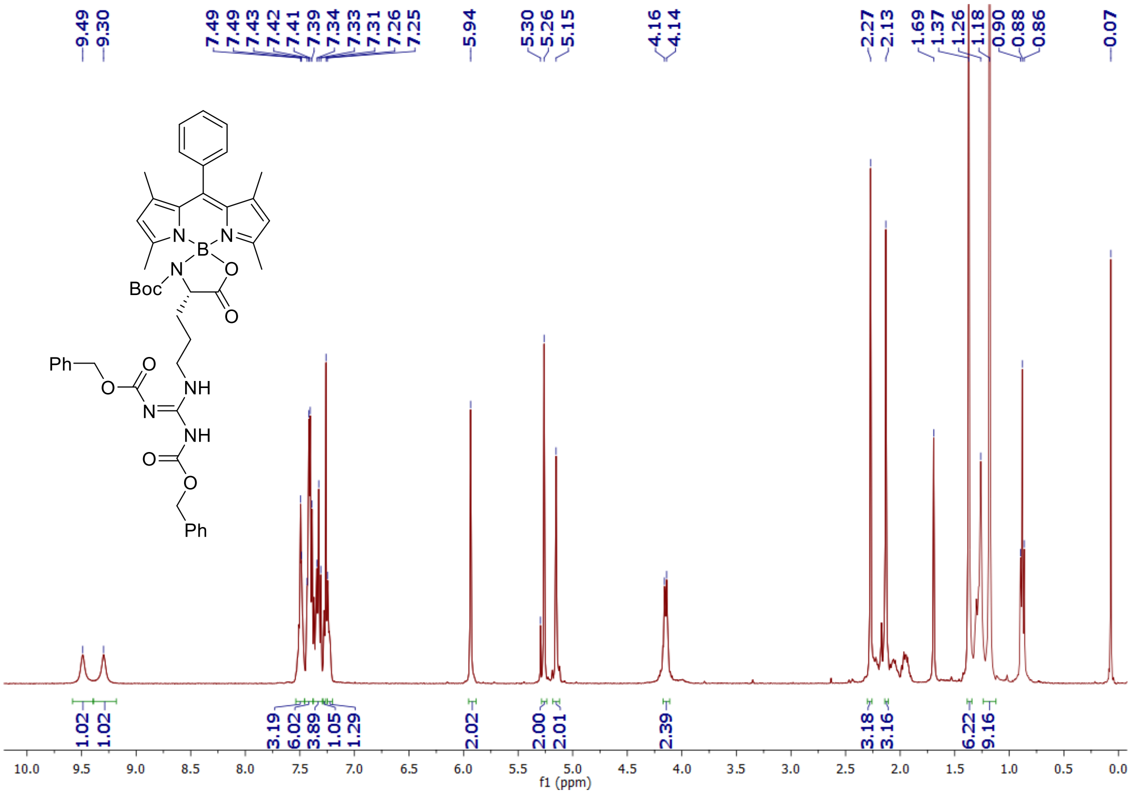

Figure S13. ^{1}H NMR (400 MHz, $CDCl_3$) spectrum of compound **3B**.

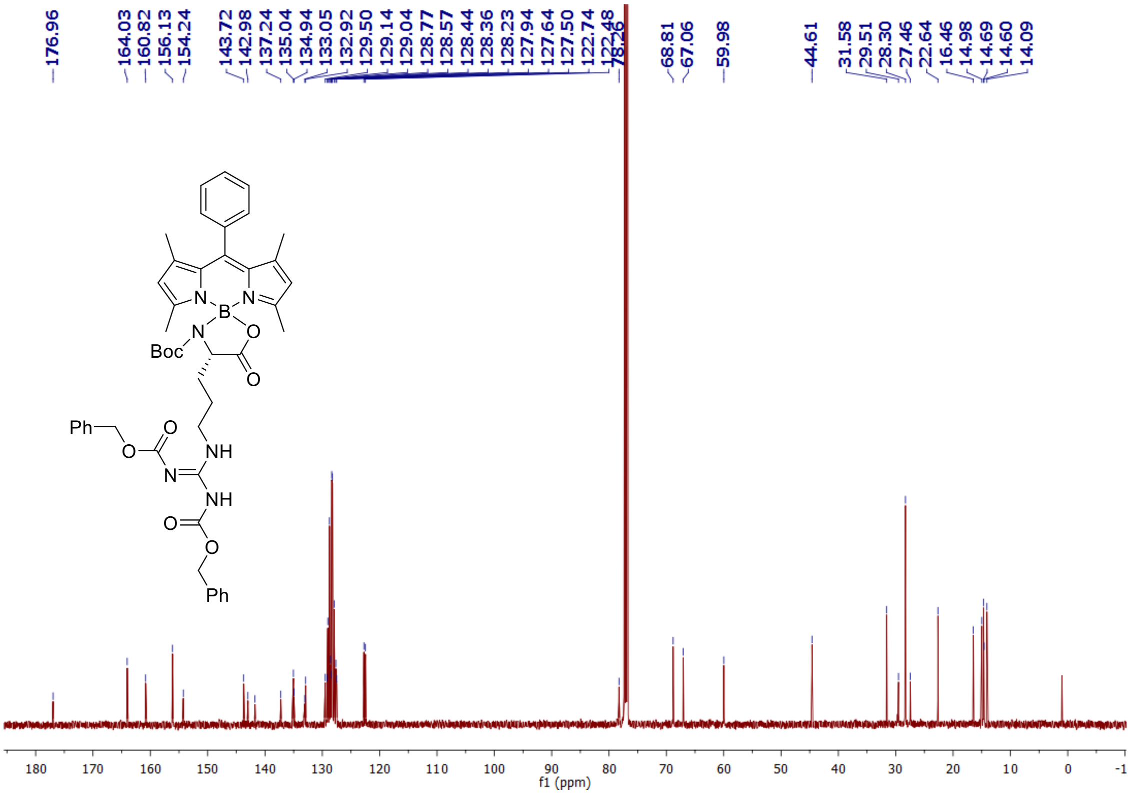

Figure S14. $^{13}C\{^{1}H\}$ NMR (100 MHz, $CDCl_3$) spectrum of compound **3B**.

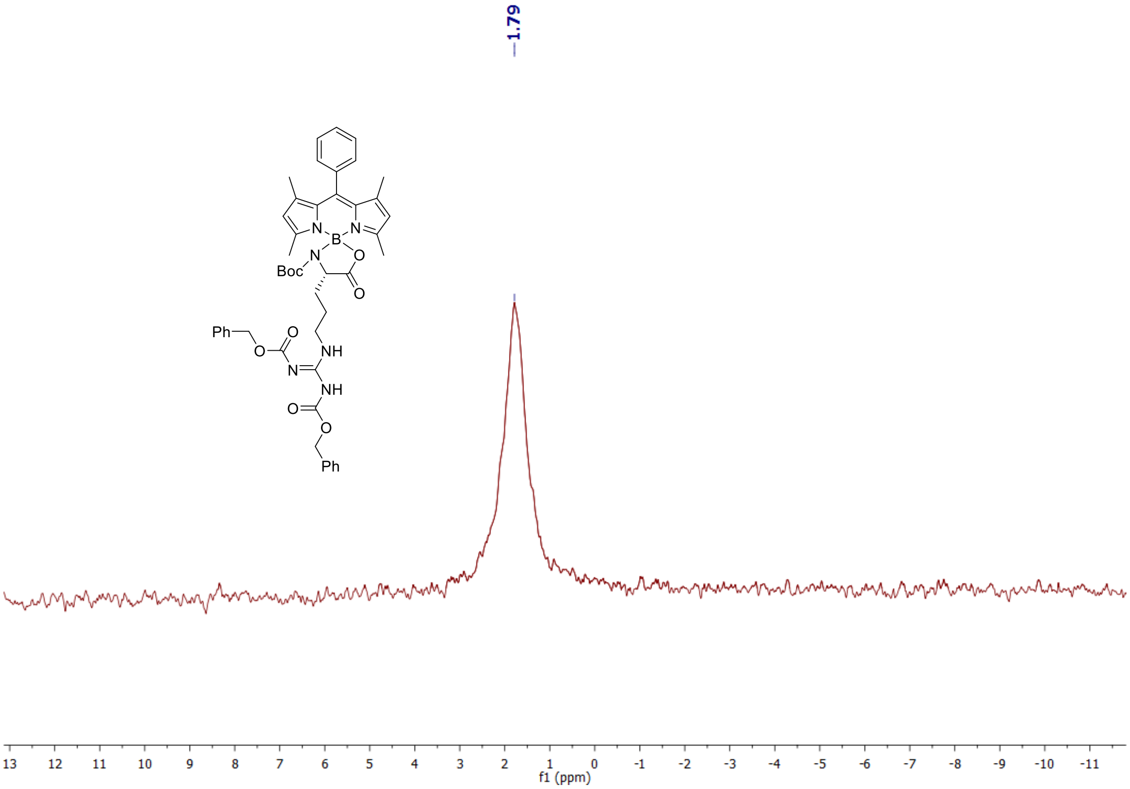

Figure S15. ^{11}B NMR (128 MHz, $CDCl_3$) spectrum of compound **3B**.

Compound 3C

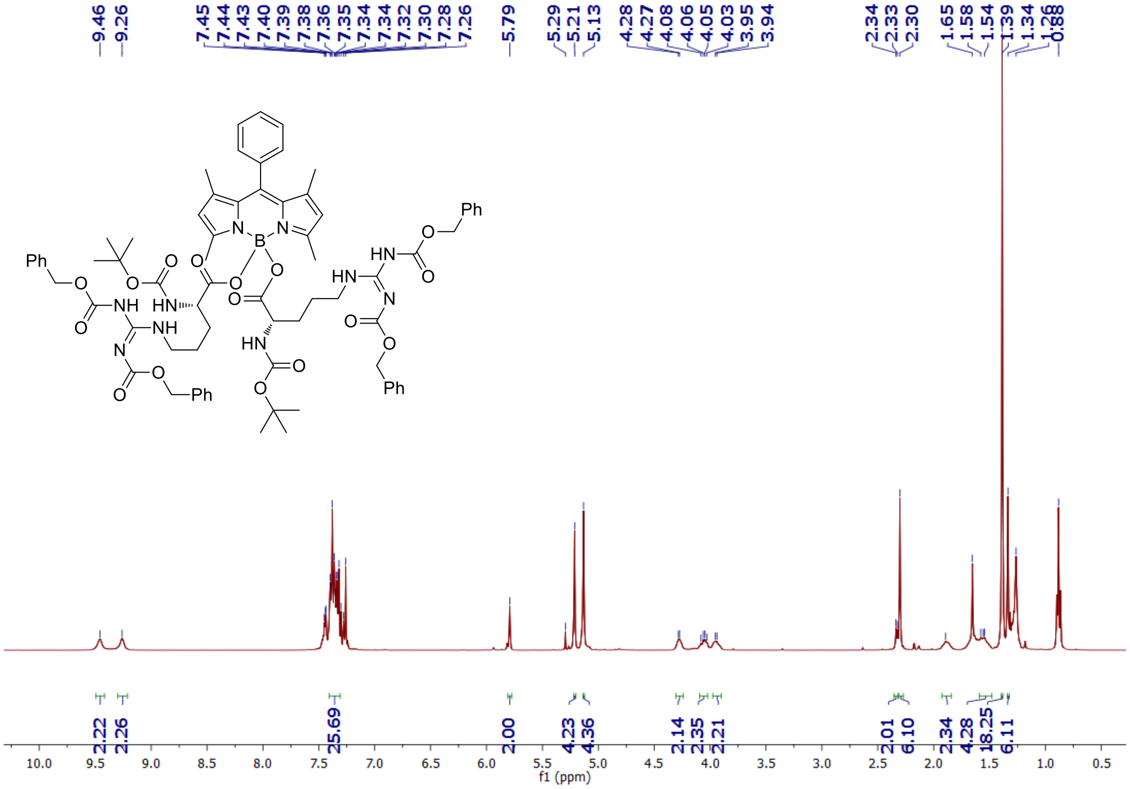

Figure S16. ^{1}H NMR (400 MHz, $CDCl_3$) spectrum of compound **3C**.

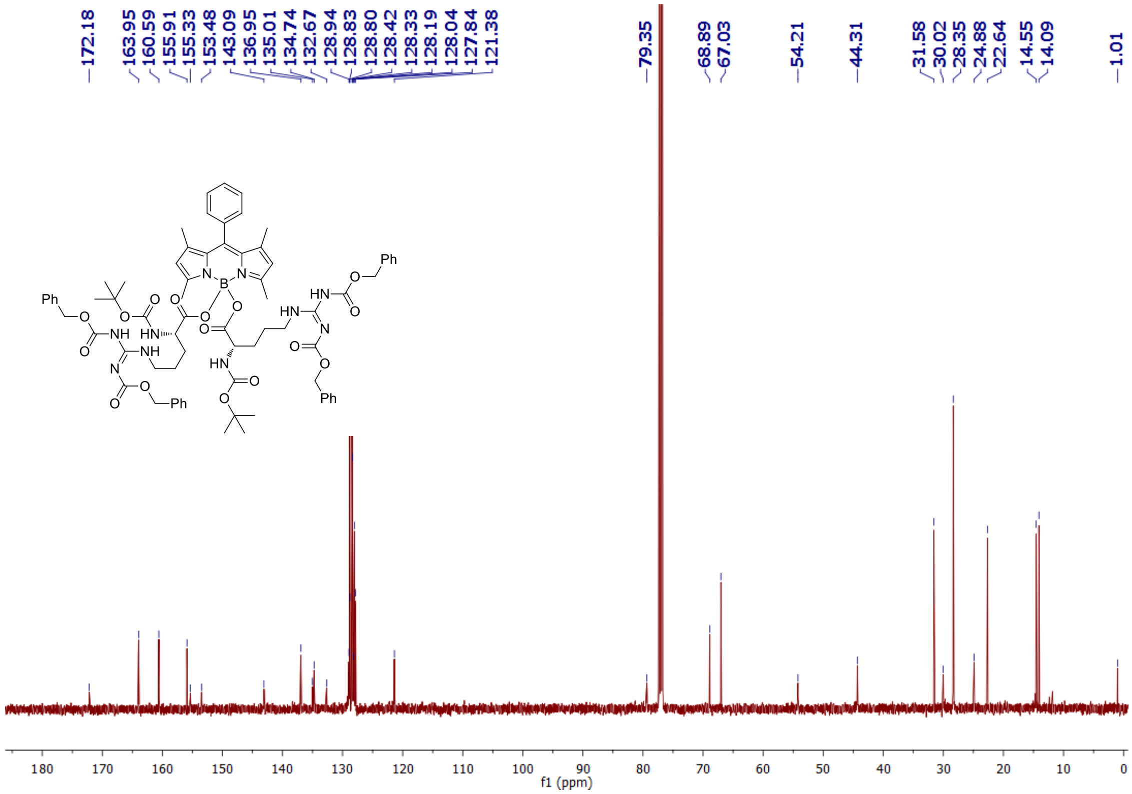

Figure S17. $^{13}C\{^{1}H\}$ NMR (100 MHz, $CDCl_3$) spectrum of compound **3C**.

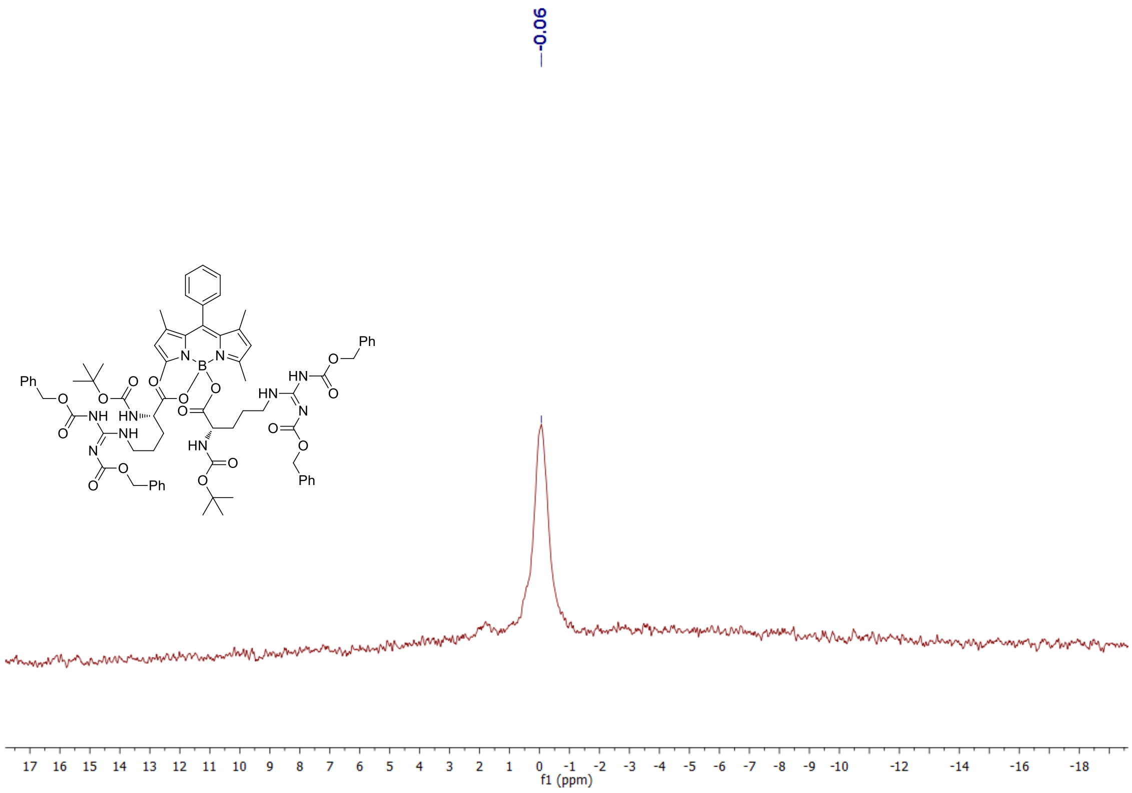

Figure S18. ^{11}B NMR (128 MHz, $CDCl_3$) spectrum of compound **3C**.

Compound 4B

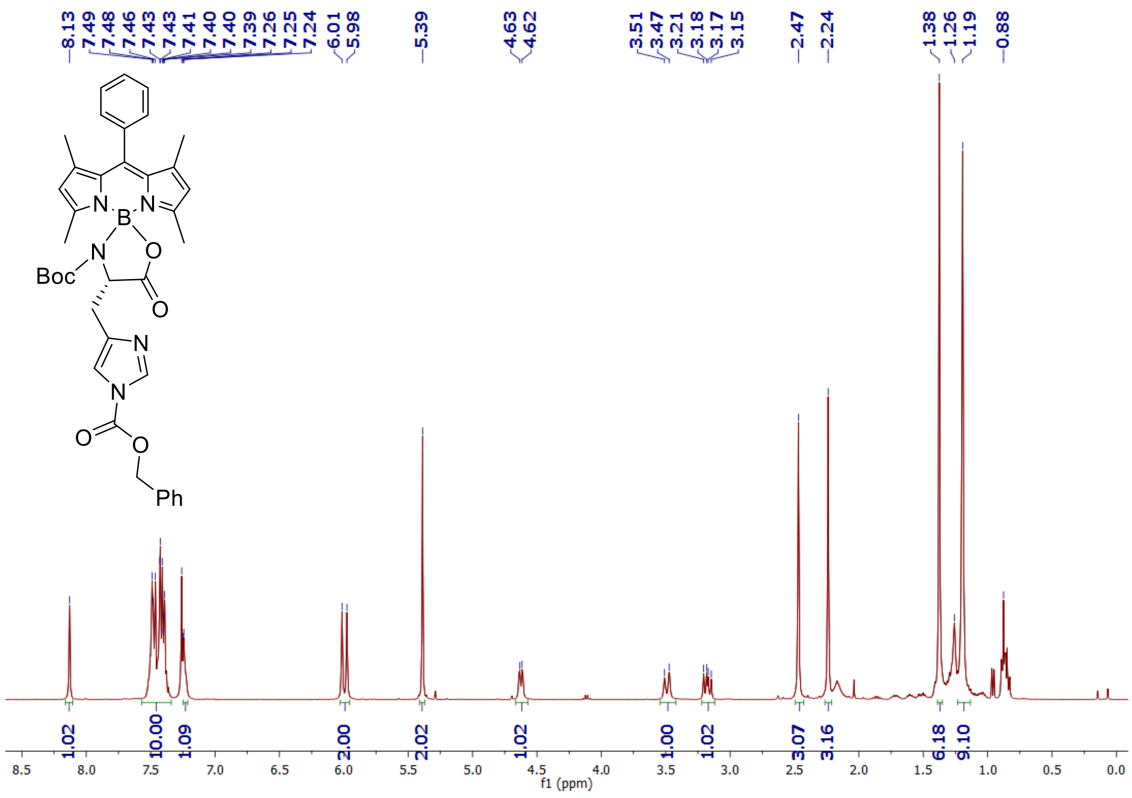

Figure S19. ^{1}H NMR (400 MHz, $CDCl_3$) spectrum of compound **4B**.

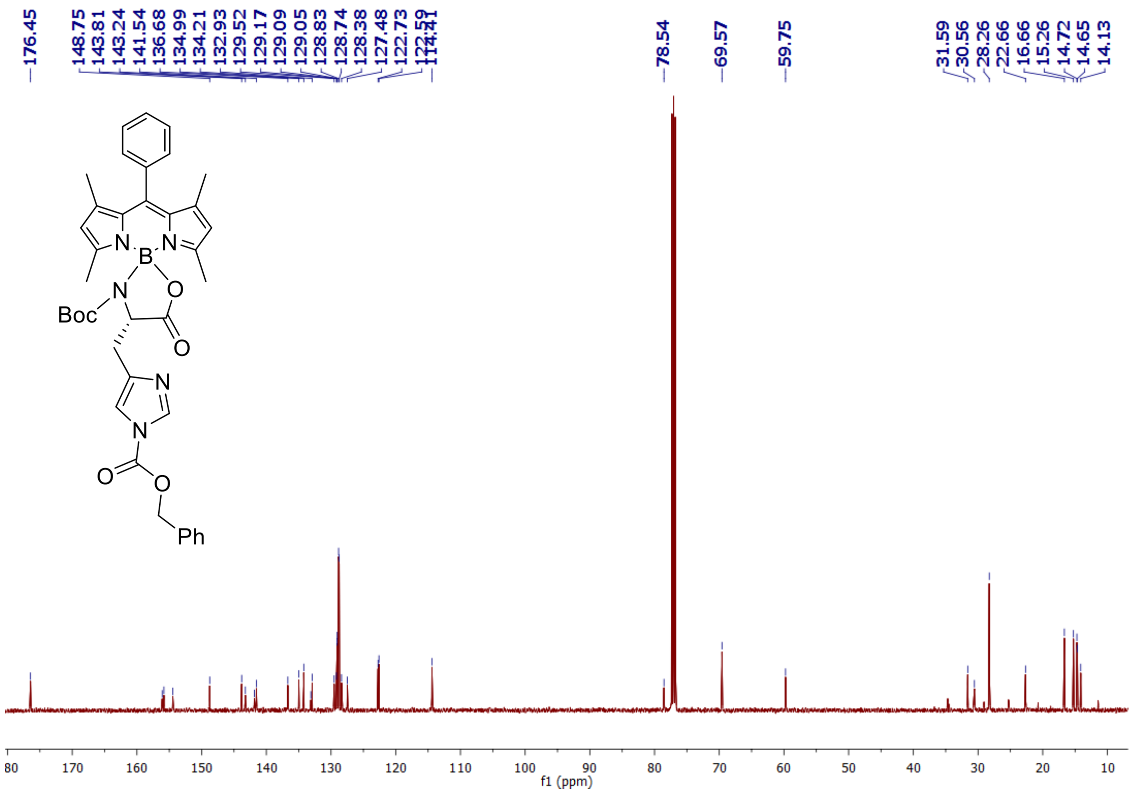

Figure S20. $^{13}C\{^{1}H\}$ NMR (100 MHz, $CDCl_3$) spectrum of compound **4B**.

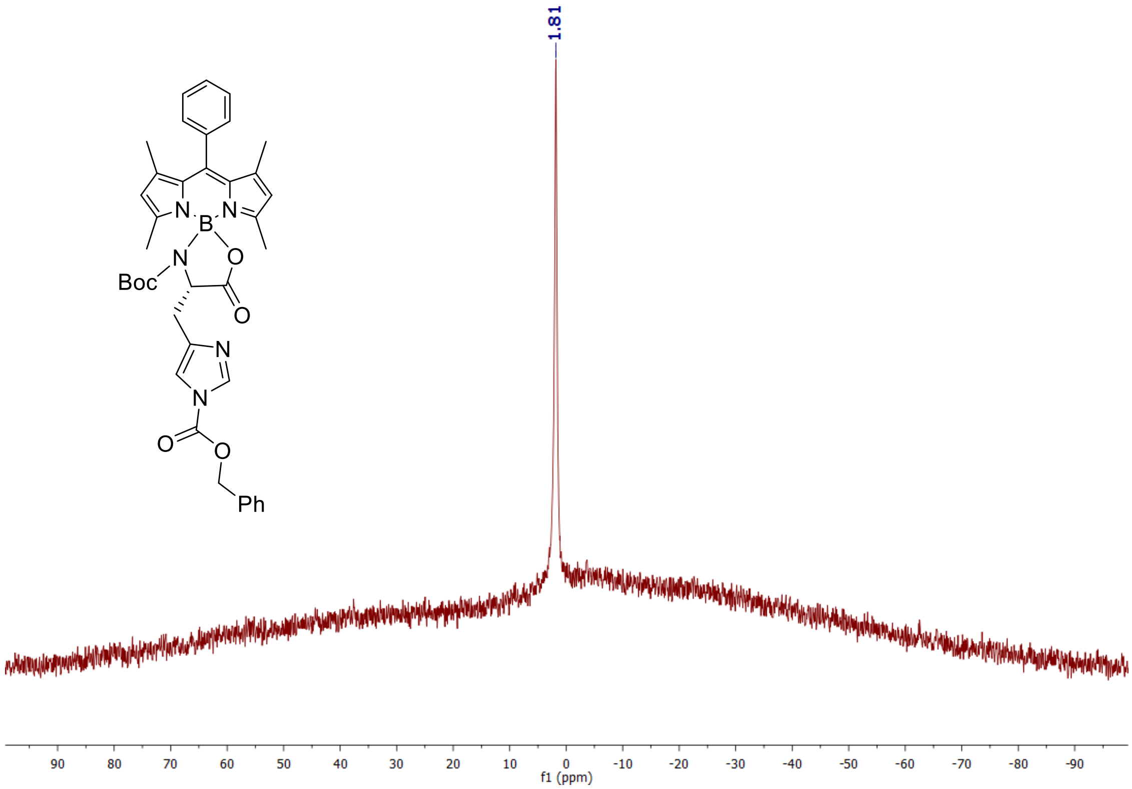

Figure S21. ^{11}B NMR (128 MHz, $CDCl_3$) spectrum of compound **4B**.

Compound 4C

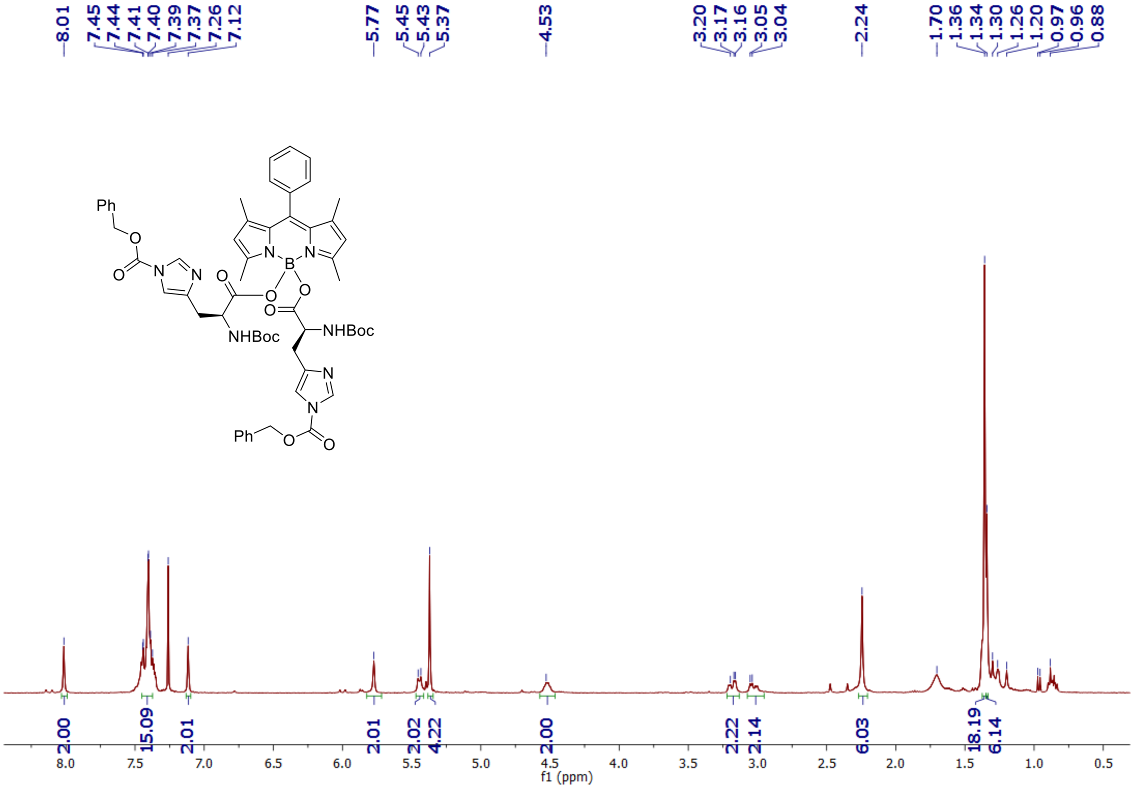

Figure S22. ^{1}H NMR (400 MHz, $CDCl_3$) spectrum of compound **4C**.

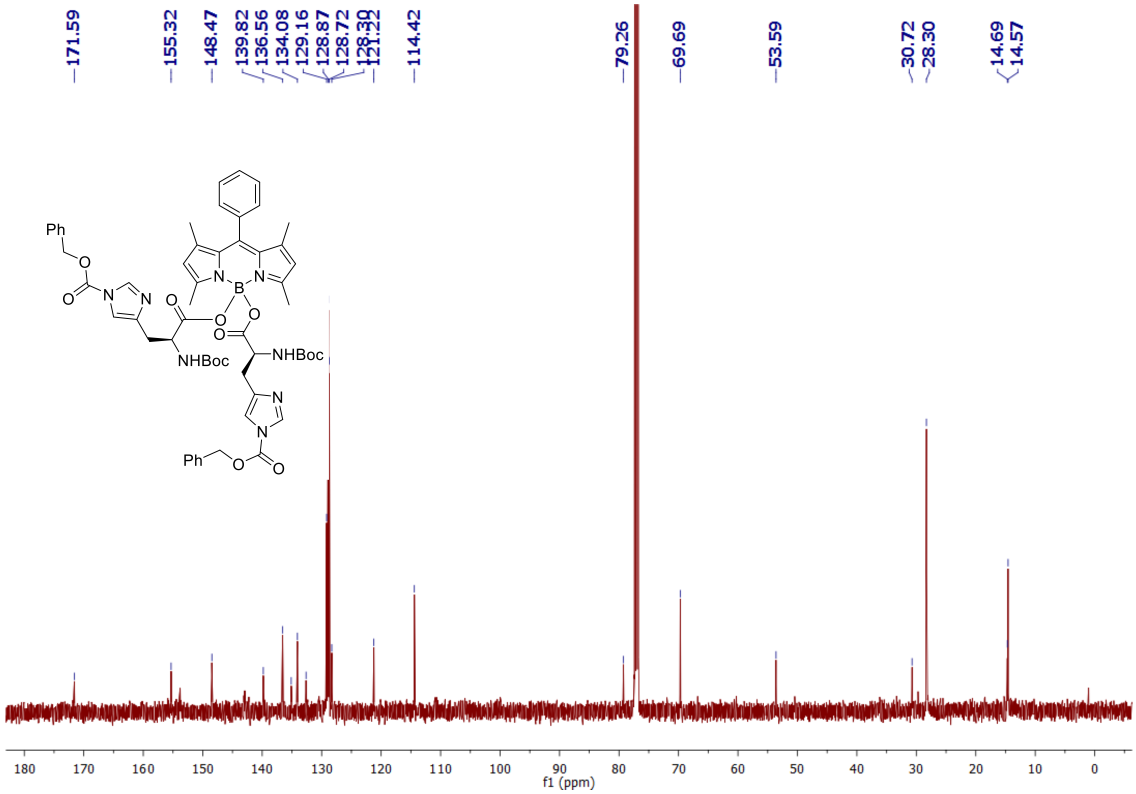

Figure S23. ${}^{13}C\{{}^{1}H\}$ NMR (100 MHz, $CDCl_3$) spectrum of compound **4C**.

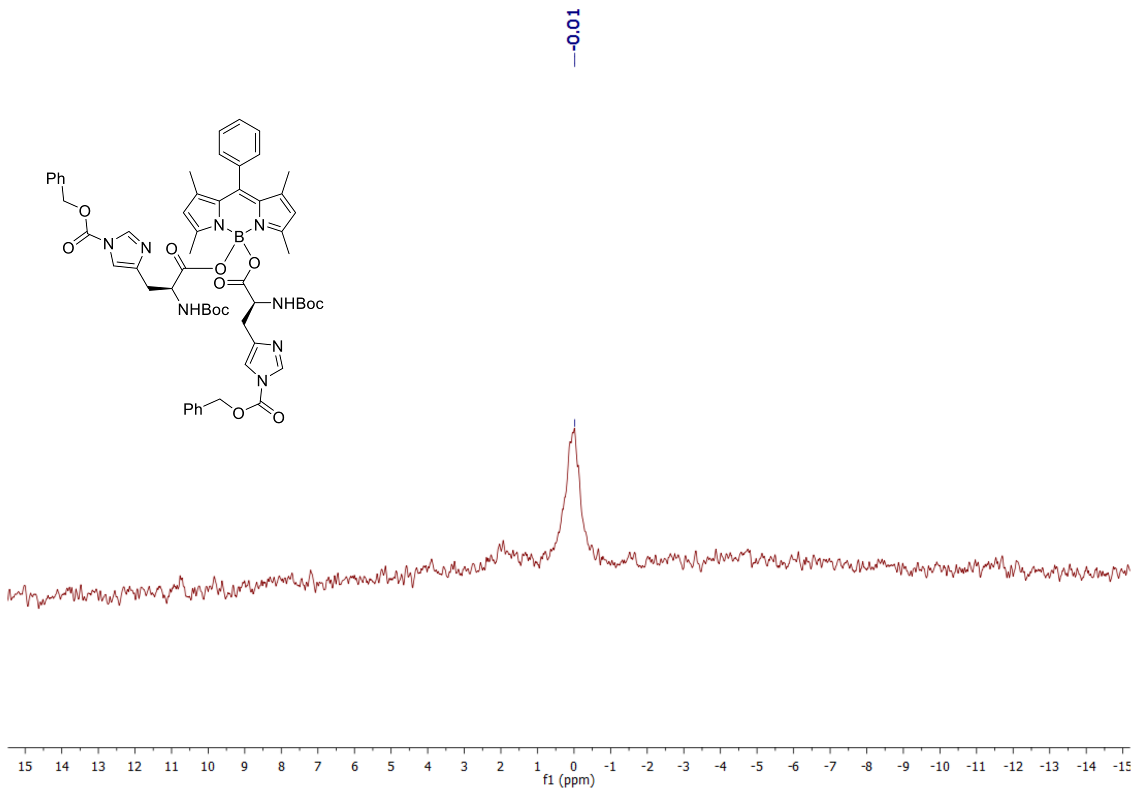

Figure S24. ${}^{11}B$ NMR (128 MHz, $CDCl_3$) spectrum of compound **4C**.

Compound 5A

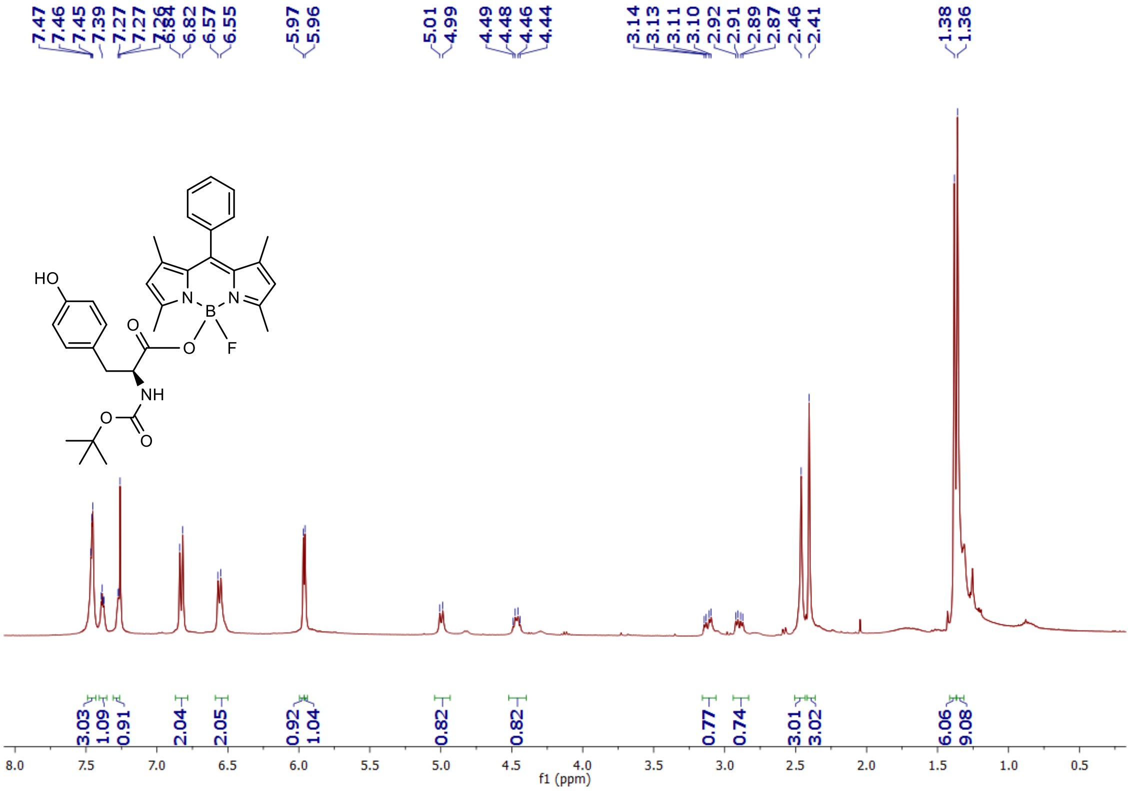

Figure S25. ^{1}H NMR (400 MHz, $CDCl_3$) spectrum of compound **5A**.

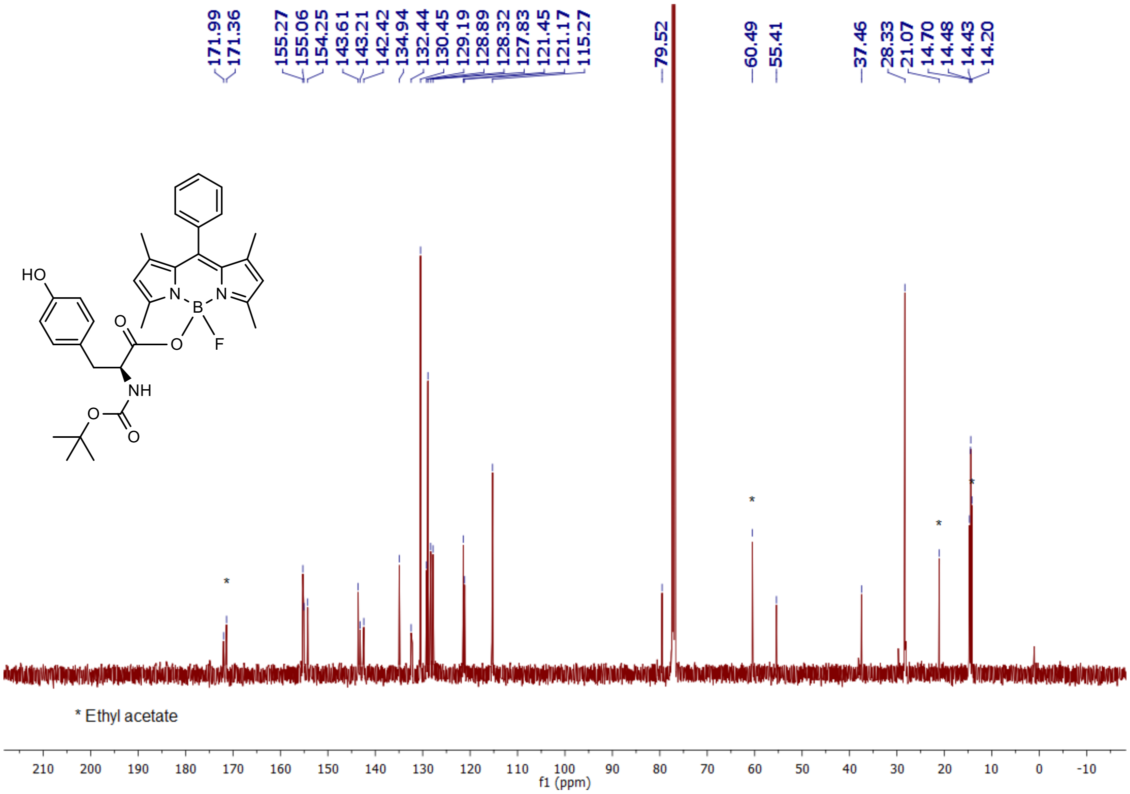

Figure S26. $^{13}C\{^{1}H\}$ NMR (100 MHz, $CDCl_3$) spectrum of compound **5A**.

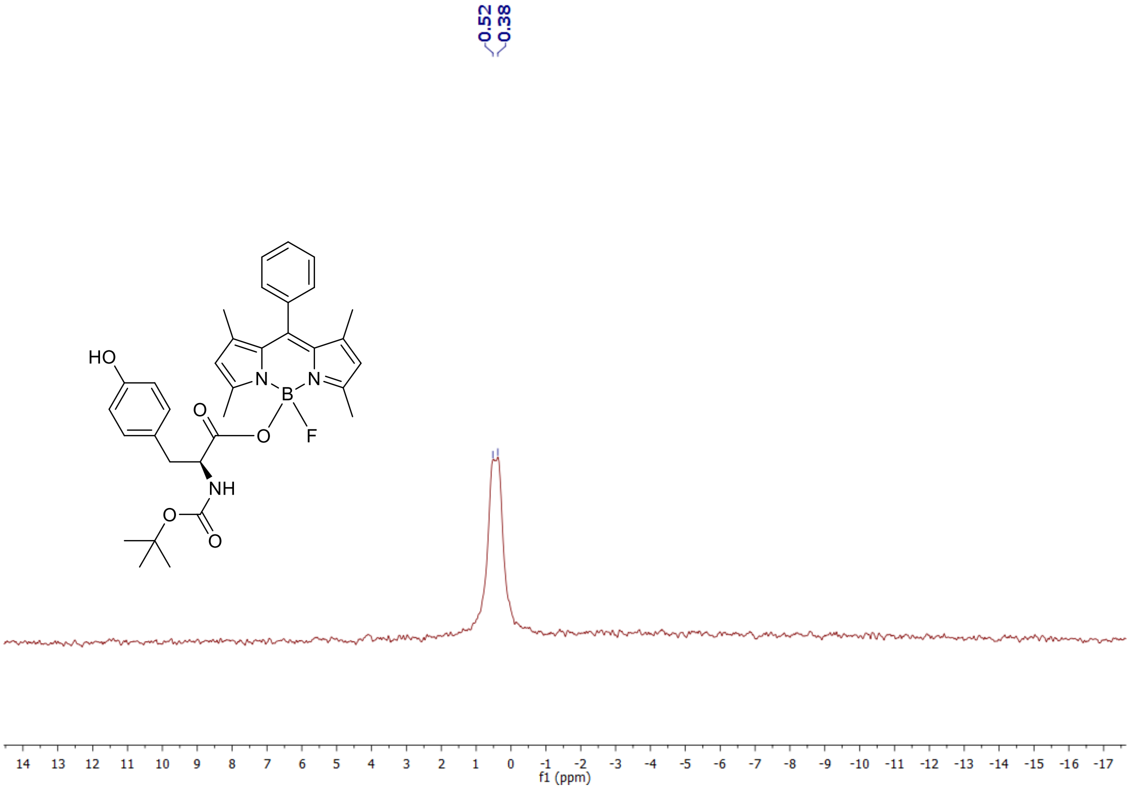

Figure S27. ^{11}B NMR (128 MHz, $CDCl_3$) spectrum of compound **5A**.

Compound 5B

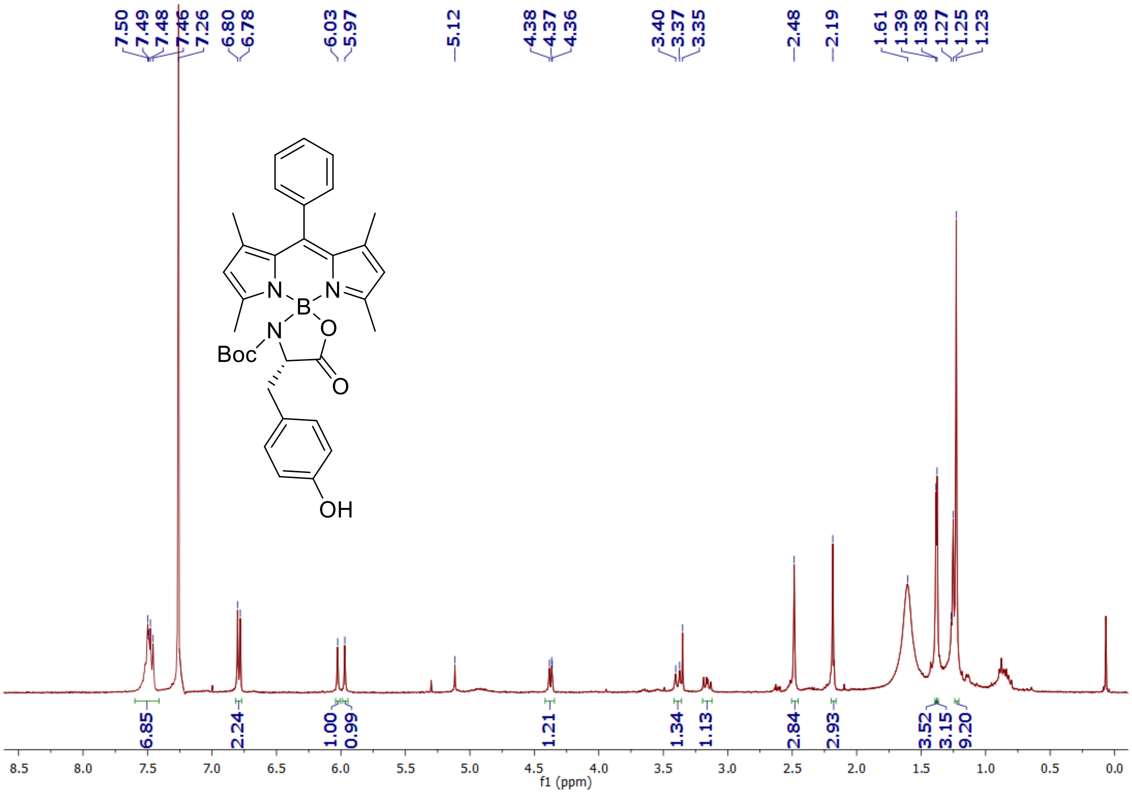

Figure S28. ^{1}H NMR (400 MHz, $CDCl_3$) spectrum of compound **5B**.

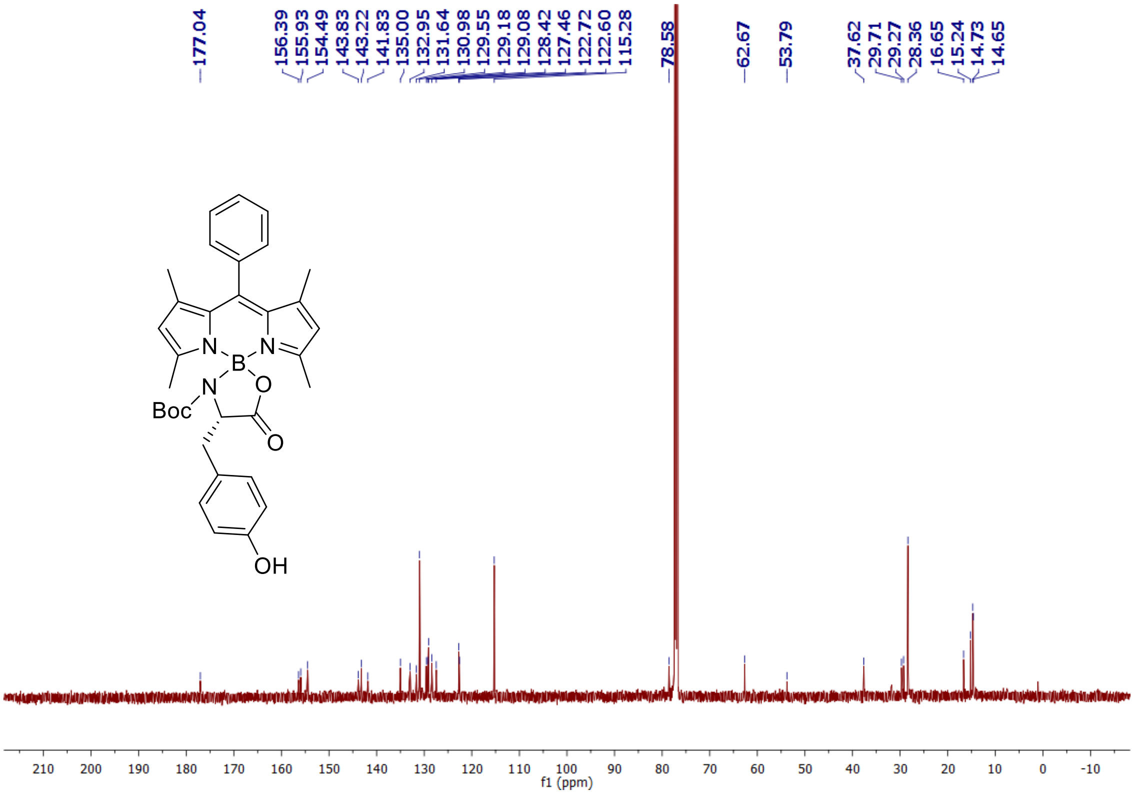

Figure S29. $^{13}C\{^{1}H\}$ NMR (100 MHz, $CDCl_3$) spectrum of compound **5B**.

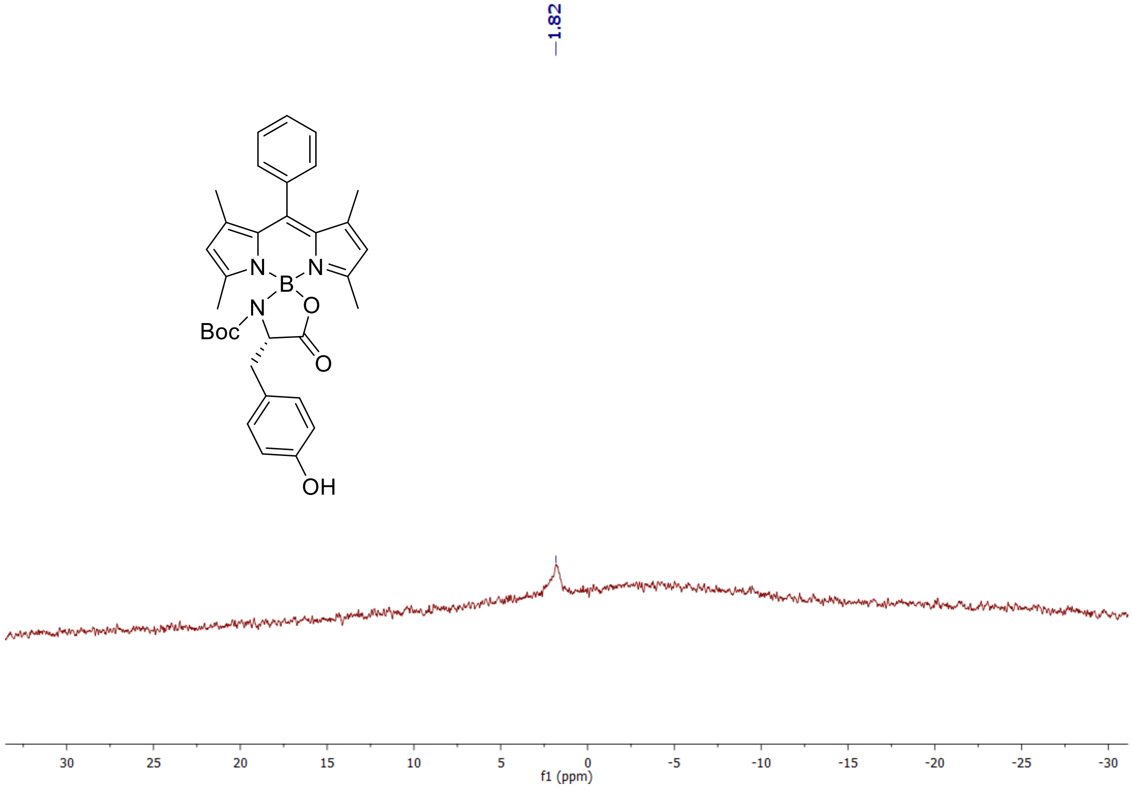

Figure S30. ^{11}B NMR (128 MHz, $CDCl_3$) spectrum of compound **5B**.

Compound 5C

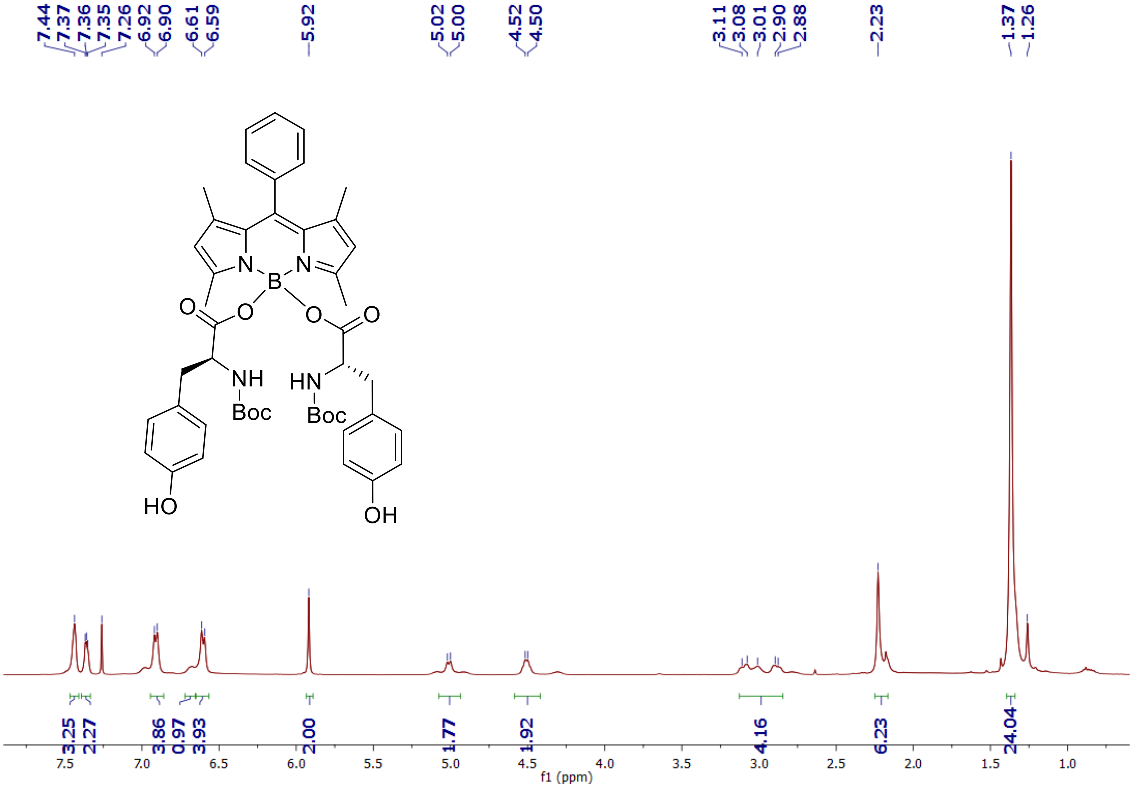

Figure S31. ^{1}H NMR (400 MHz, $CDCl_3$) spectrum of compound **5C**.

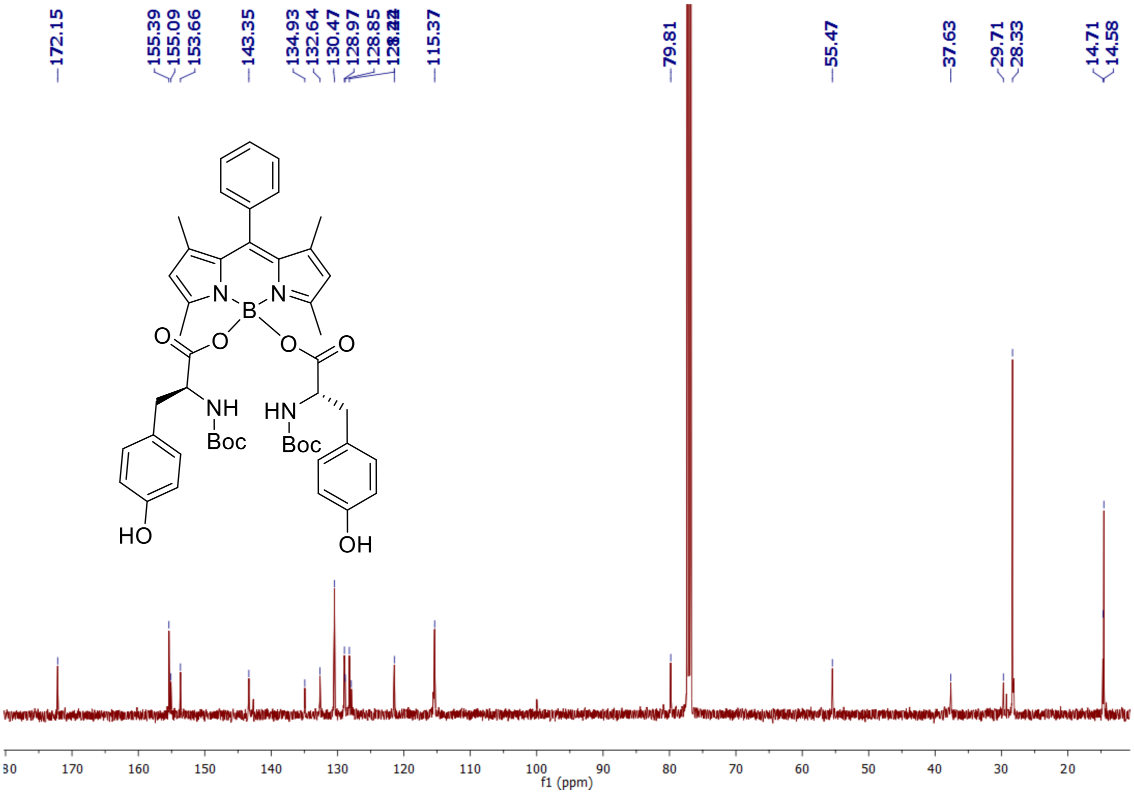

Figure S32. $^{13}C\{^{1}H\}$ NMR (100 MHz, $CDCl_3$) spectrum of compound **5C**.

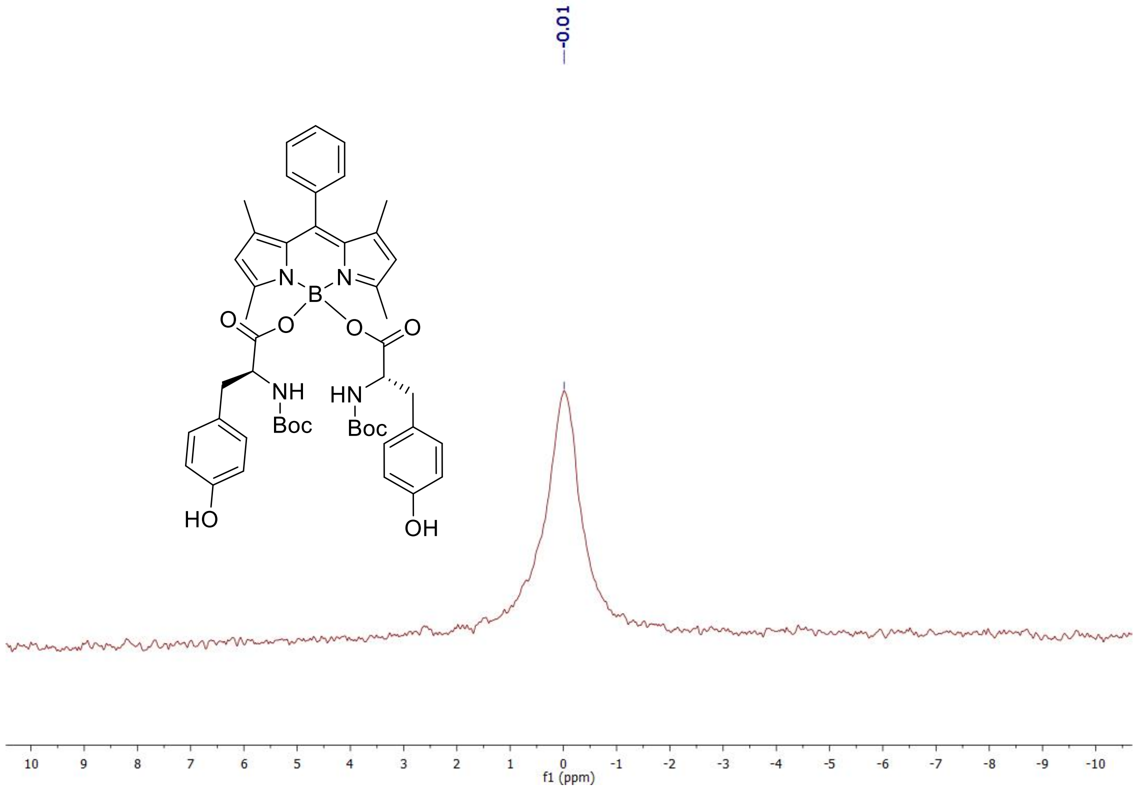

Figure S33. ^{11}B NMR (128 MHz, $CDCl_3$) spectrum of compound **5C**.

Compound 6B

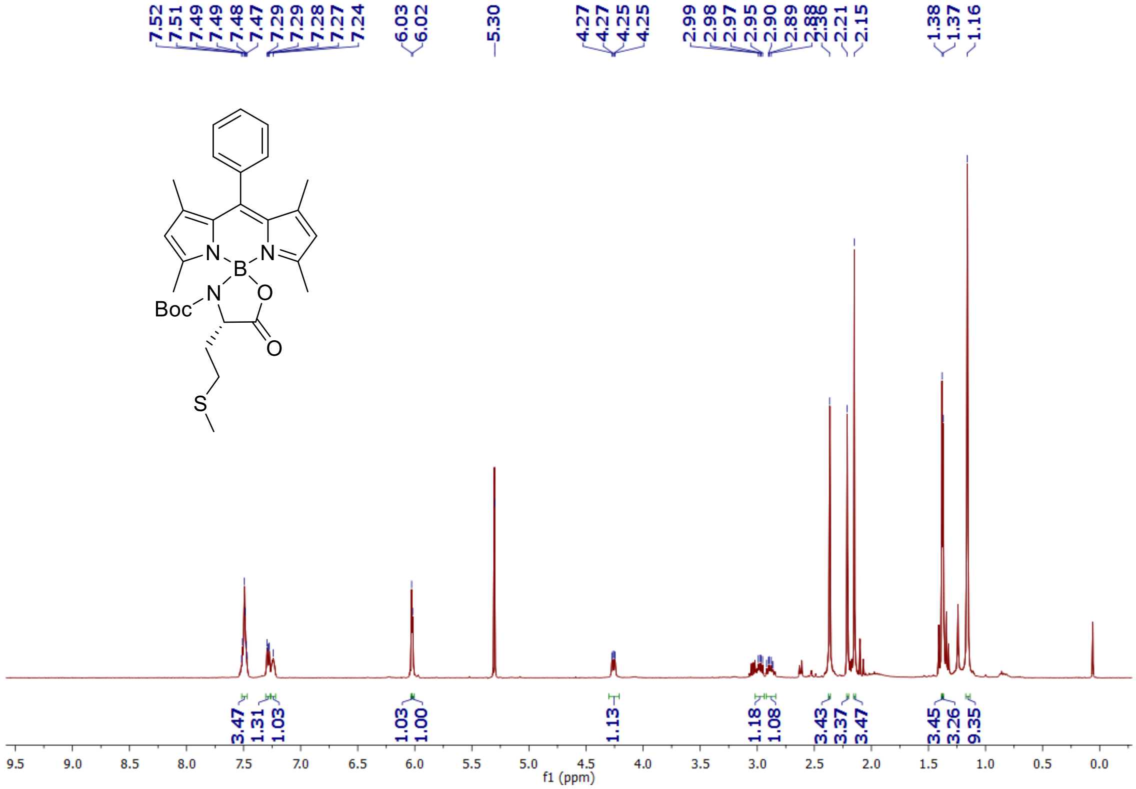

Figure S34. ^{1}H NMR (400 MHz, CD_2Cl_2) spectrum of compound **6B**.

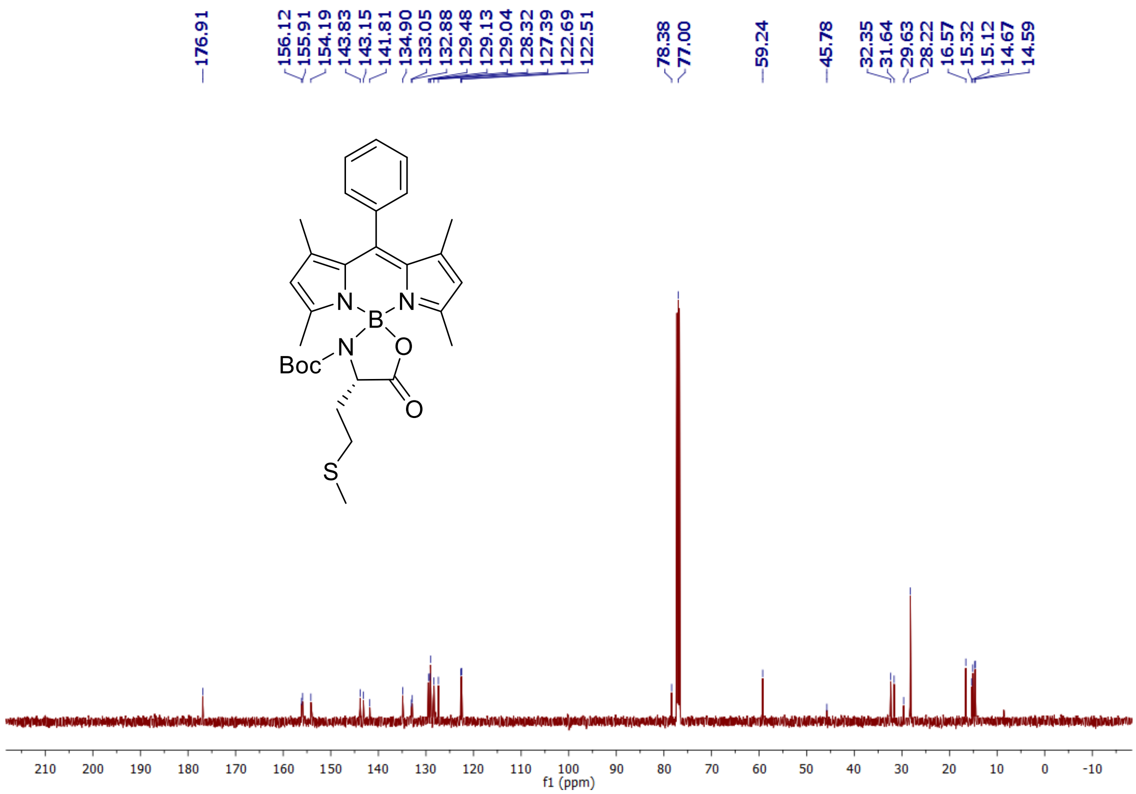

Figure S35. $^{13}C\{^{1}H\}$ NMR (100 MHz, $CDCl_3$) spectrum of compound **6B**.

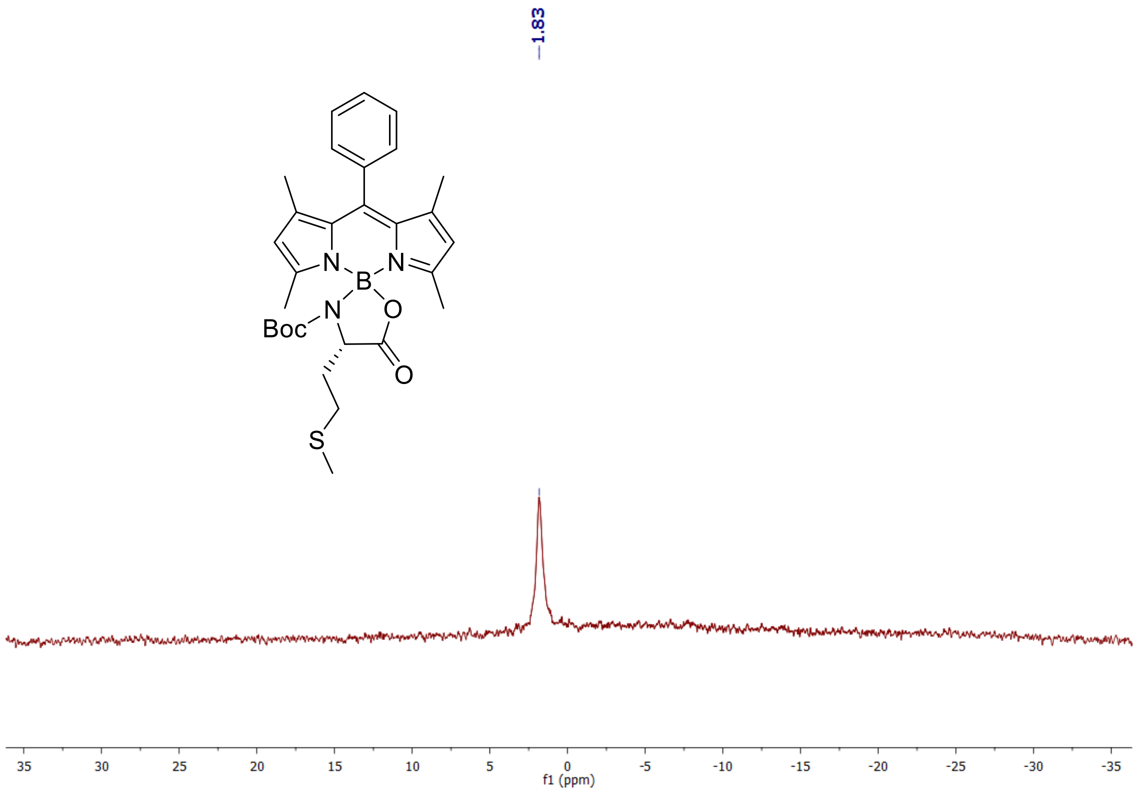

Figure S36. ^{11}B NMR (128 MHz, $CDCl_3$) spectrum of compound **6B**.

Compound 6C

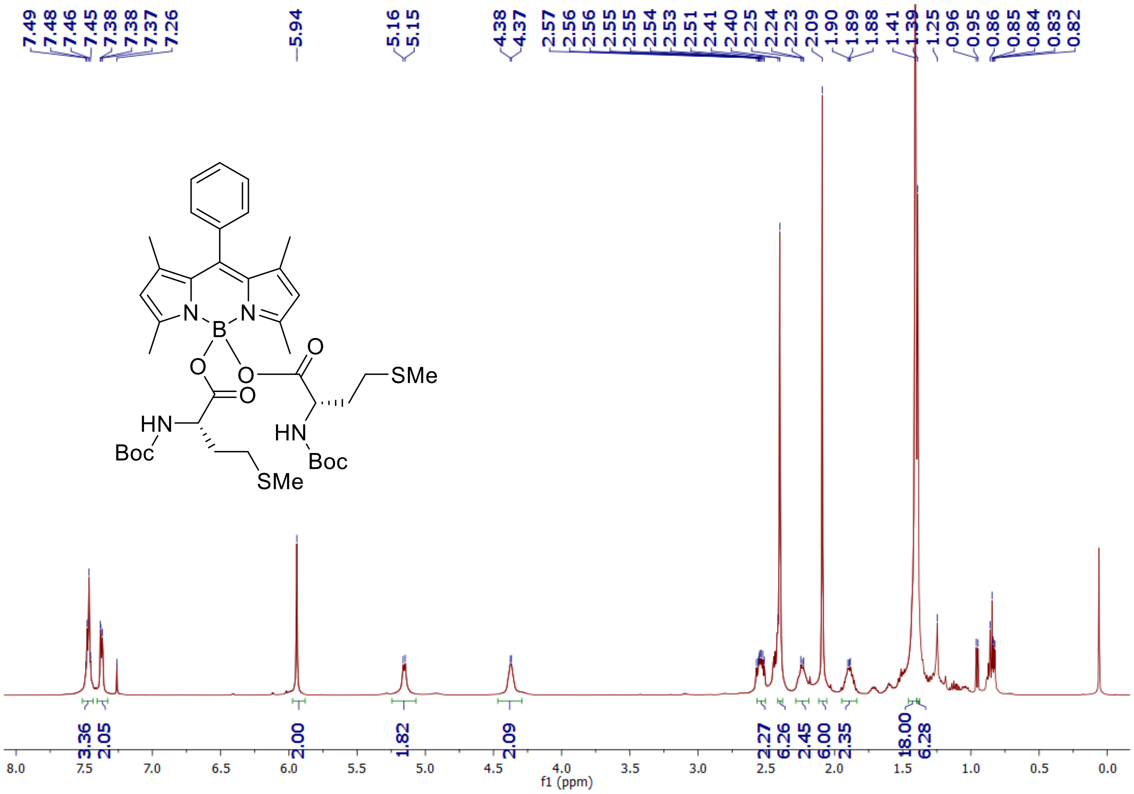

Figure S37. ^{1}H NMR (400 MHz, $CDCl_3$) spectrum of compound **6C**.

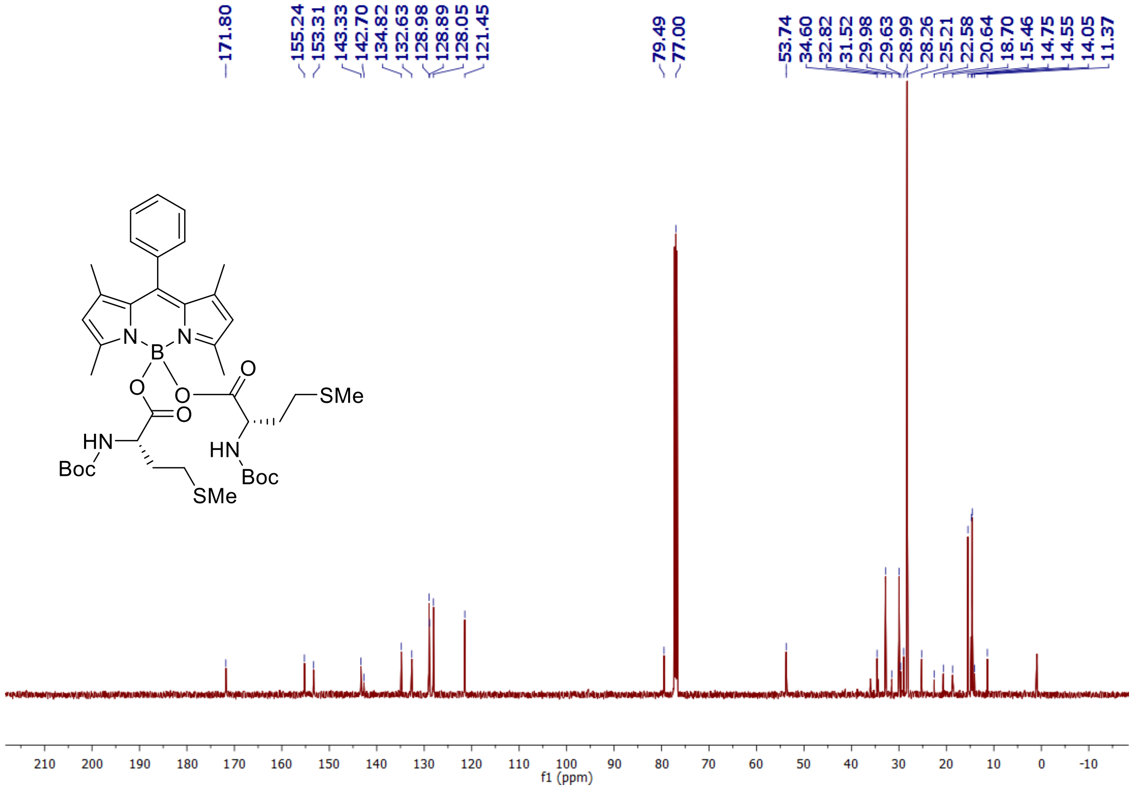

Figure S38. $^{13}C\{^{1}H\}$ NMR (100 MHz, $CDCl_3$) spectrum of compound **6C**.

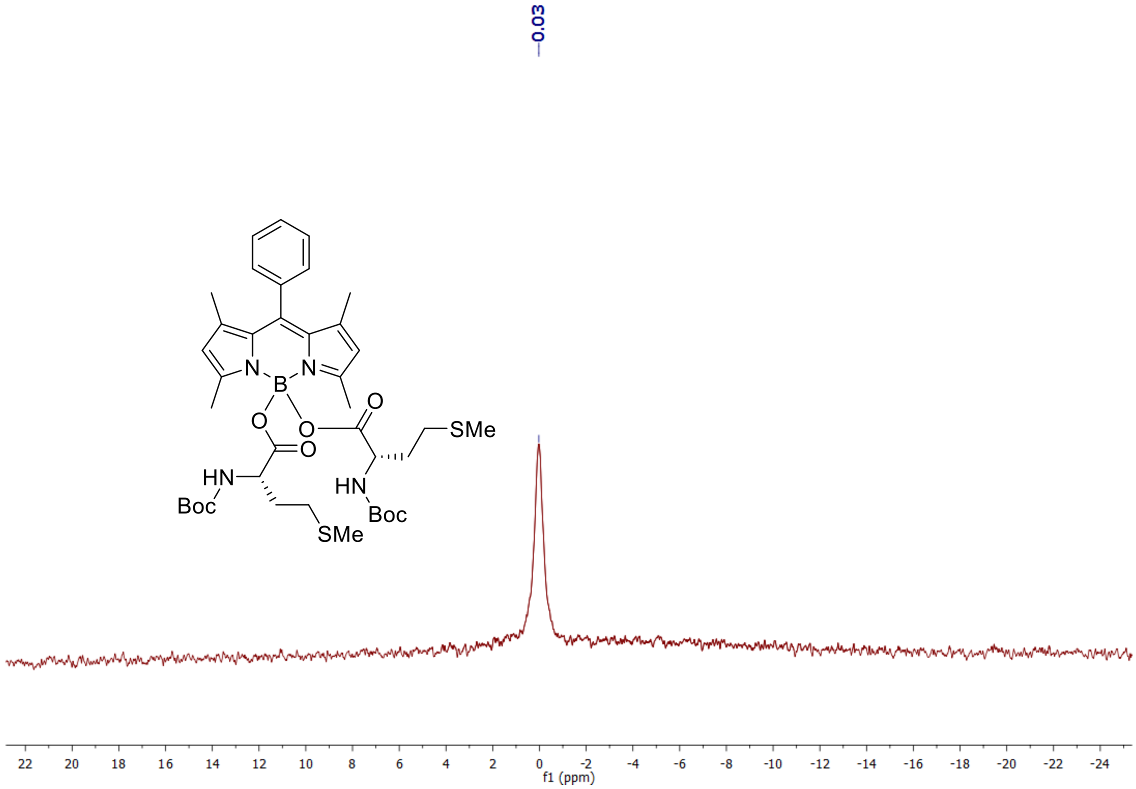

Figure S39. ^{11}B NMR (128 MHz, $CDCl_3$) spectrum of compound **6C**.

Compound 7A

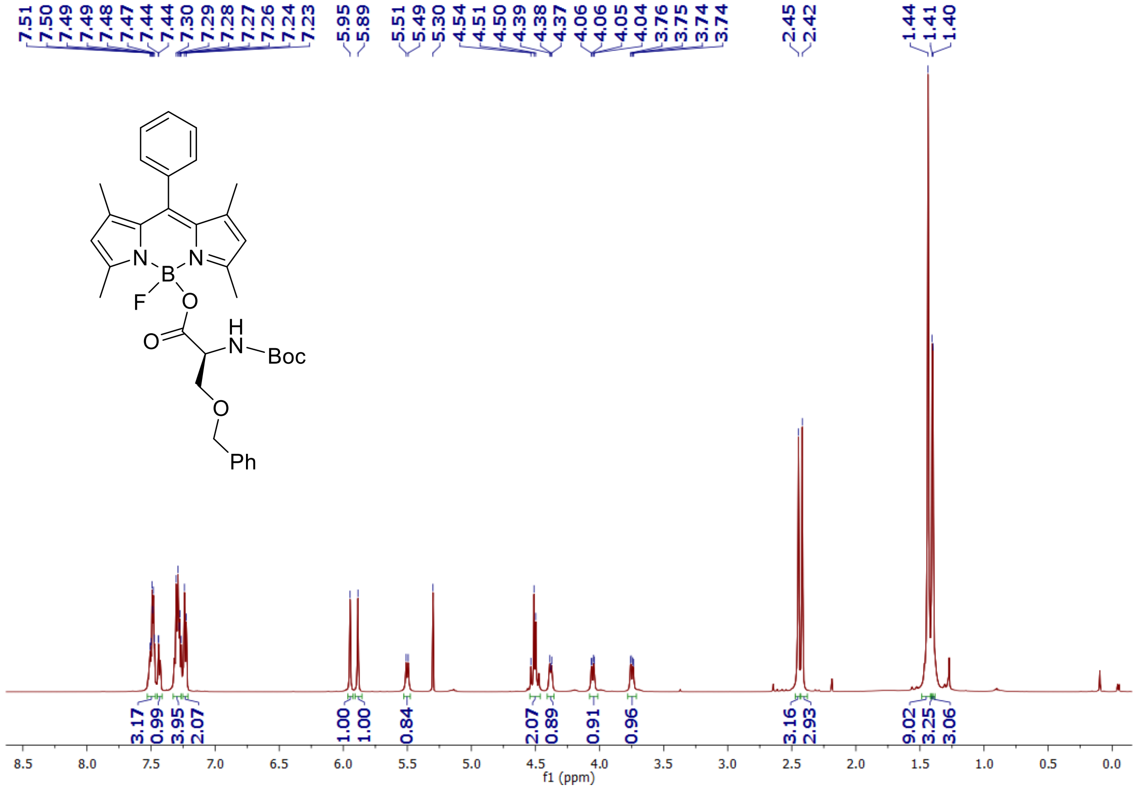

Figure S40. ^{1}H NMR (400 MHz, $CDCl_3$) spectrum of compound **7A**.

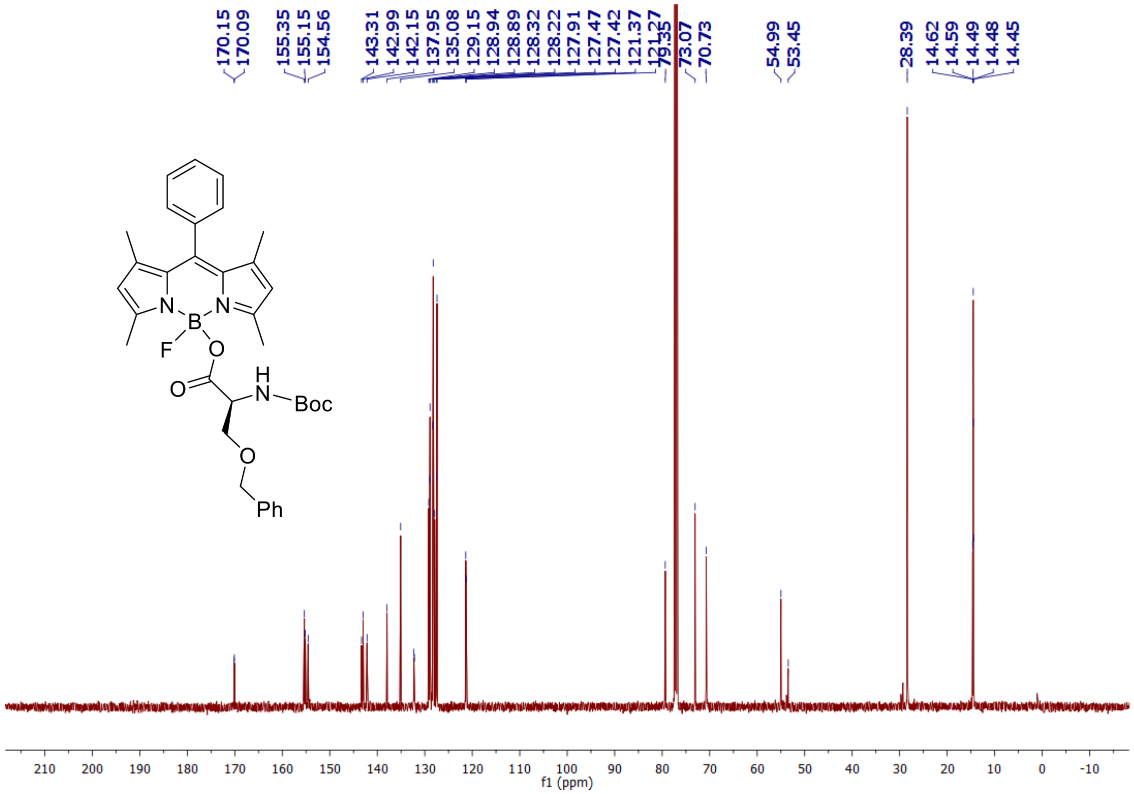

Figure S41. $^{13}C\{^{1}H\}$ NMR (100 MHz, $CDCl_3$) spectrum of compound **7A**.

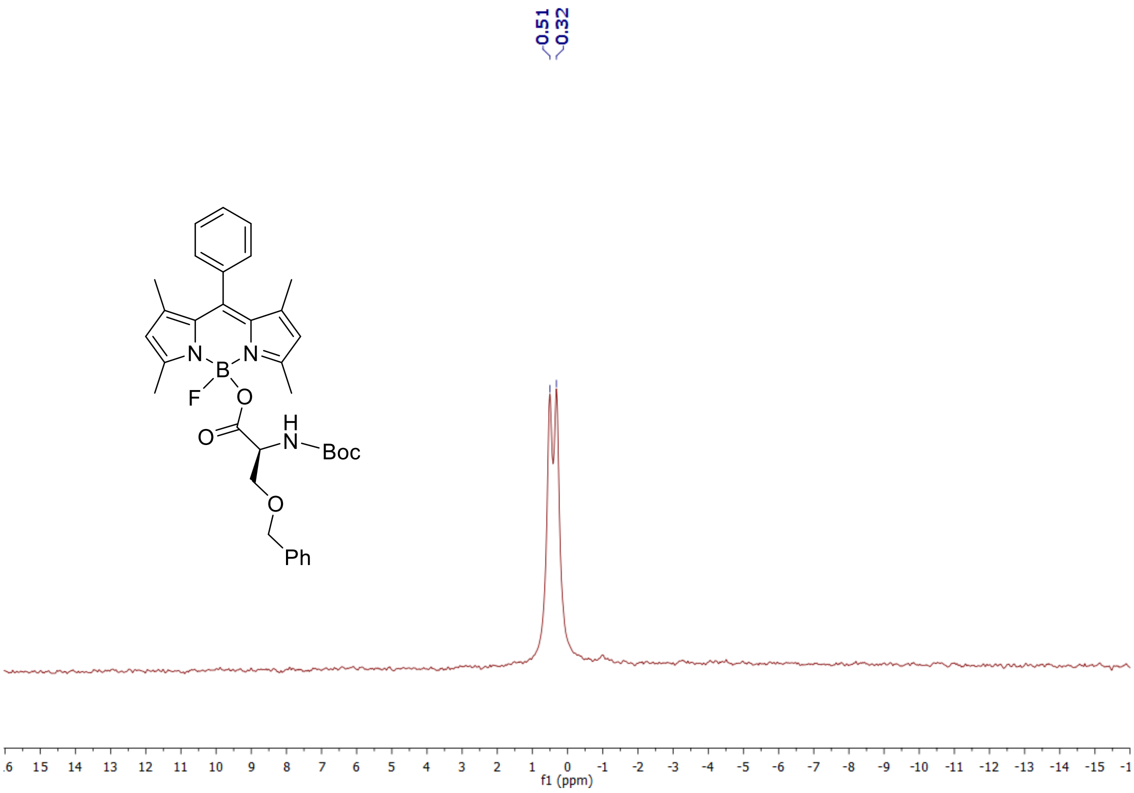

Figure S42. ^{11}B NMR (128 MHz, $CDCl_3$) spectrum of compound **7A**.

Compound 7B

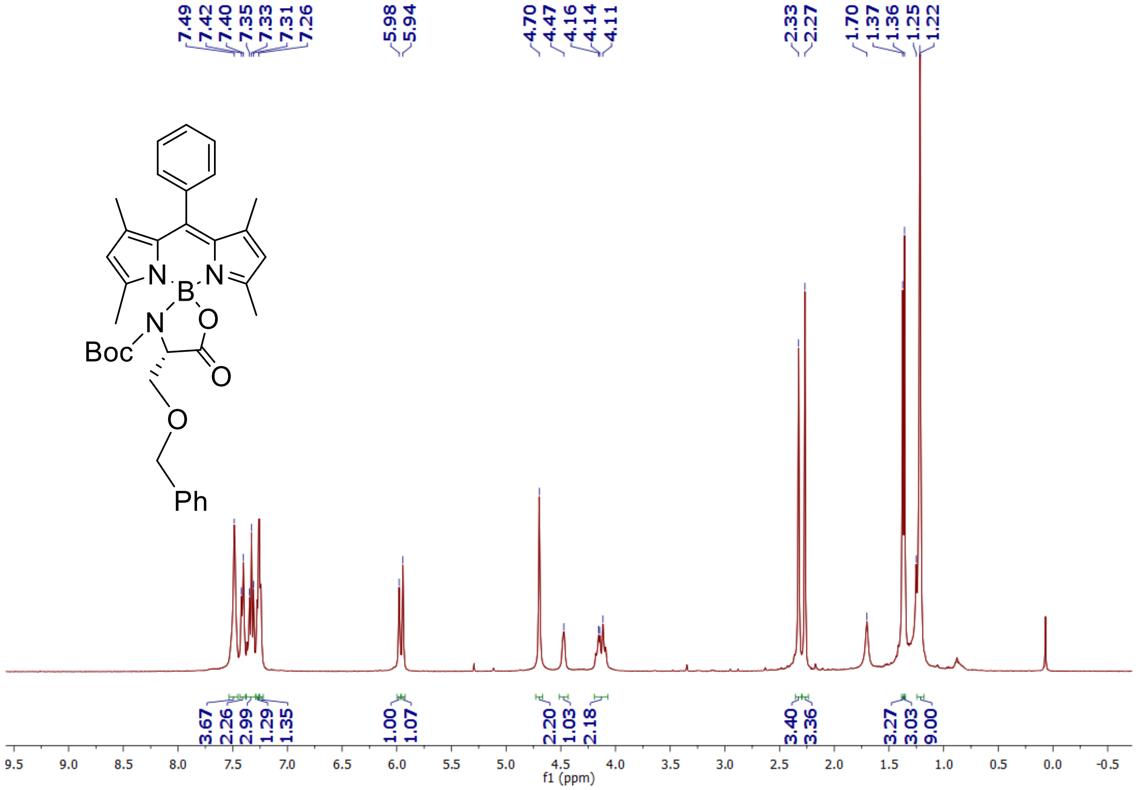

Figure S43. ^{1}H NMR (400 MHz, $CDCl_3$) spectrum of compound **7B**.

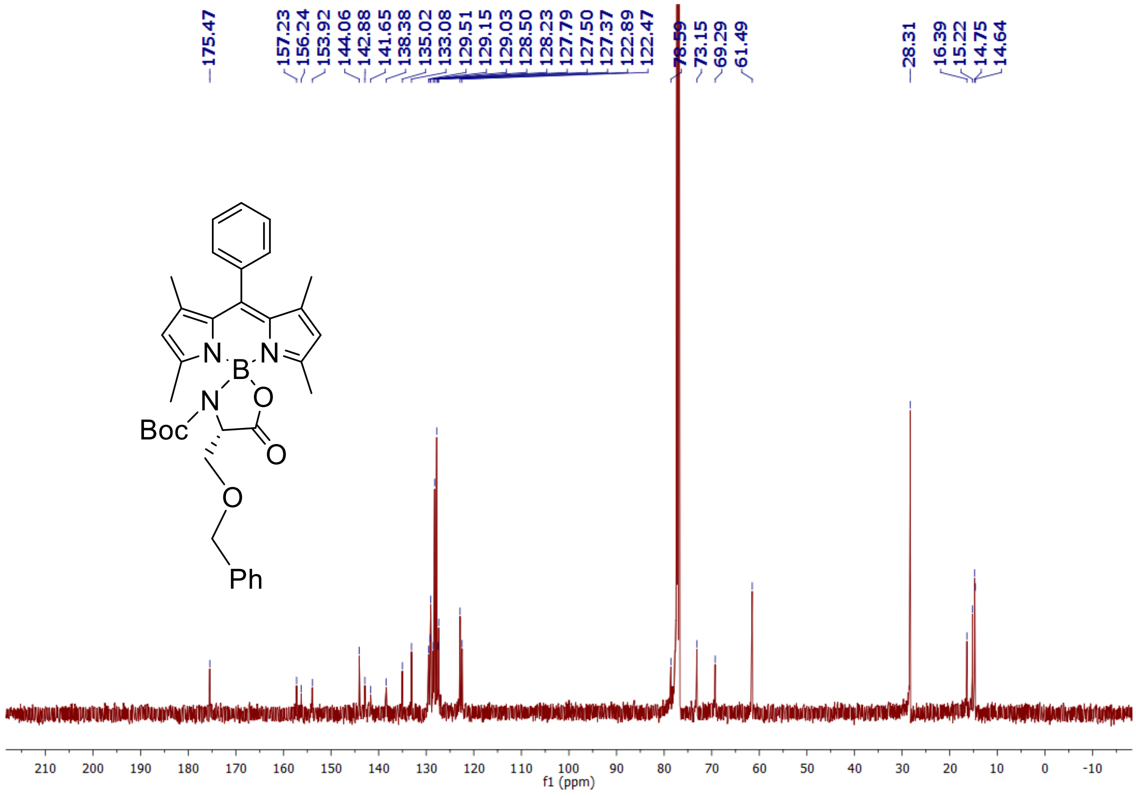

Figure S44. $^{13}C\{^{1}H\}$ NMR (100 MHz, $CDCl_3$) spectrum of compound **7B**.

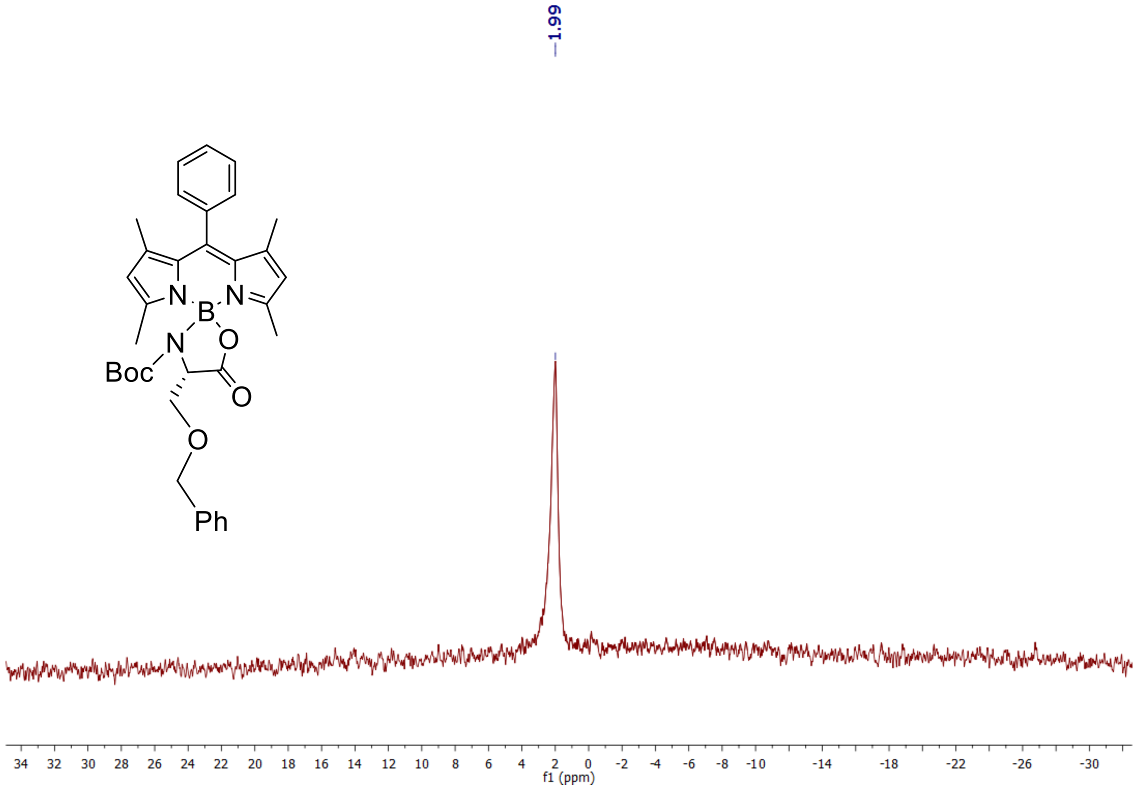

Figure S45. ^{11}B NMR (128 MHz, $CDCl_3$) spectrum of compound **7B**.

Compound 7C

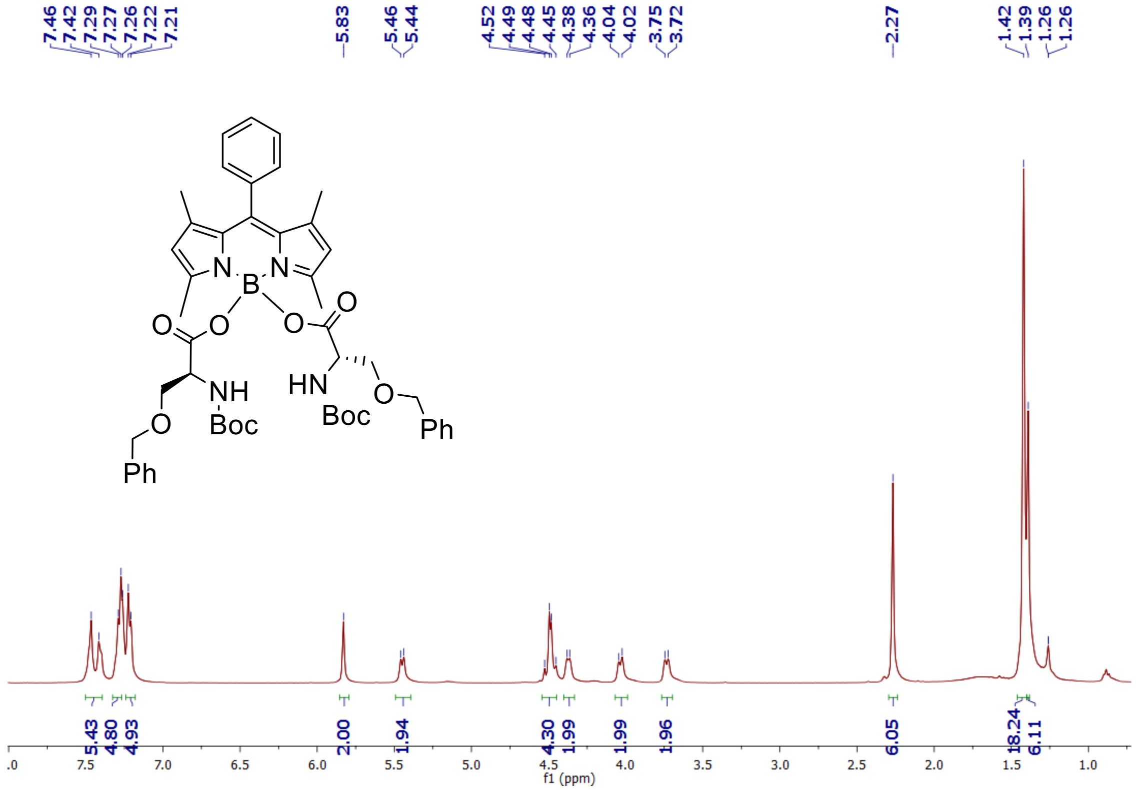

Figure S46. ^{1}H NMR (400 MHz, $CDCl_3$) spectrum of compound **7C**.

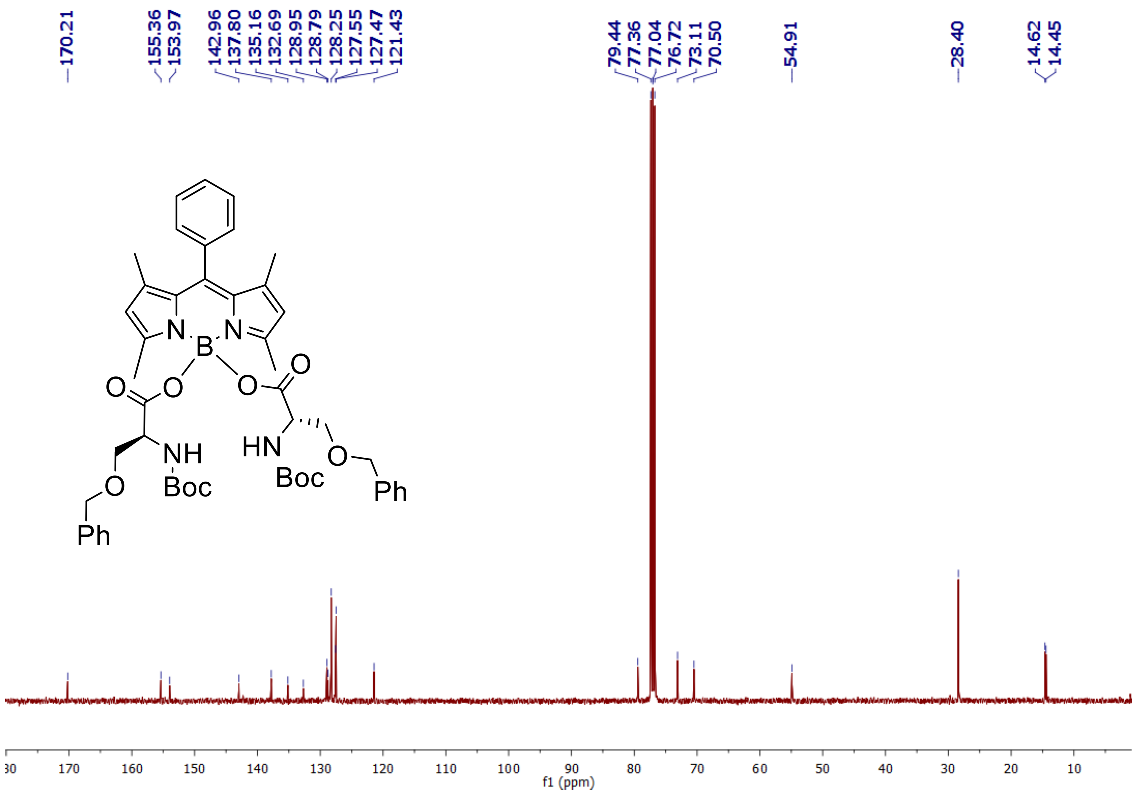

Figure S47. $^{13}C\{^{1}H\}$ NMR (100 MHz, $CDCl_3$) spectrum of compound **7C**.

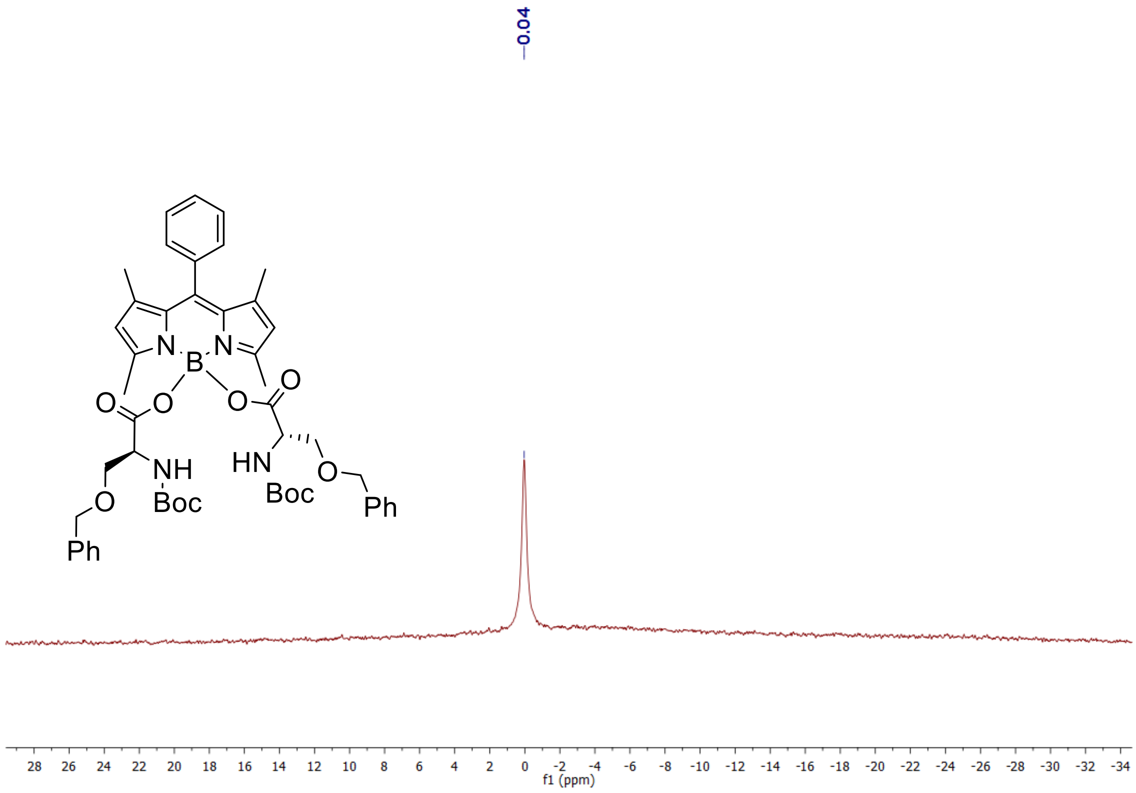

Figure S48. ^{11}B NMR (128 MHz, $CDCl_3$) spectrum of compound **7C**.

Compound 8A

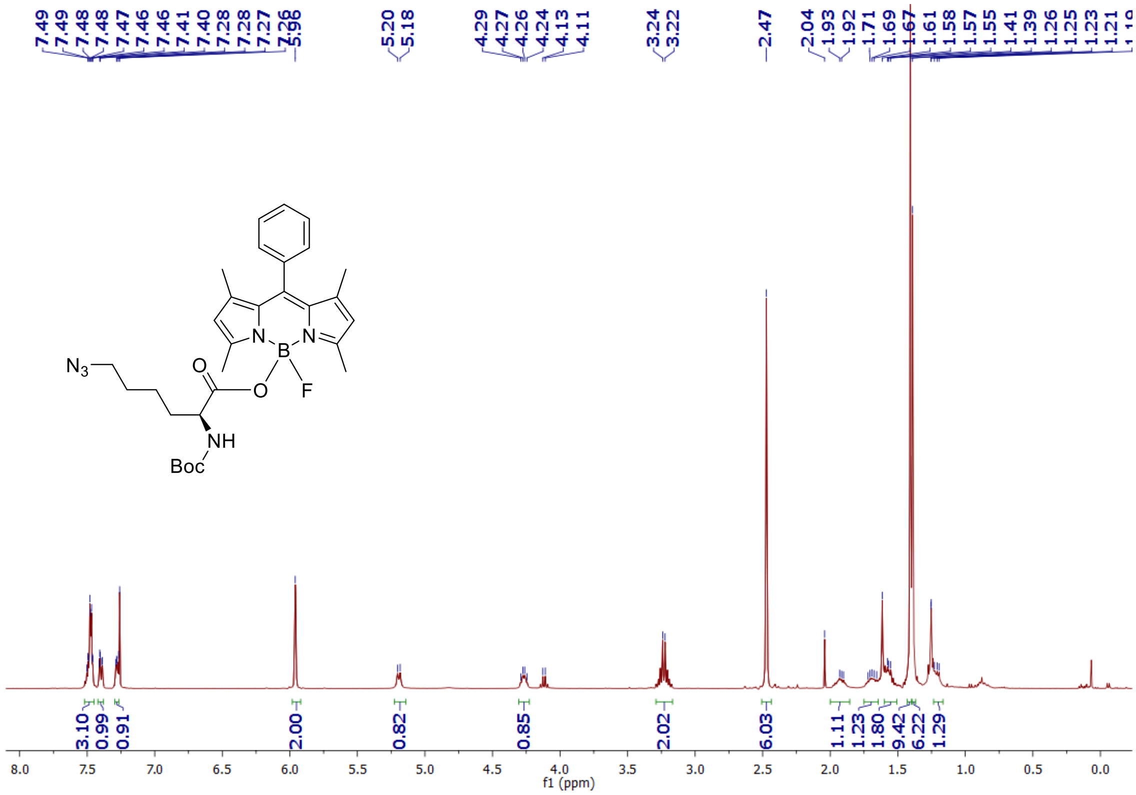

Figure S49. ^{1}H NMR (400 MHz, $CDCl_3$) spectrum of compound **8A**.

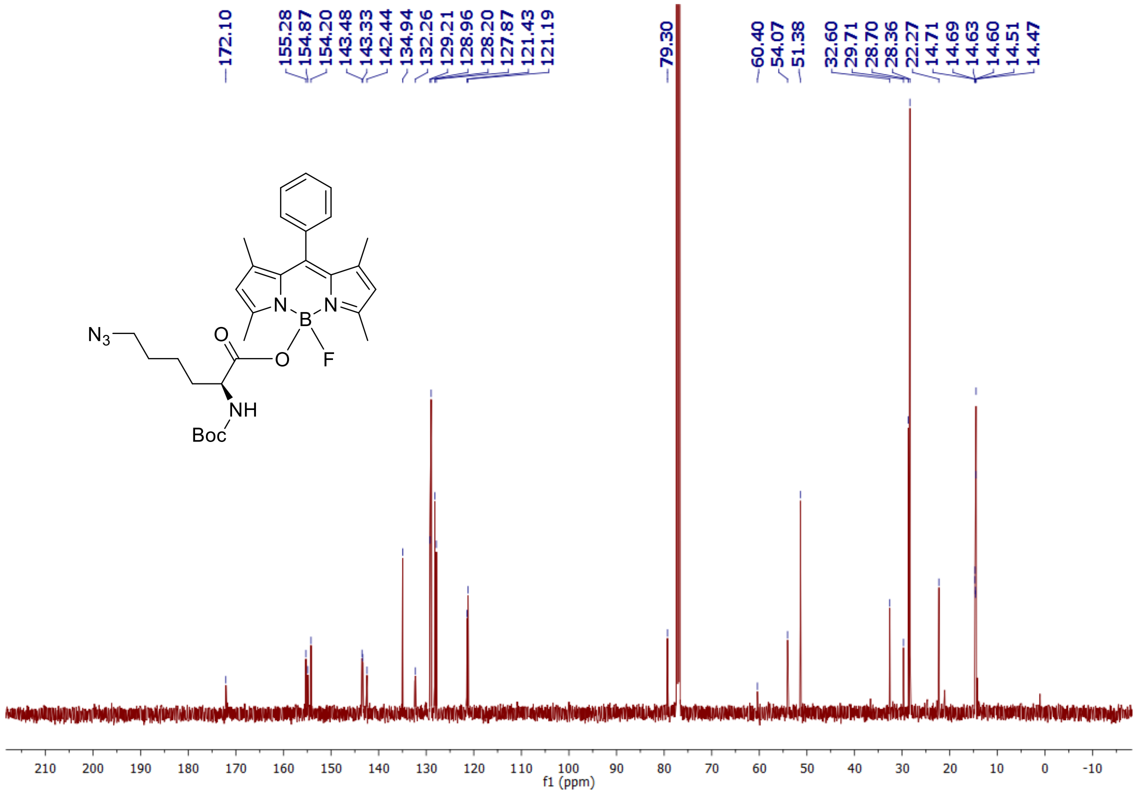

Figure S50. $^{13}C\{^{1}H\}$ NMR (100 MHz, $CDCl_3$) spectrum of compound **8A**.

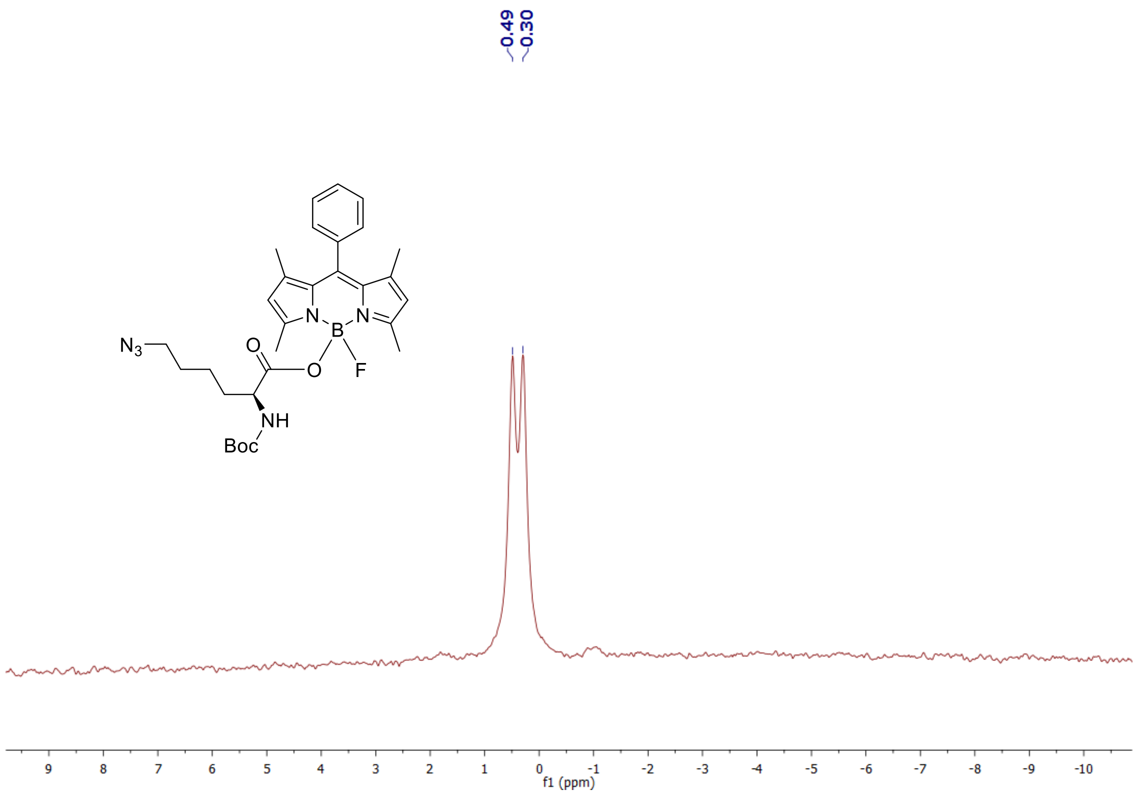

Figure S51. ^{11}B NMR (128 MHz, $CDCl_3$) spectrum of compound **8A**.

Compound 8B

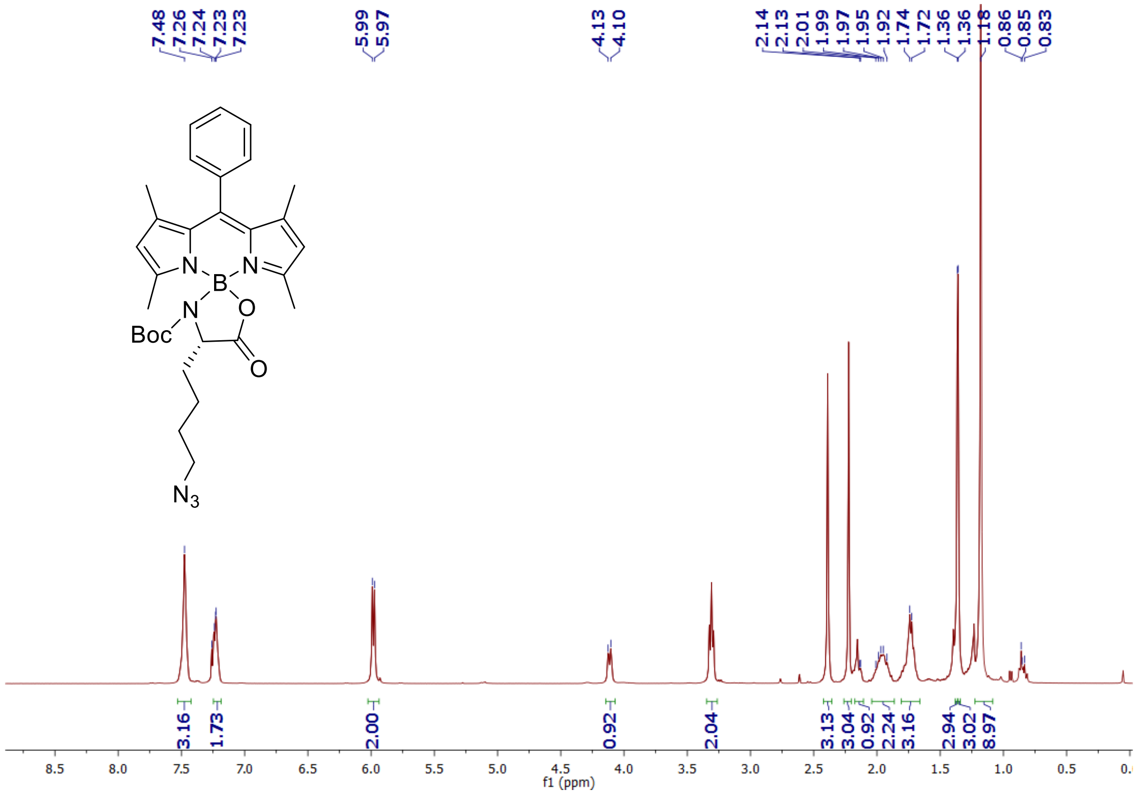

Figure S52. ^{1}H NMR (400 MHz, $CDCl_3$) spectrum of compound **8B**.

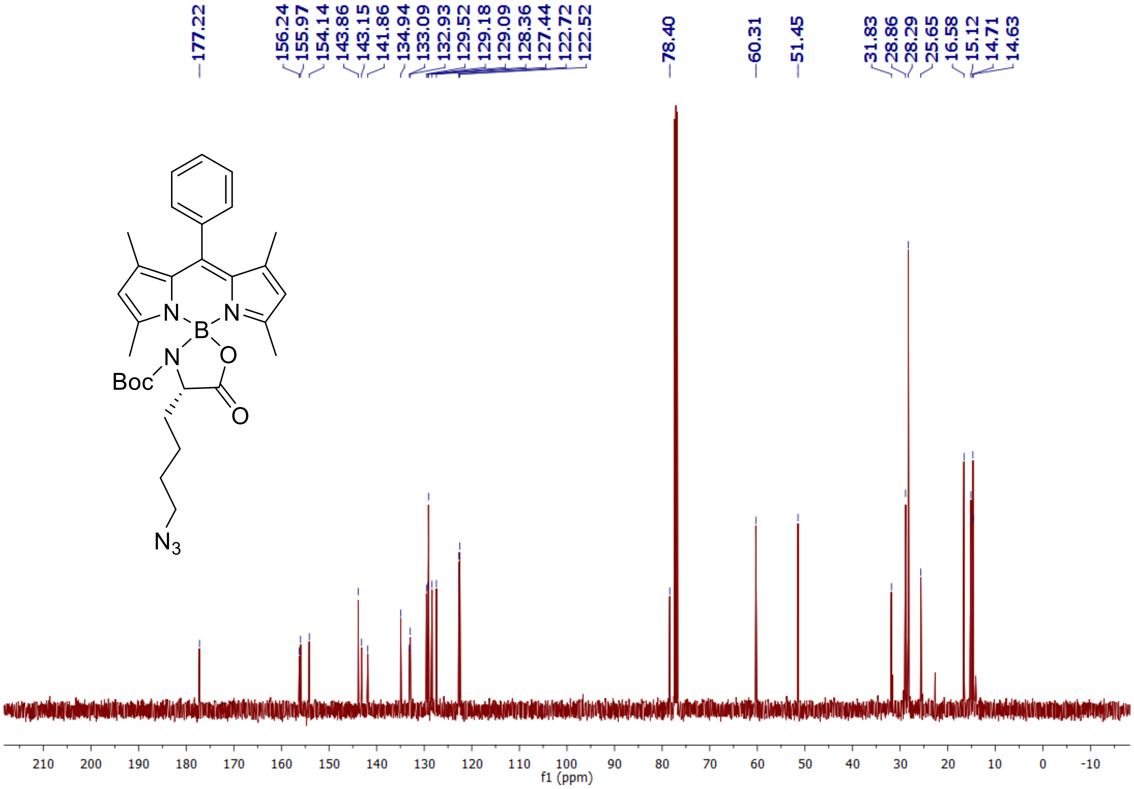

Figure S53. $^{13}C\{^1H\}$ NMR (100 MHz, $CDCl_3$) spectrum of compound **8B**.

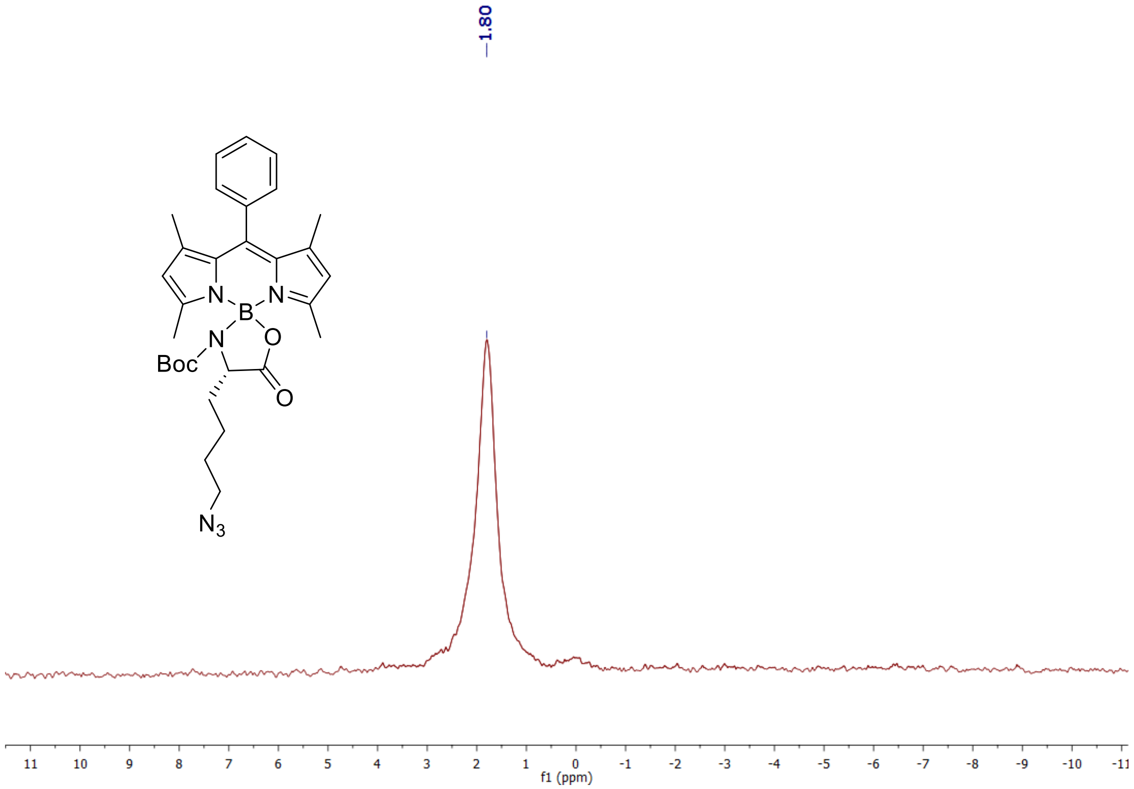

Figure S54. ^{11}B NMR (128 MHz, $CDCl_3$) spectrum of compound **8B**.

Compound 8C

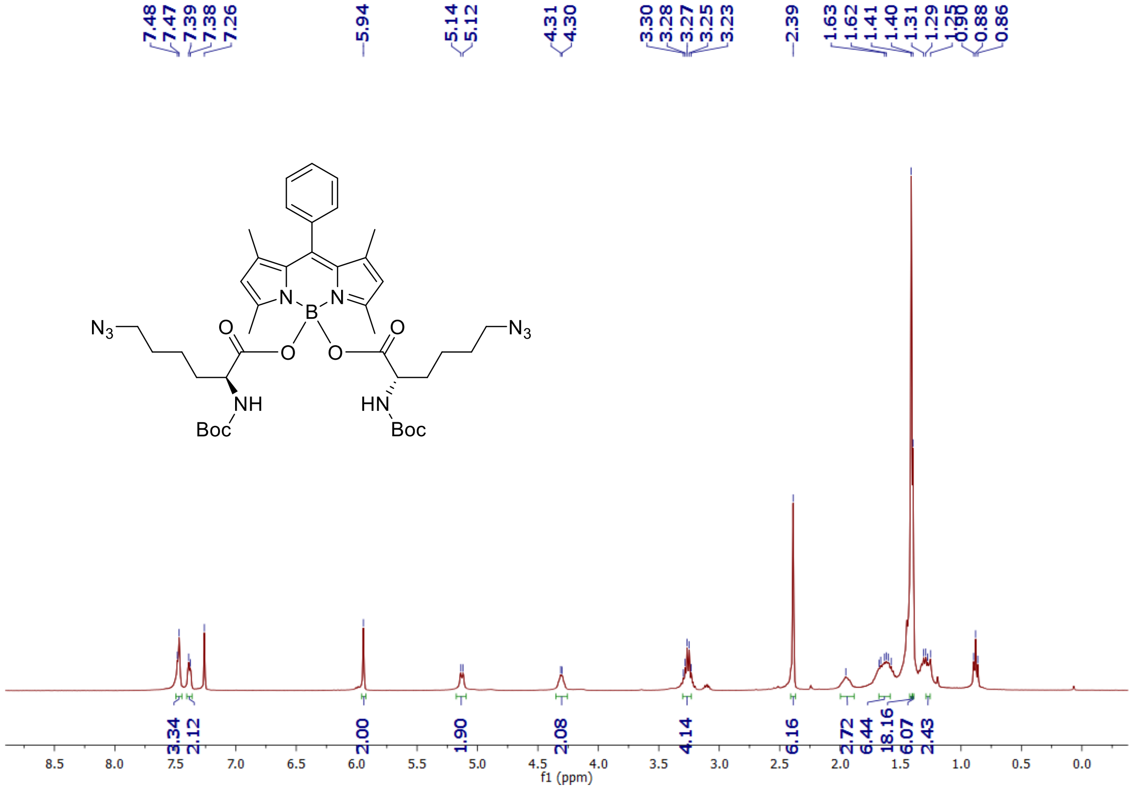

Figure S55. ^{1}H NMR (400 MHz, $CDCl_3$) spectrum of compound **8C**.

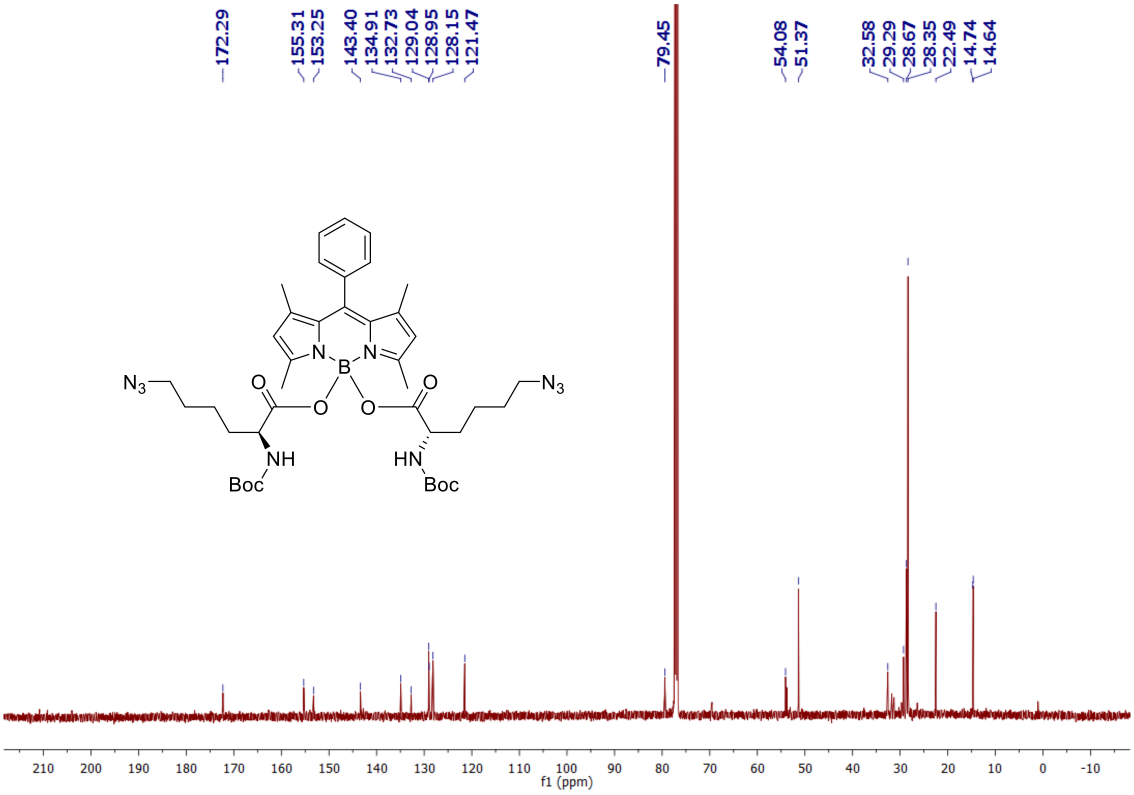

Figure S56. $^{13}C\{^{1}H\}$ NMR (100 MHz, $CDCl_3$) spectrum of compound **8C**.

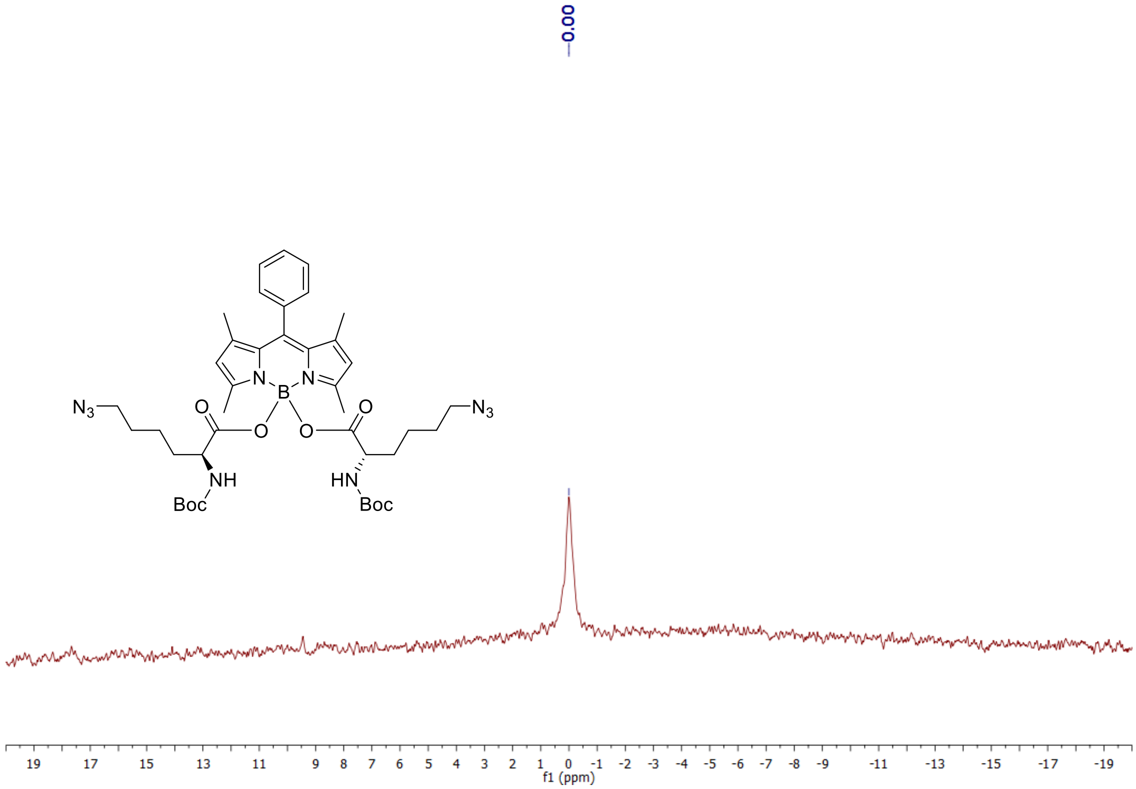

Figure S57. ^{11}B NMR (128 MHz, $CDCl_3$) spectrum of compound **8C**.

Compound 9B

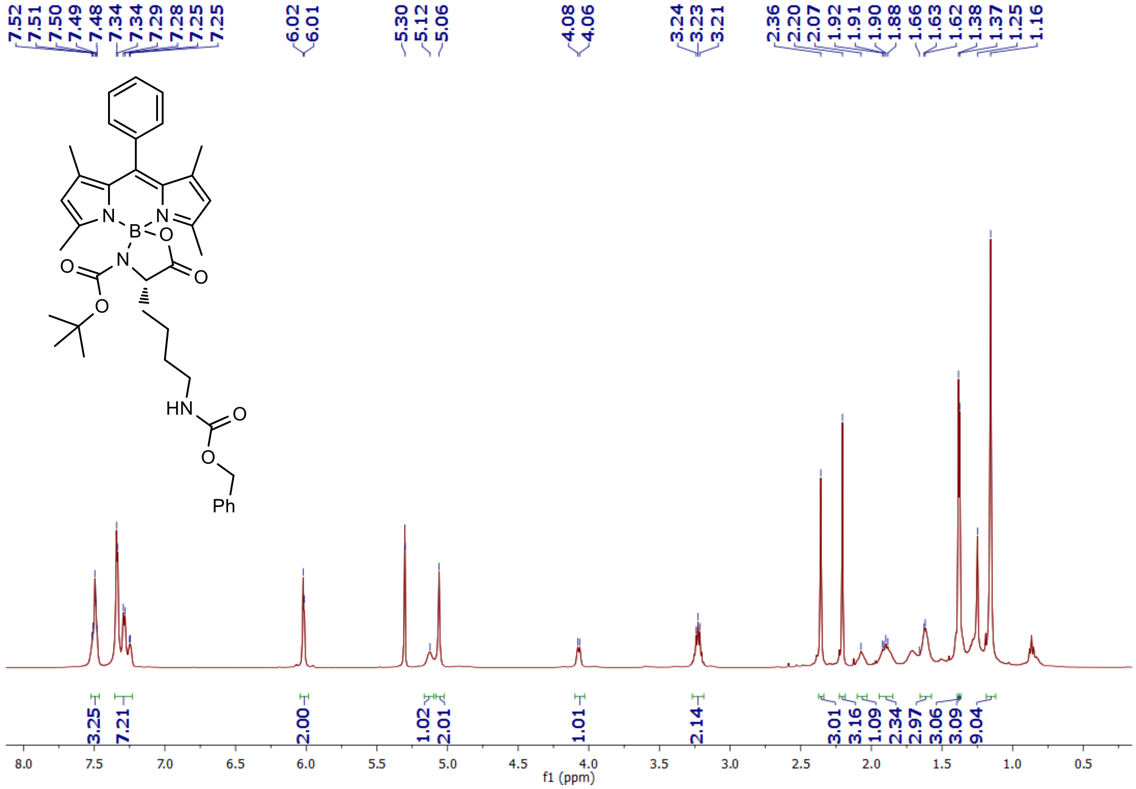

Figure S58. ^{1}H NMR (500 MHz, CD_2Cl_2) spectrum of compound **9B**.

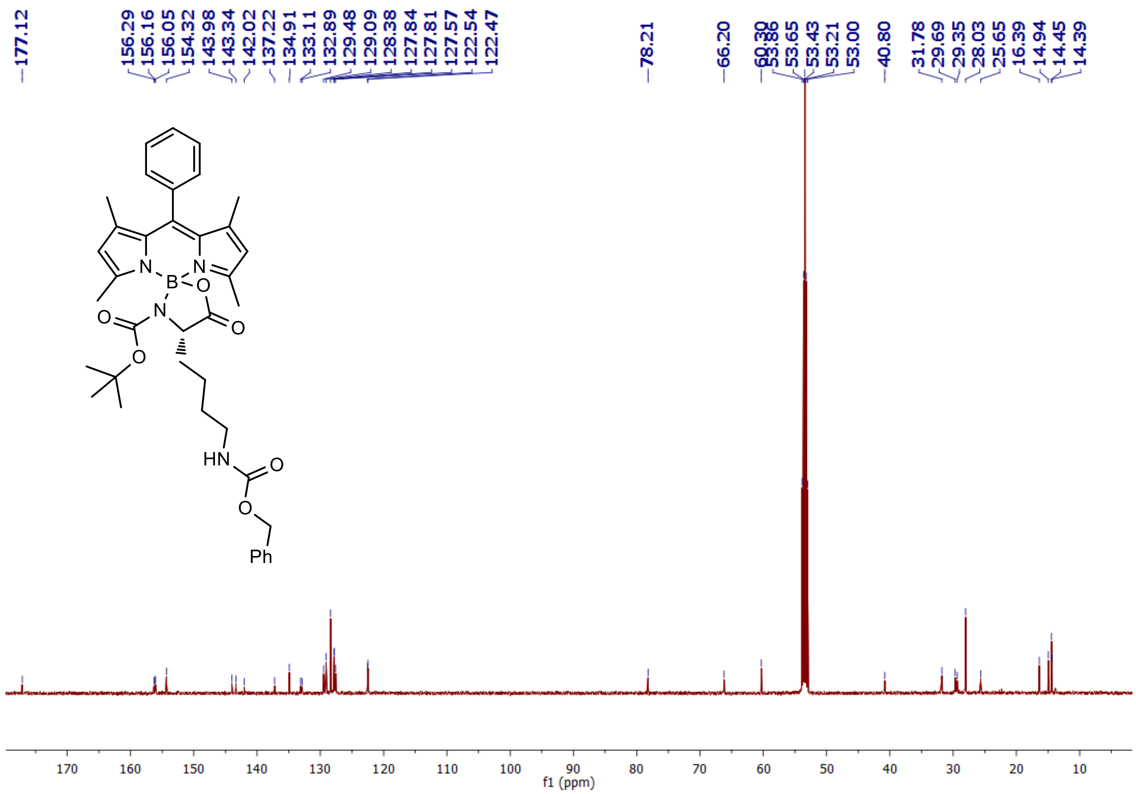

Figure S59. $^{13}C\{^{1}H\}$ NMR (126 MHz, CD_2Cl_2) spectrum of compound **9B**.

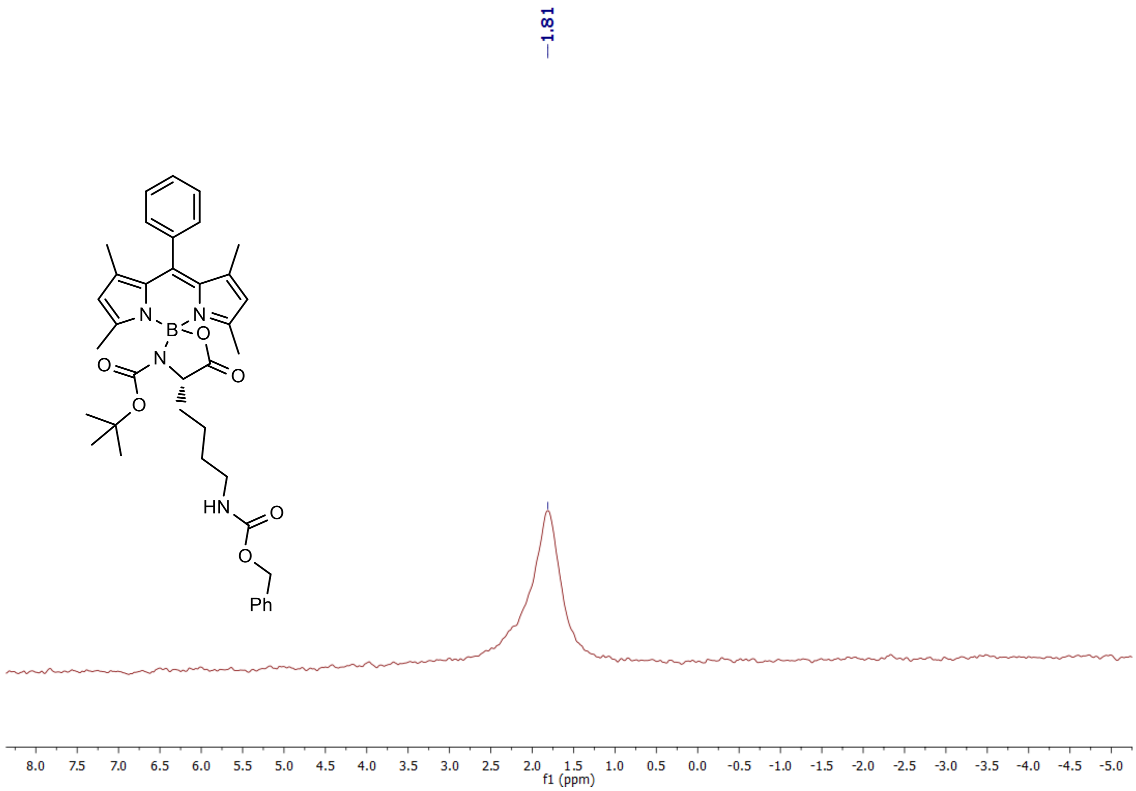

Figure S60. ^{11}B NMR (128 MHz, CD_2Cl_2) spectrum of compound **9B**.

Compound 9C

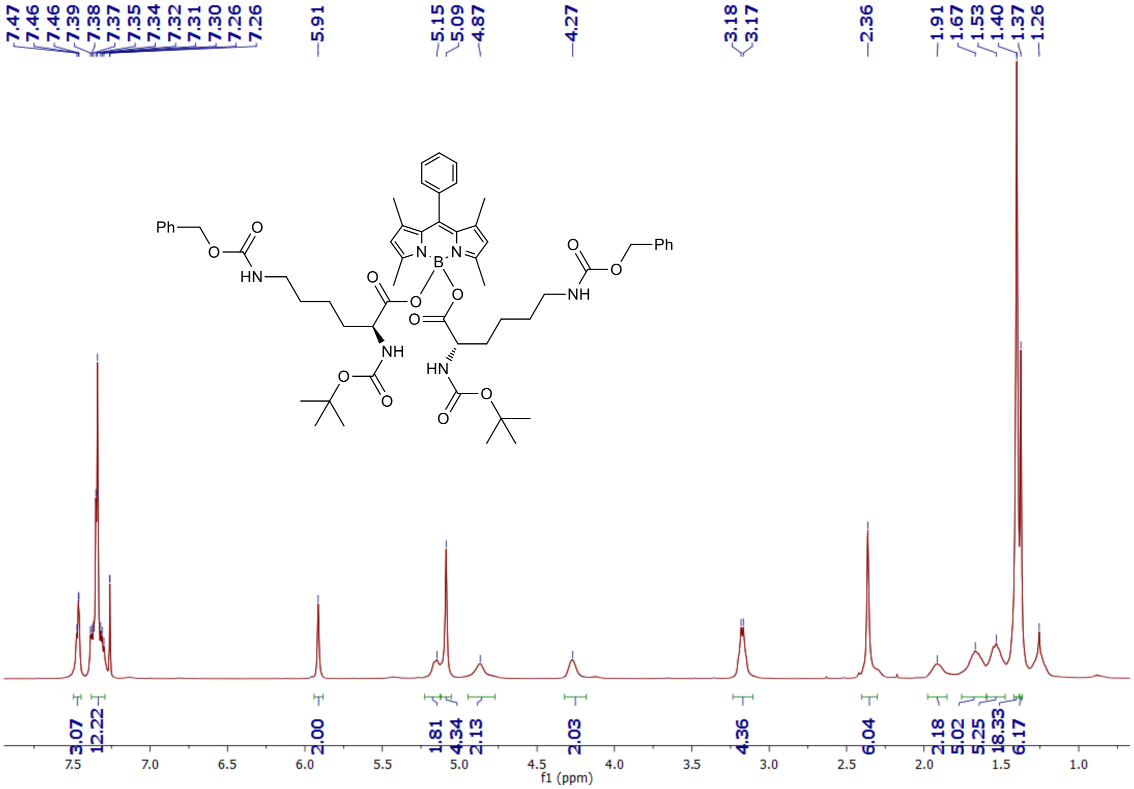

Figure S61. ^{1}H NMR (400 MHz, $CDCl_3$) spectrum of compound **9C**.

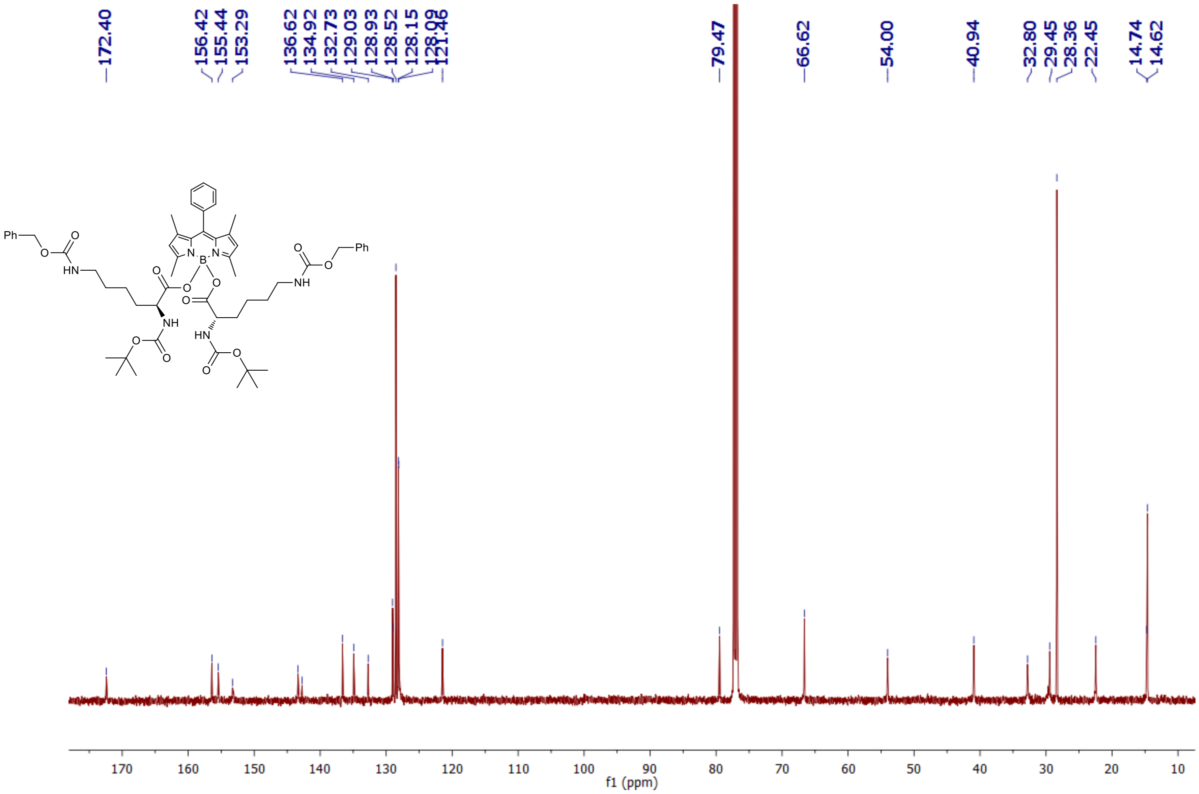

Figure S62. $^{13}C\{^{1}H\}$ NMR (100 MHz, $CDCl_3$) spectrum of compound **9C**.

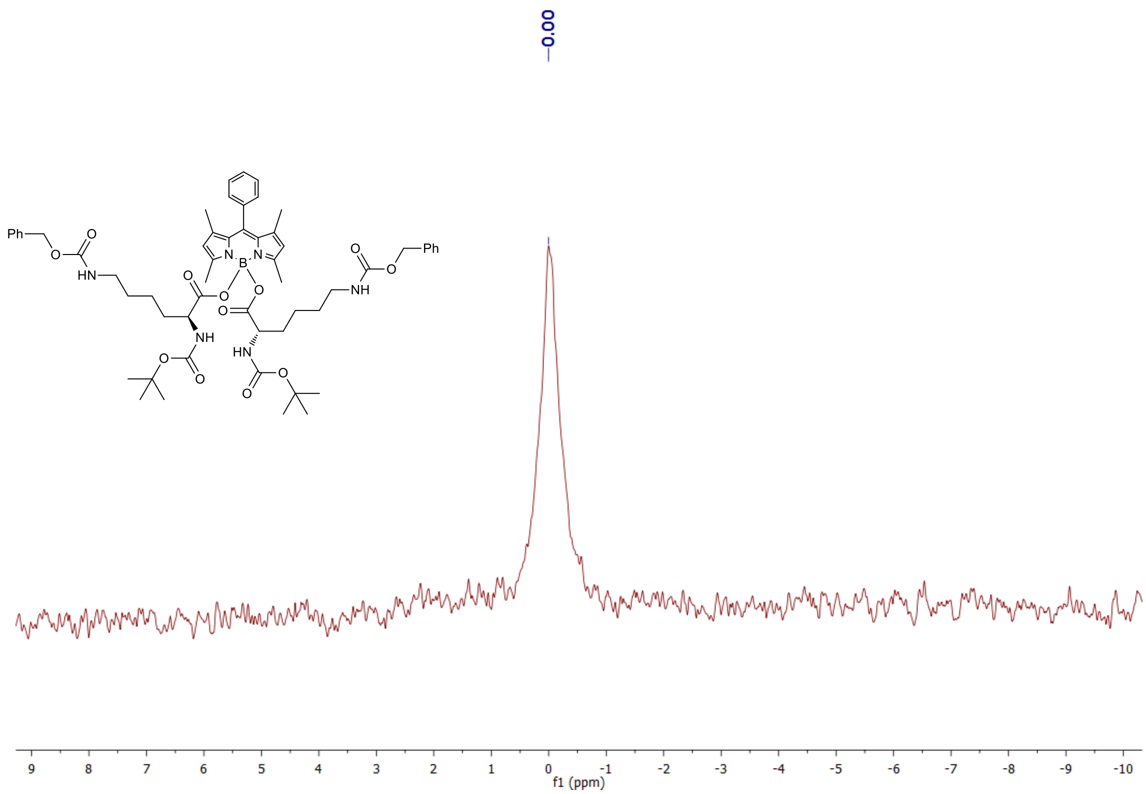

Figure S63. ^{11}B NMR (128 MHz, $CDCl_3$) spectrum of compound **9C**.

Compound 10A

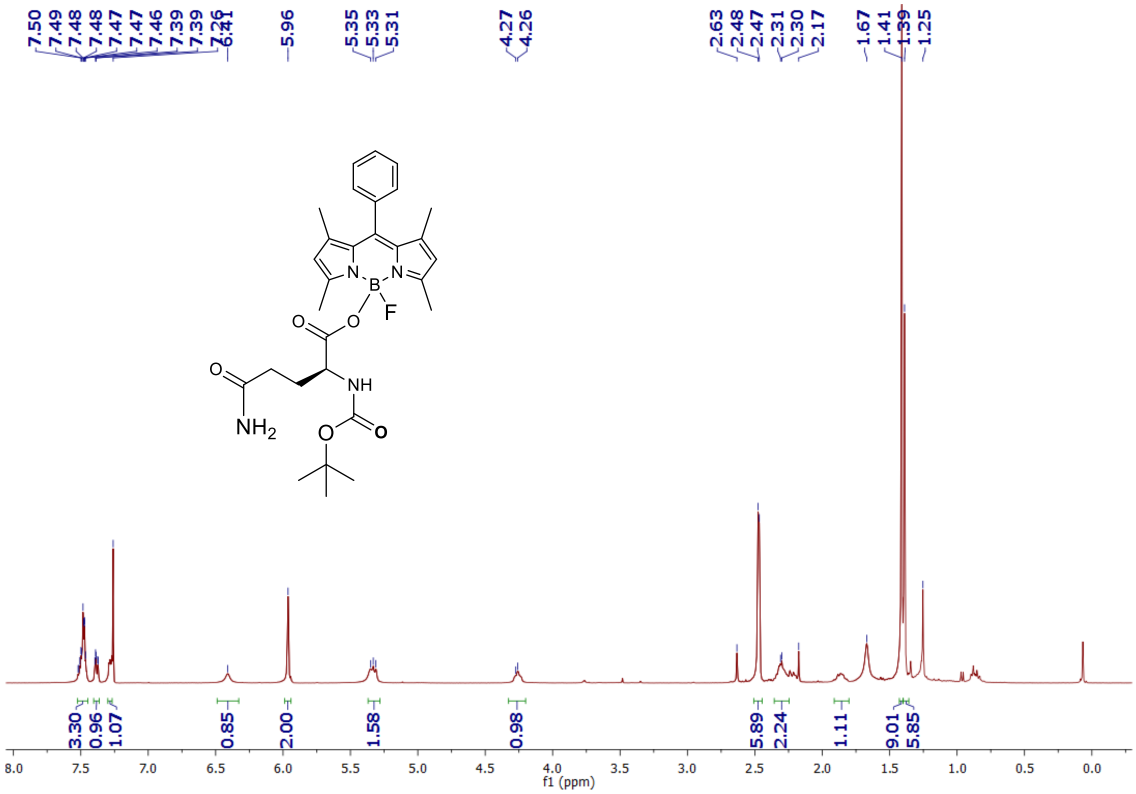

Figure S64. ^{1}H NMR (400 MHz, $CDCl_3$) spectrum of compound **10A**.

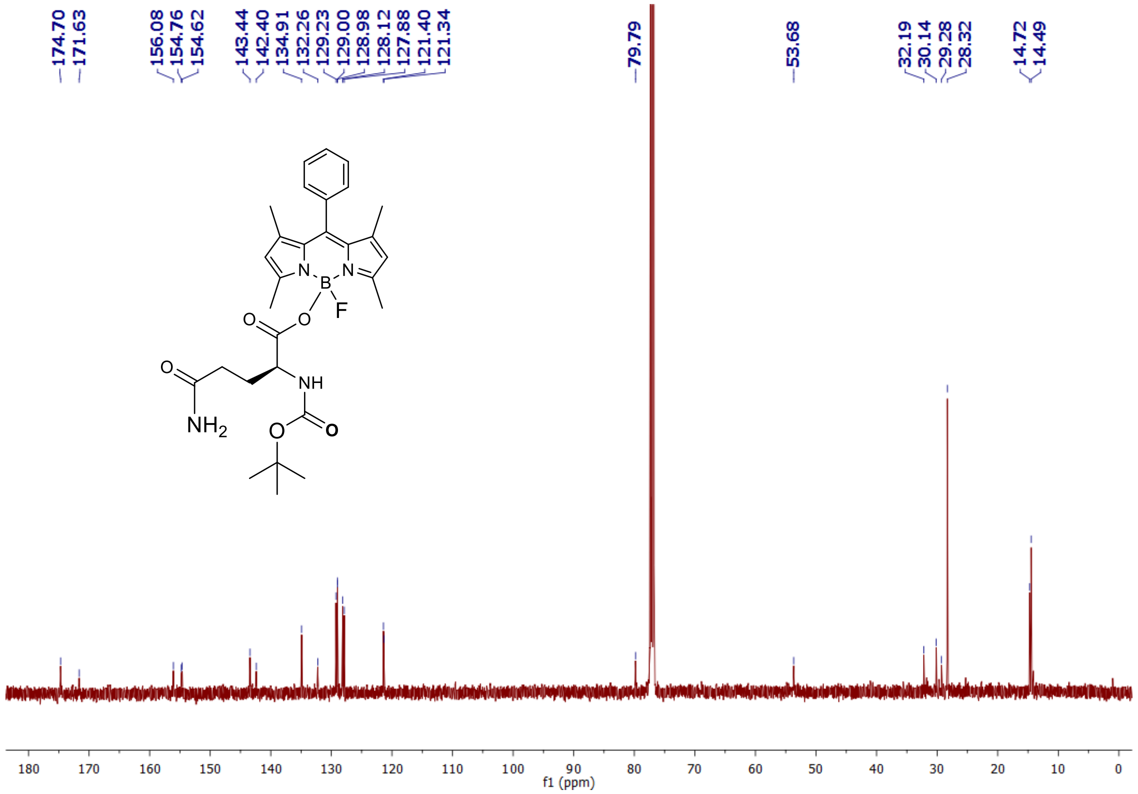

Figure S65. $^{13}C\{^{1}H\}$ NMR (100 MHz, $CDCl_3$) spectrum of compound **10A**.

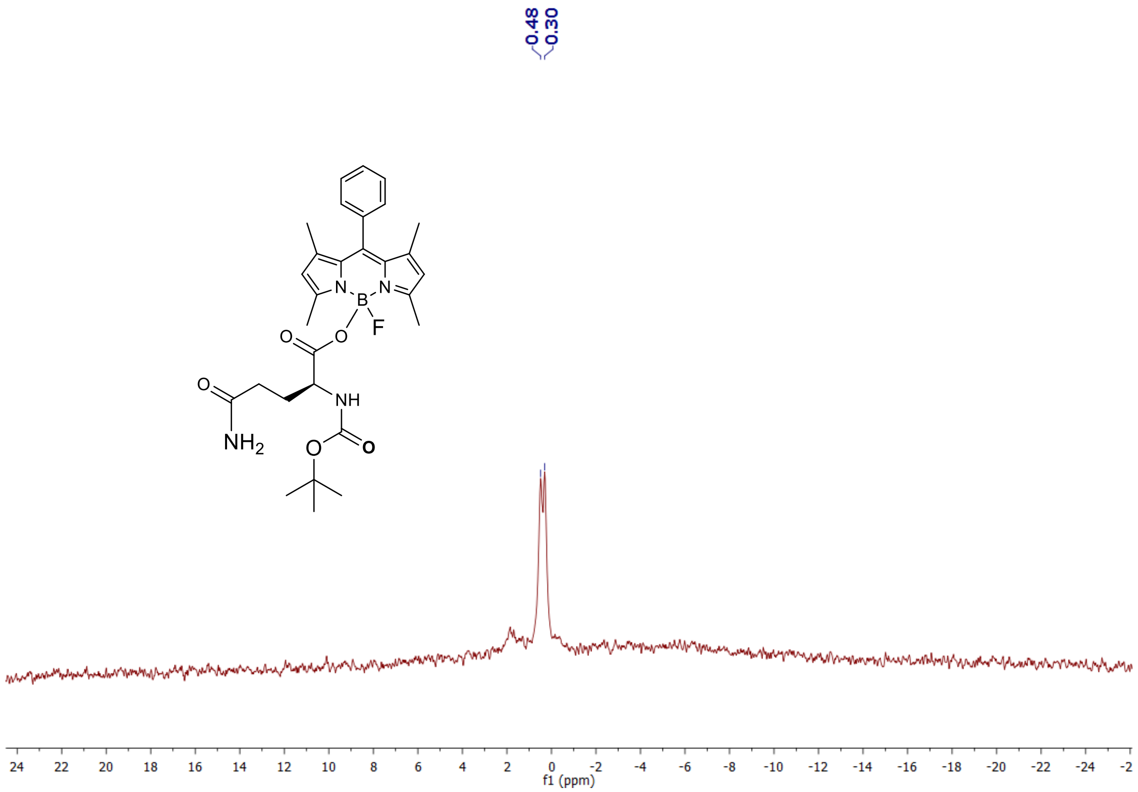

Figure S66. ^{11}B NMR (128 MHz, $CDCl_3$) spectrum of compound **10A**.

Compound 10B

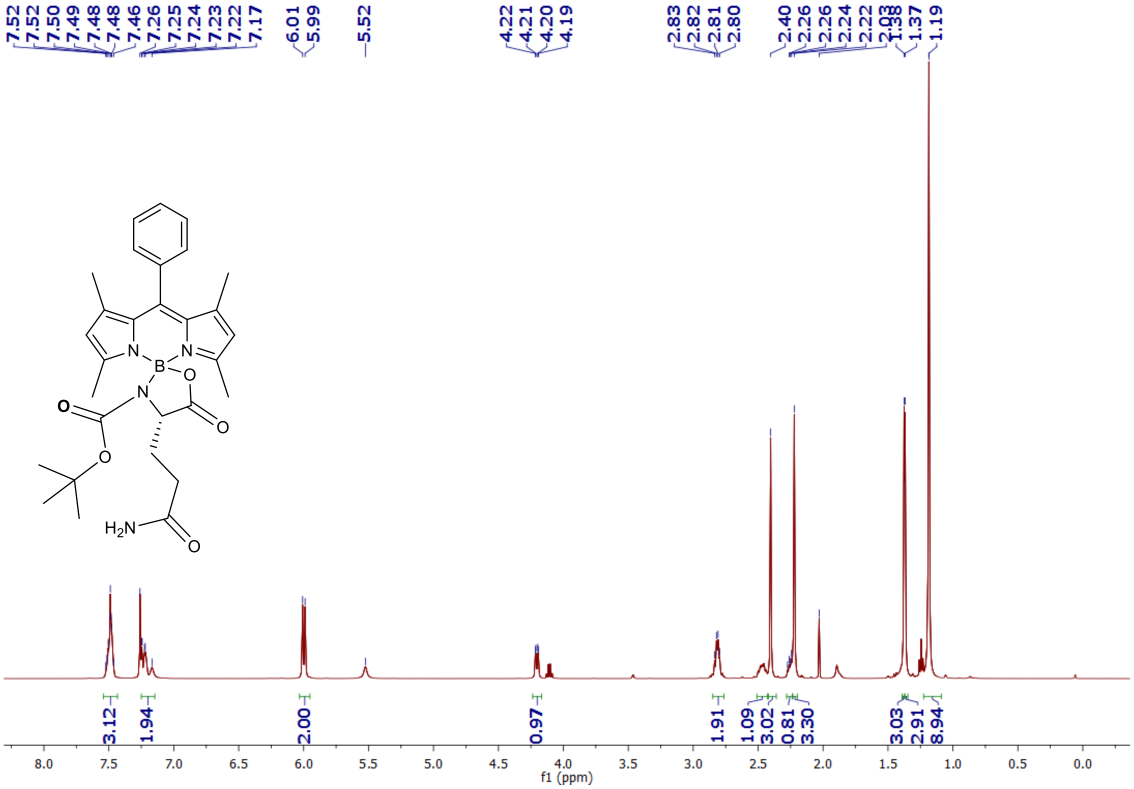

Figure S67. ^{1}H NMR (400 MHz, $CDCl_3$) spectrum of compound **10B**.

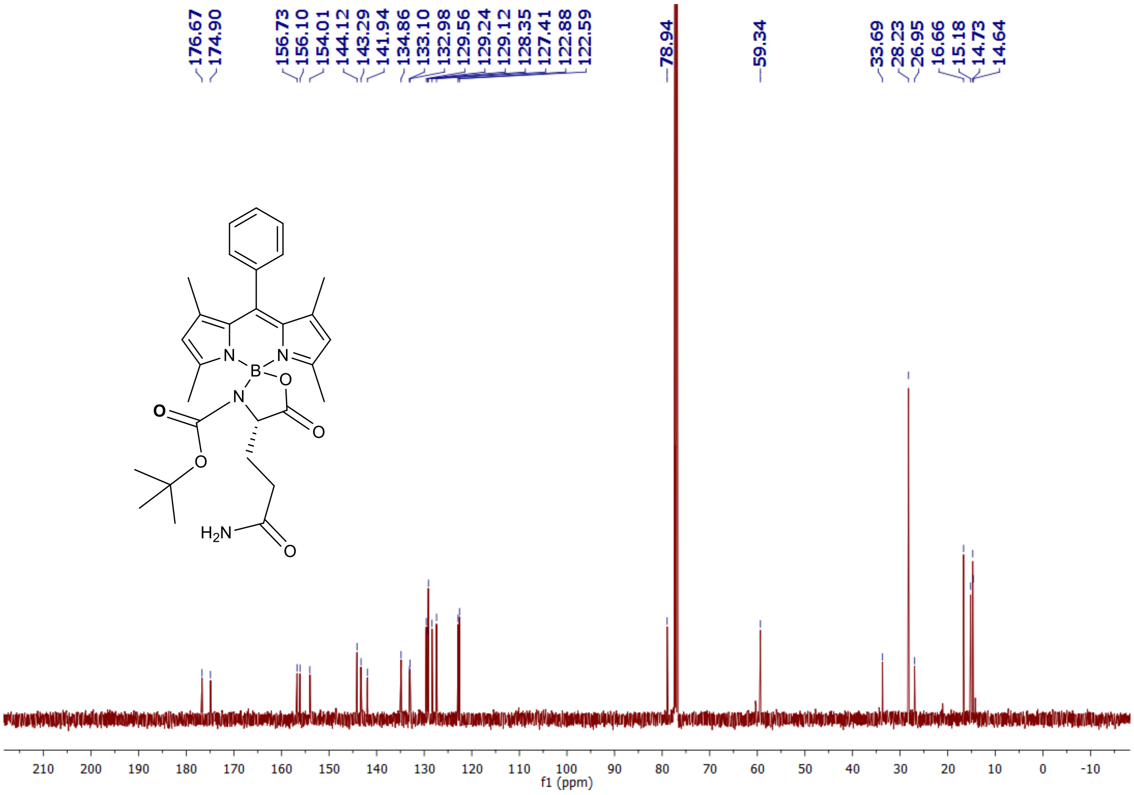

Figure S68. $^{13}C\{^{1}H\}$ NMR (100 MHz, $CDCl_3$) spectrum of compound **10B**.

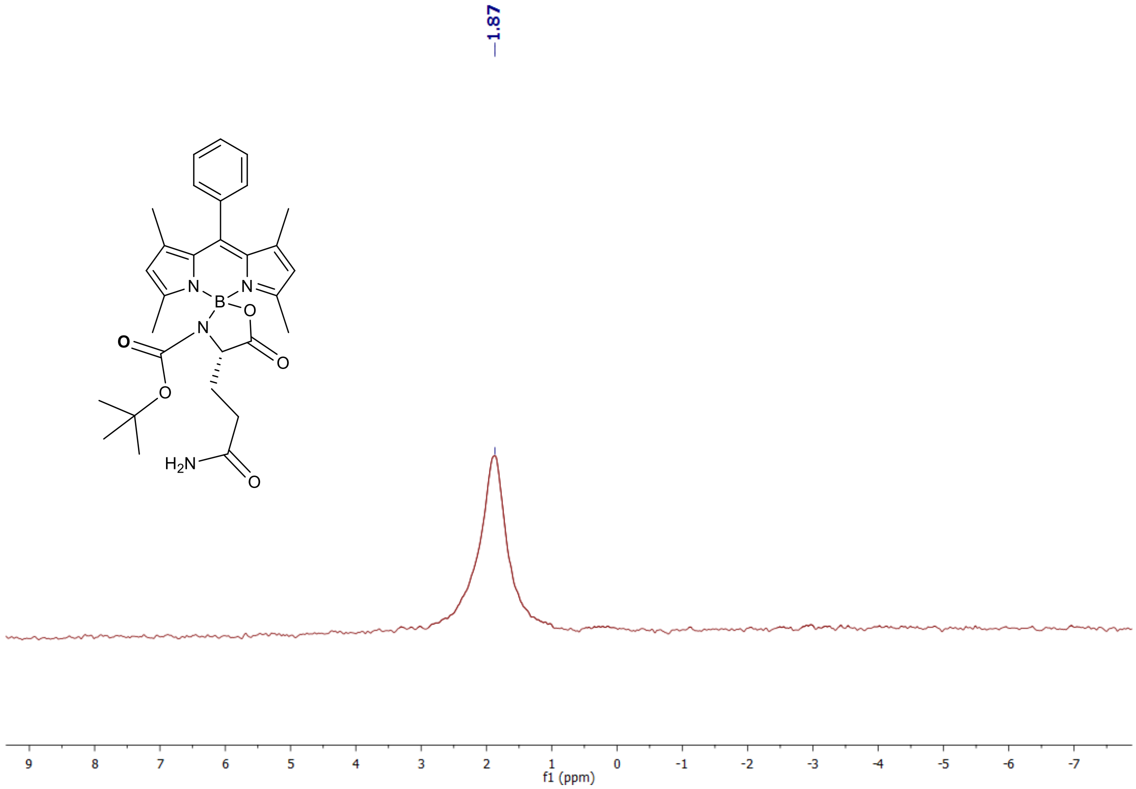

Figure S69. ^{11}B NMR (128 MHz, CD_3OD) spectrum of compound **10B**.

Compound 10C

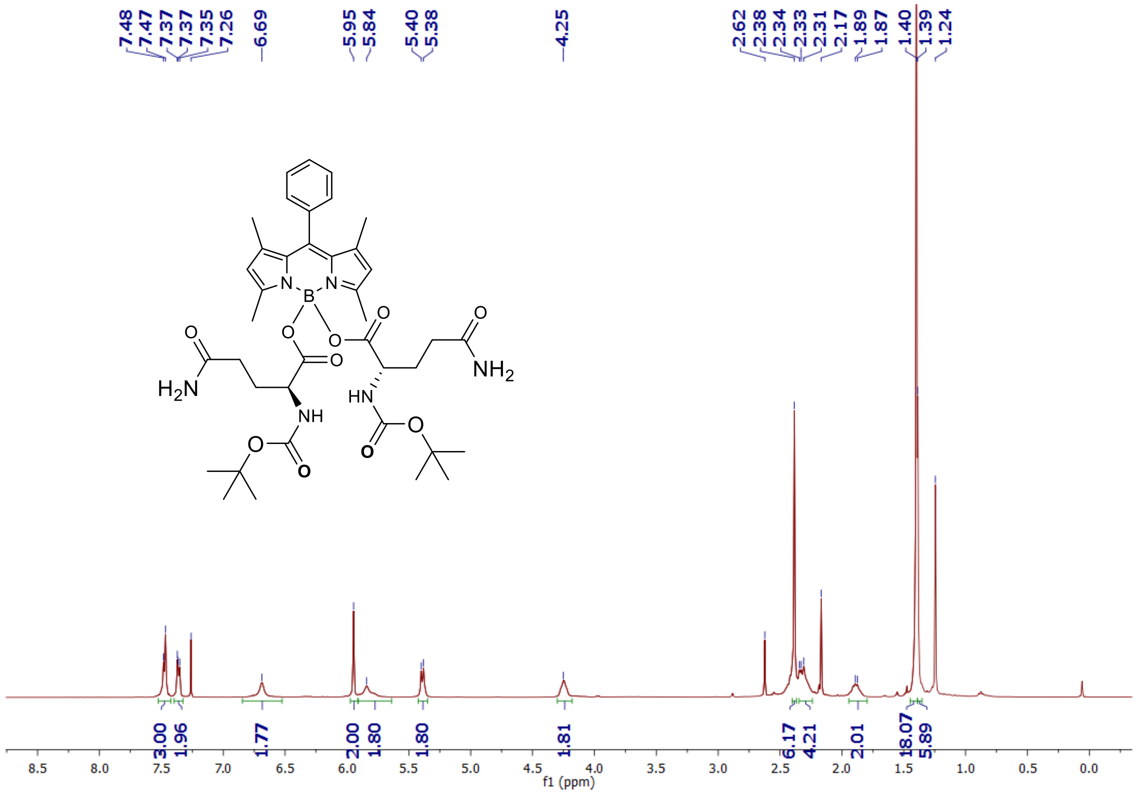

Figure S70. ^{1}H NMR (400 MHz, $CDCl_3$) spectrum of compound **10C**.

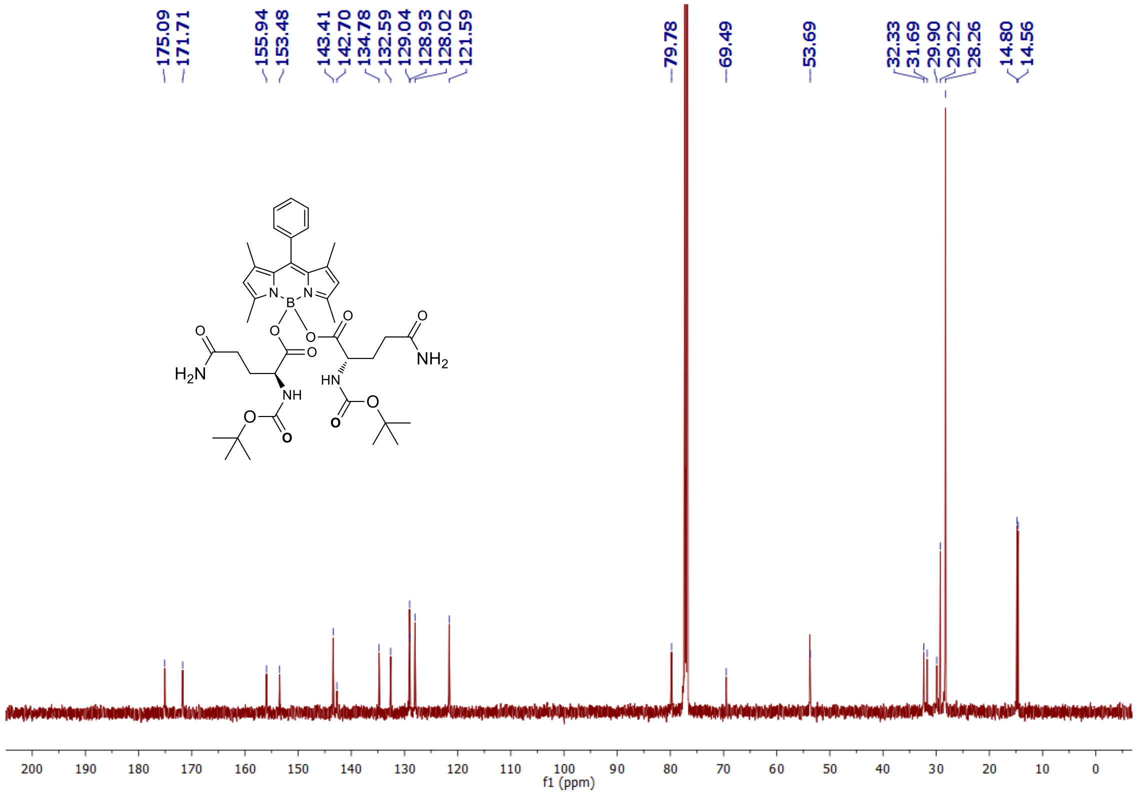

Figure S71. $^{13}C\{^{1}H\}$ NMR (100 MHz, $CDCl_3$) spectrum of compound **10C**.

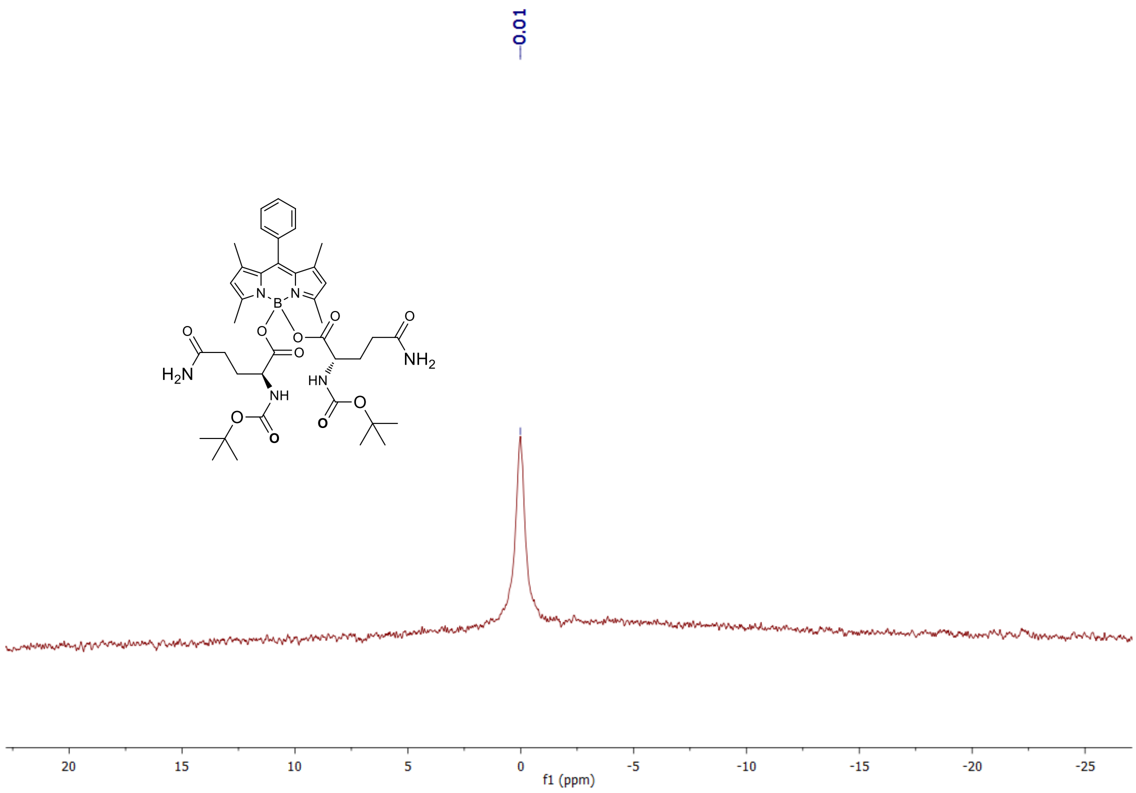

Figure S72. ^{11}B NMR (128 MHz, $CDCl_3$) spectrum of compound **10C**.

Compound 11B

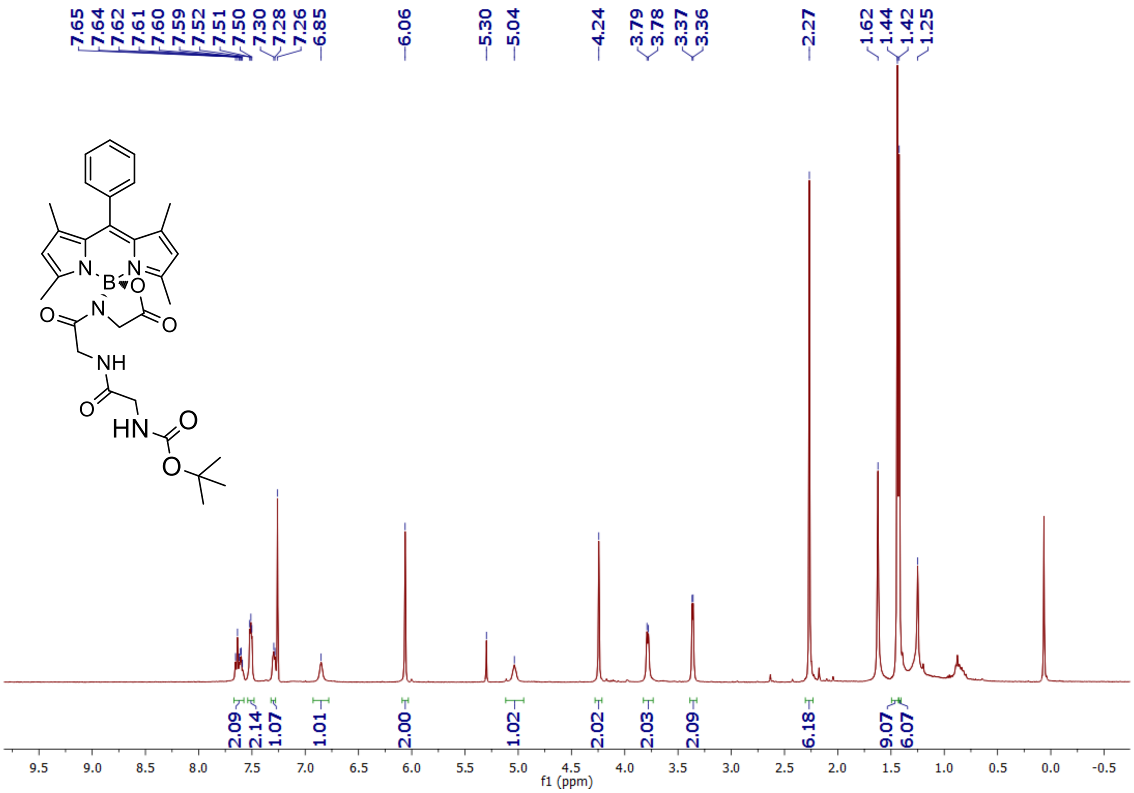

Figure S73. ^{1}H NMR (400 MHz, $CDCl_3$) spectrum of compound **11B**.

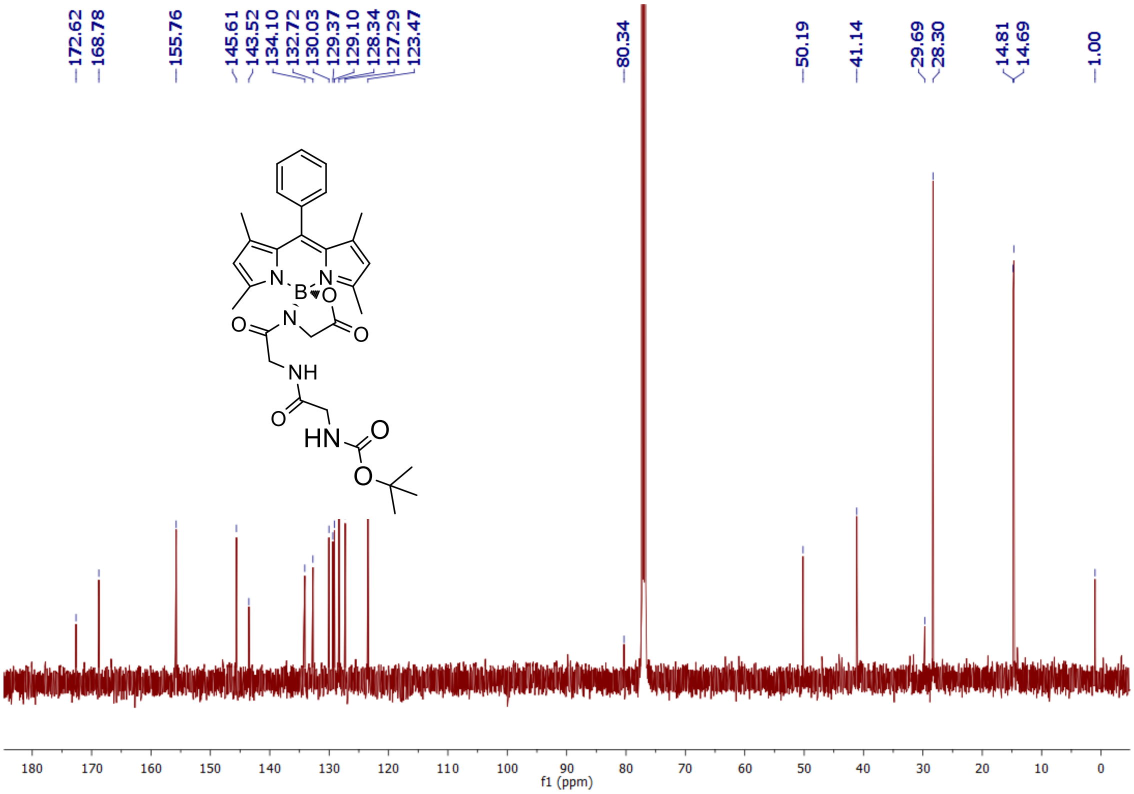

Figure S74. $^{13}C\{^{1}H\}$ NMR (100 MHz, $CDCl_3$) spectrum of compound **11B**.

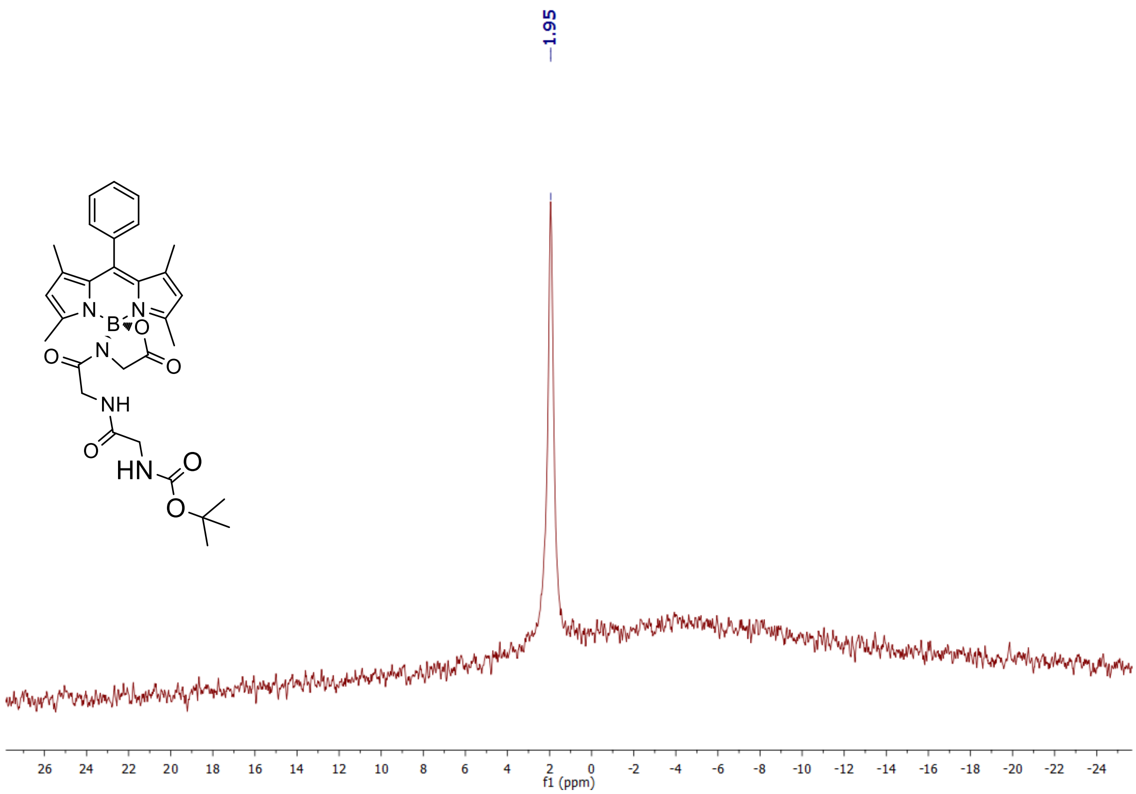

Figure S75. ^{11}B NMR (128 MHz, $CDCl_3$) spectrum of compound **11B**.

Compound 11C

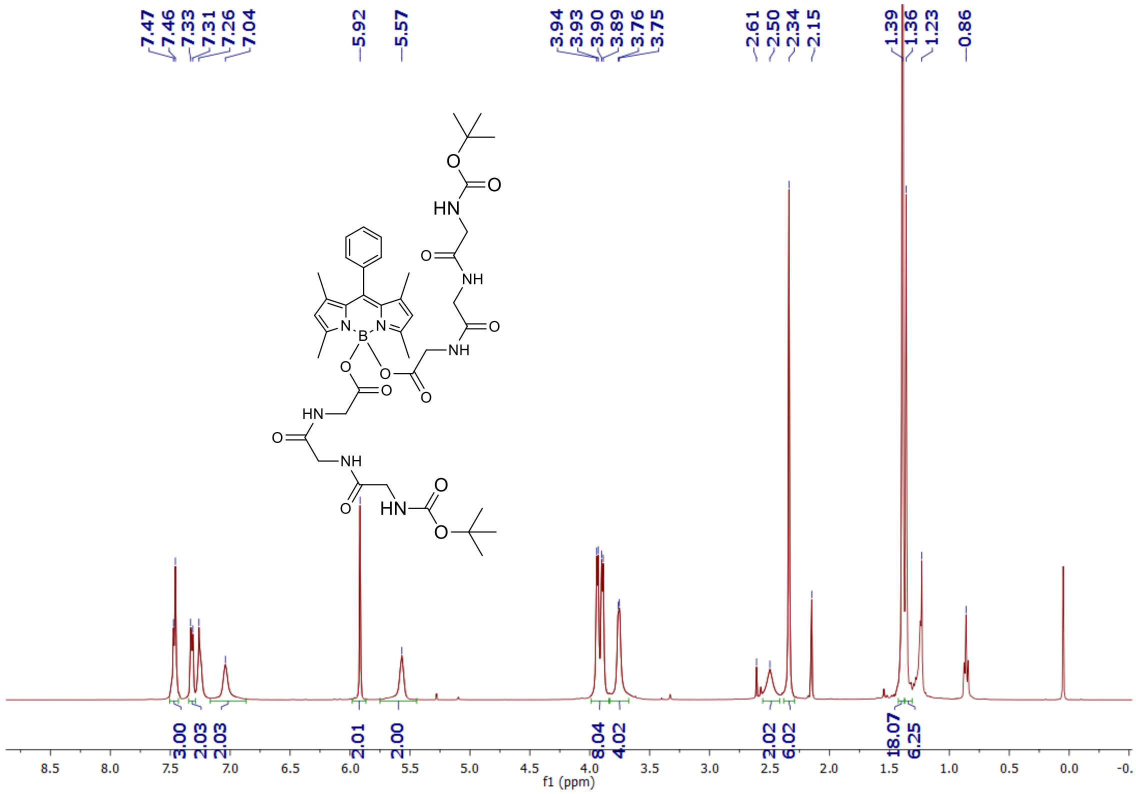

Figure S76. ^{1}H NMR (400 MHz, $CDCl_3$) spectrum of compound **11C**.

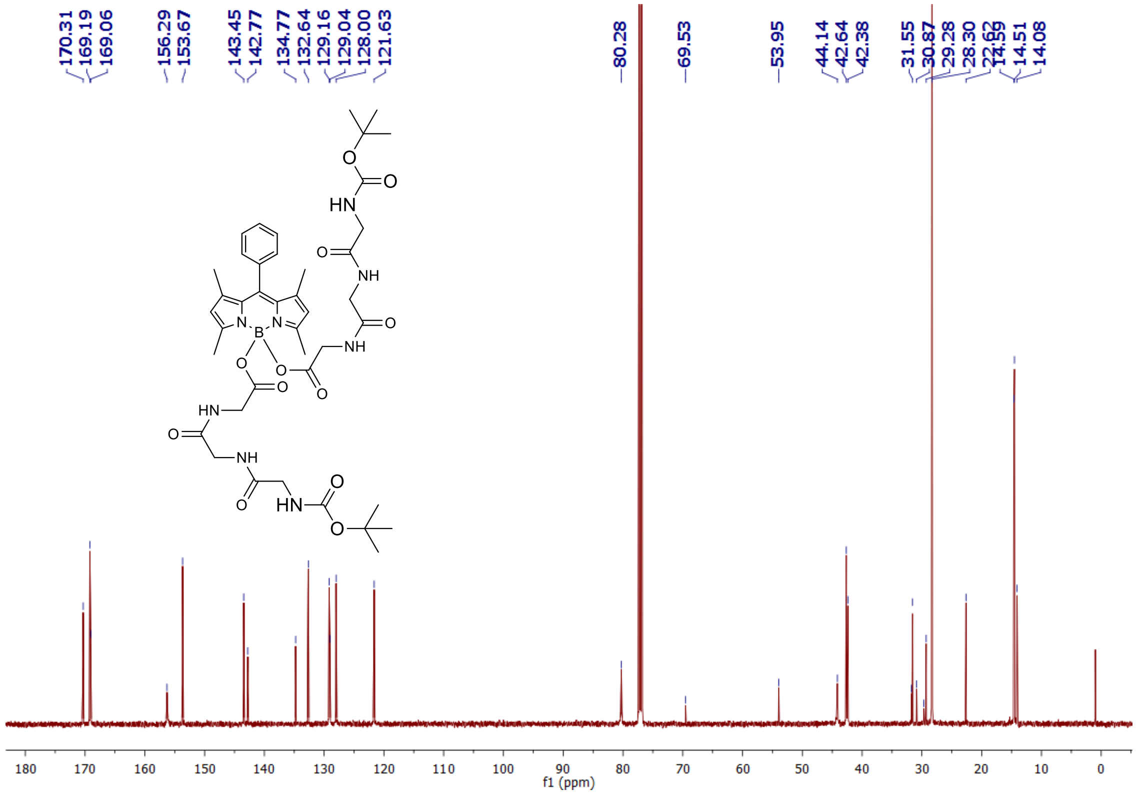

Figure S77. $^{13}C\{^{1}H\}$ NMR (100 MHz, $CDCl_3$) spectrum of compound **11C**.

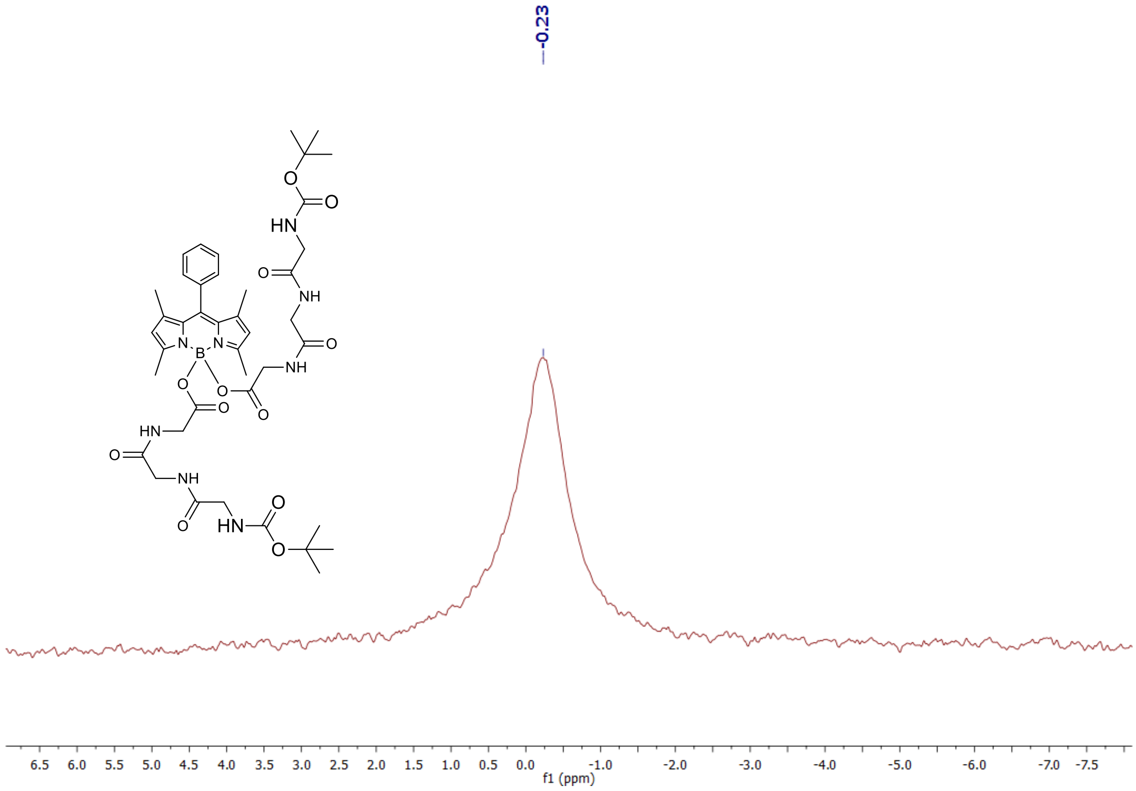

Figure S78. ^{11}B NMR (128 MHz, $CDCl_3$) spectrum of compound **11C**.

Compound 2b

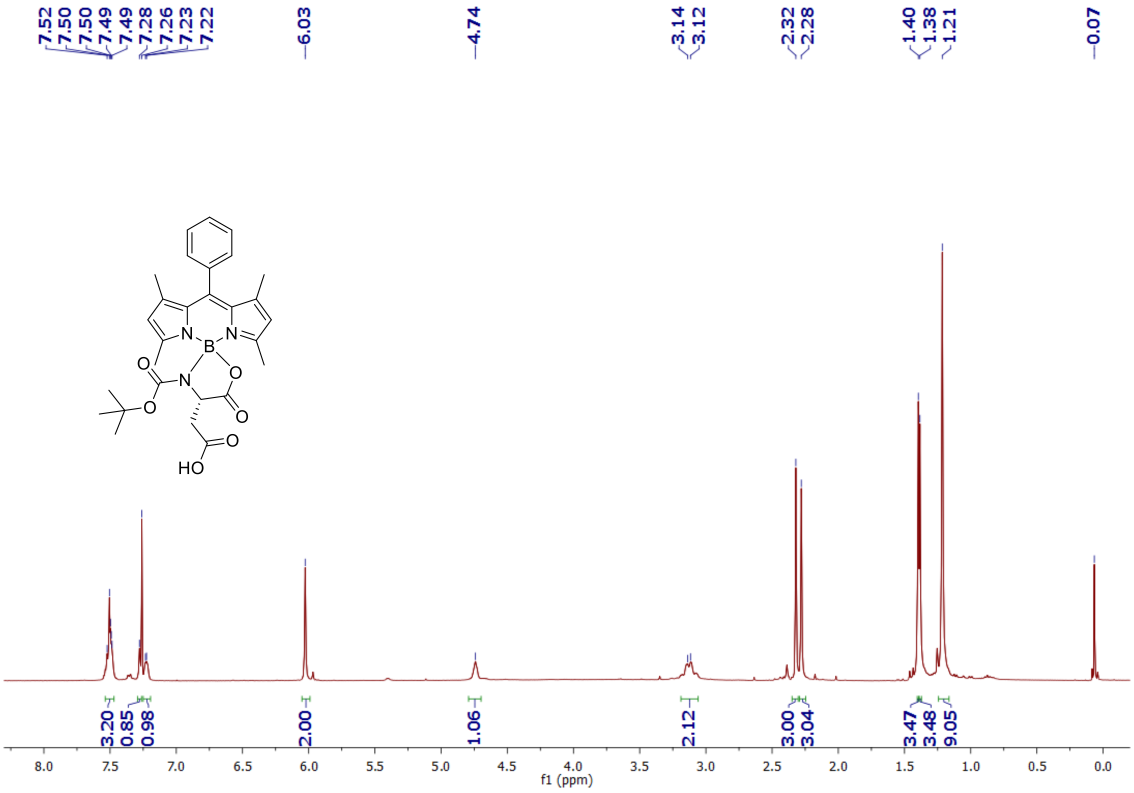

Figure S79. ^{1}H NMR (400 MHz, $CDCl_3$) spectrum of compound **2b**.

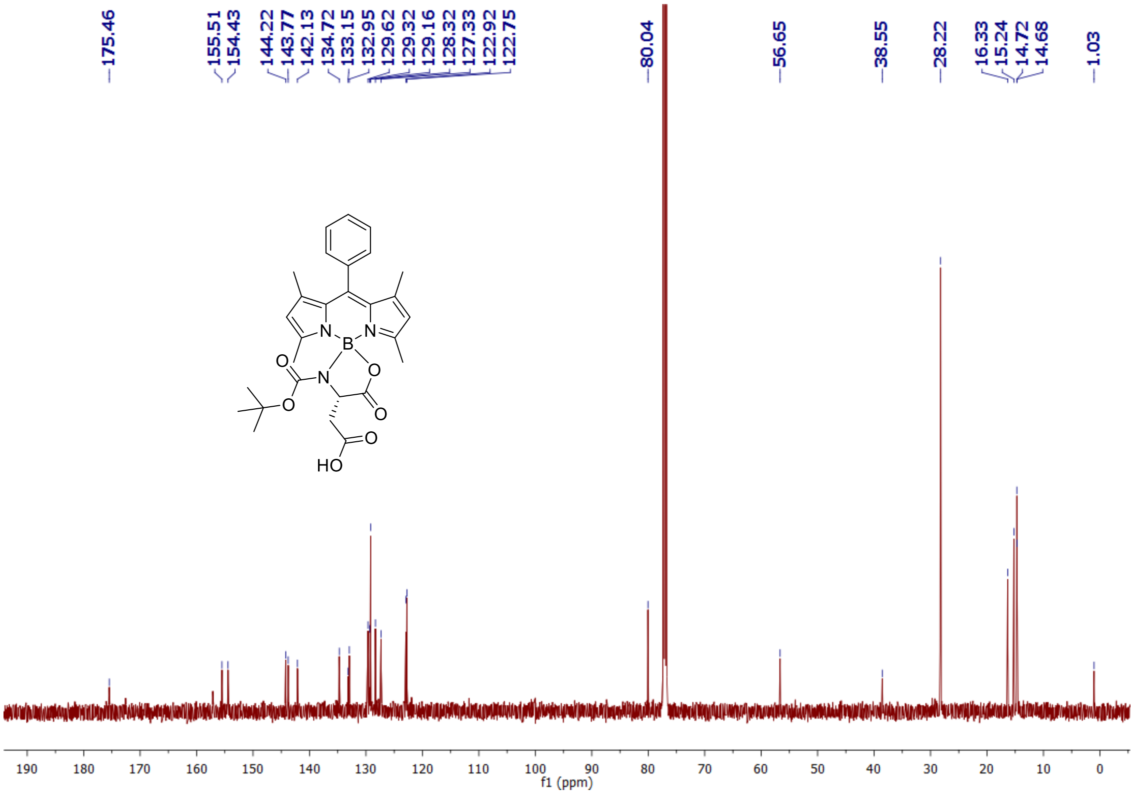

Figure S80. ^{13}C{^{1}H} NMR (100 MHz, $CDCl_3$) spectrum of compound **2b**.

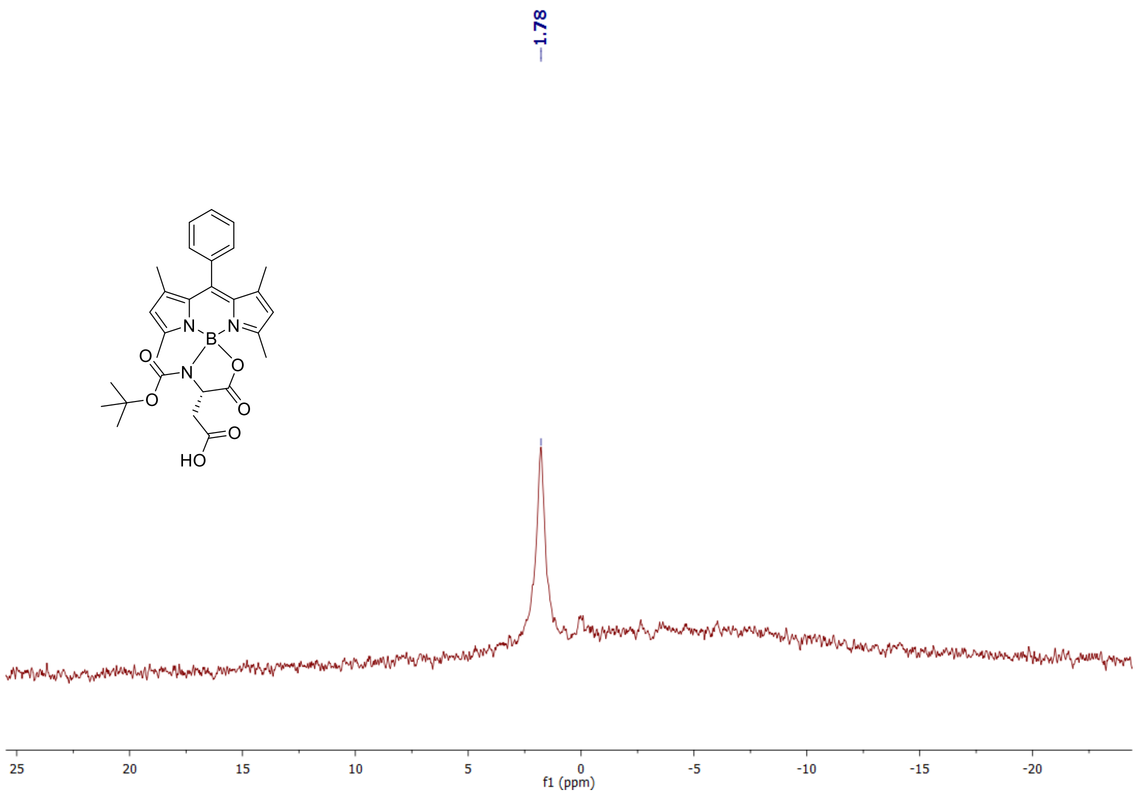

Figure S81. ^{11}B NMR (128 MHz, $CDCl_3$) spectrum of compound **2b**.

Compound 2c

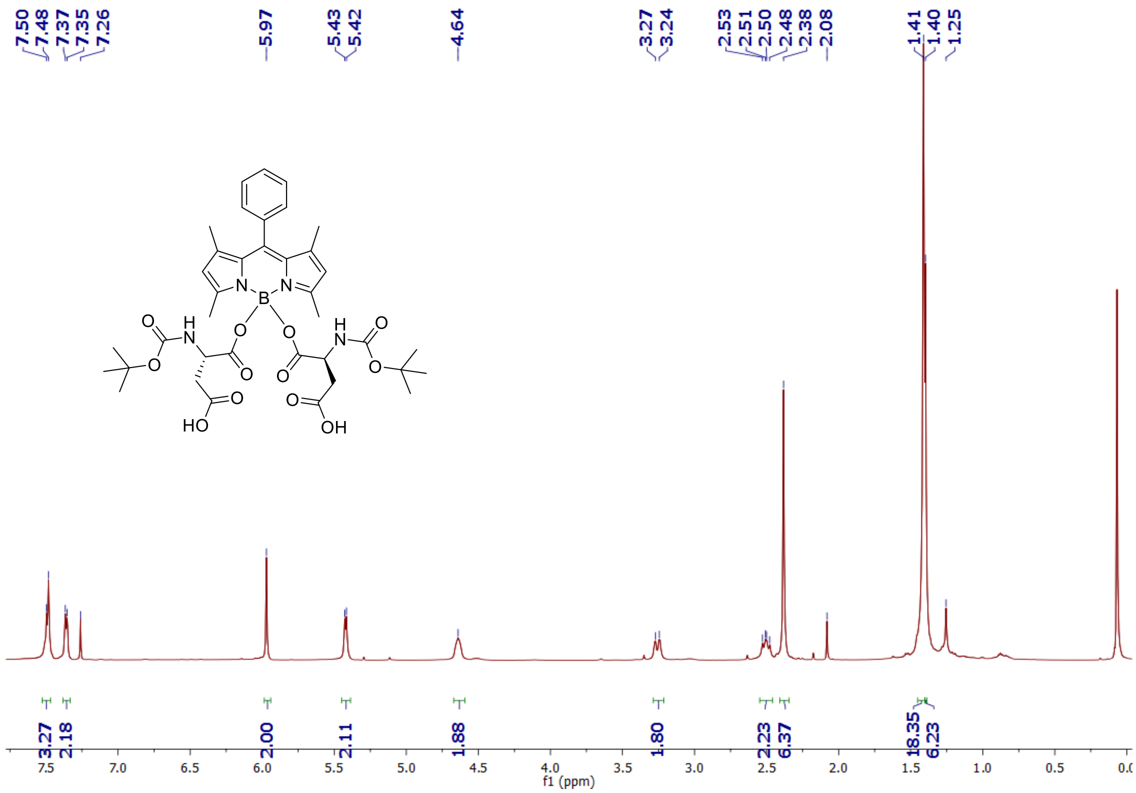

Figure S82. ^{1}H NMR (400 MHz, $CDCl_3$) spectrum of compound **2c**.

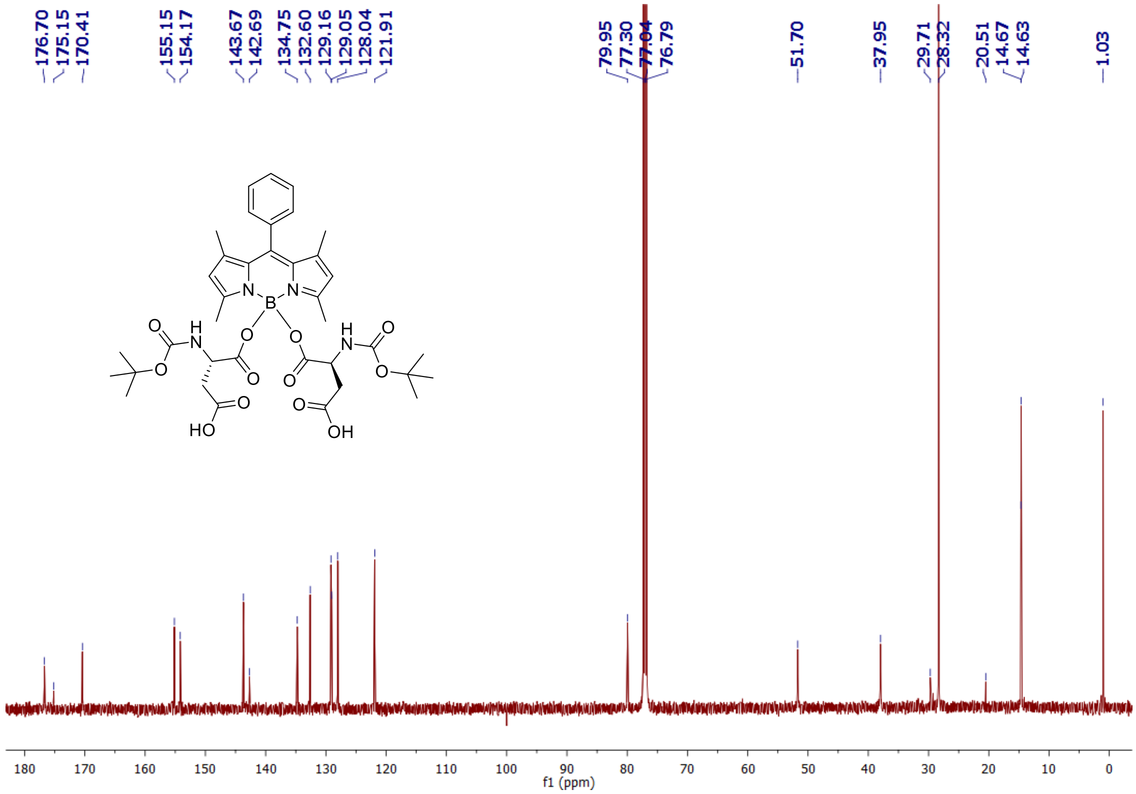

Figure S83. $^{13}C\{^{1}H\}$ NMR (100 MHz, $CDCl_3$) spectrum of compound **2c**.

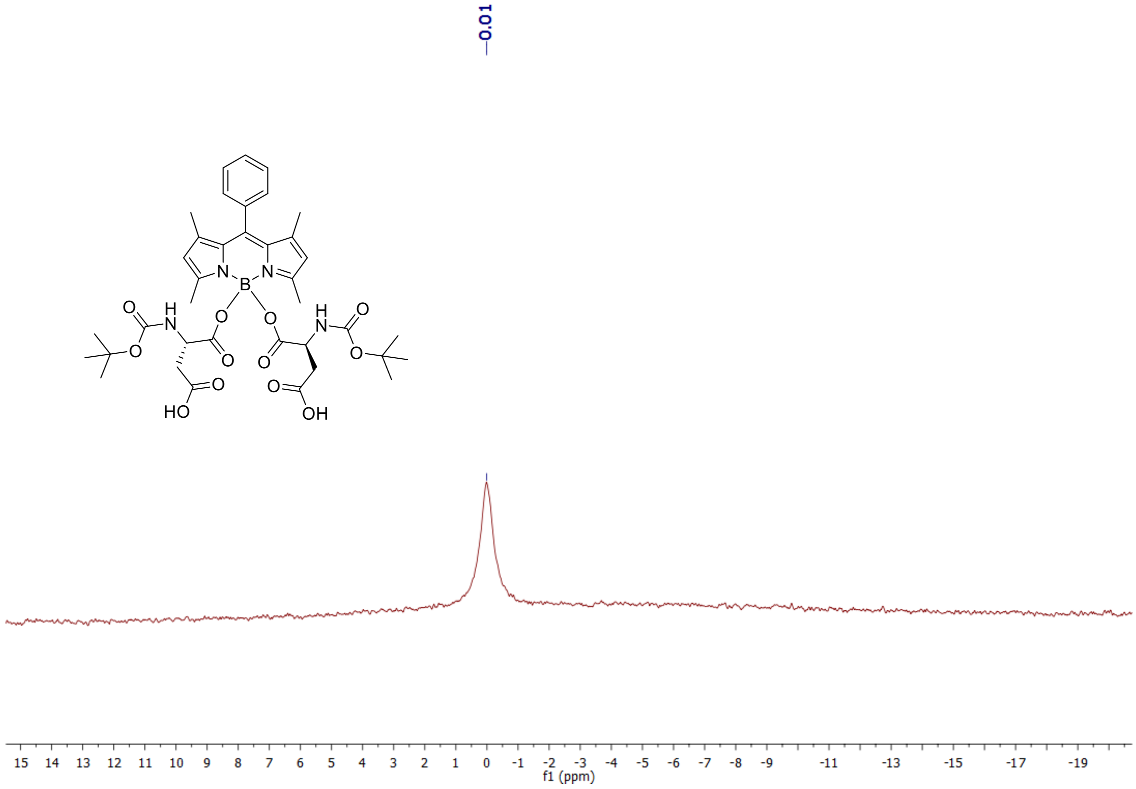

Figure S84. ^{11}B NMR (128 MHz, $CDCl_3$) spectrum of compound **2c**.

Compound 3b

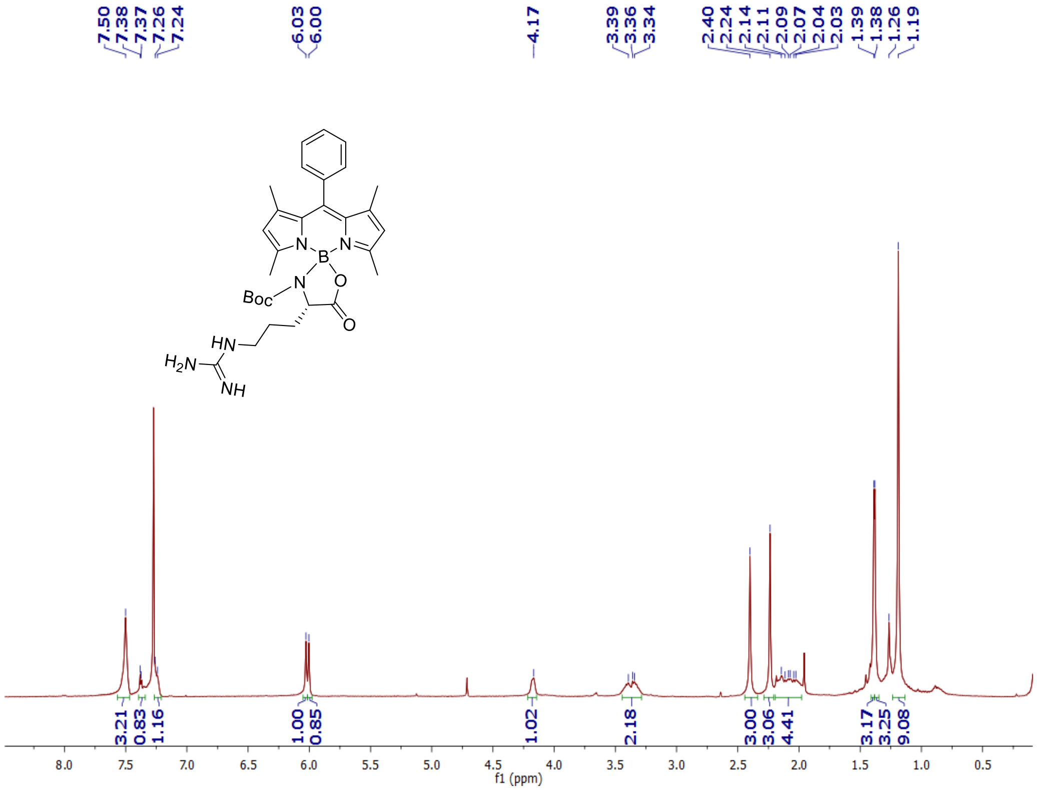

Figure S85. ^{1}H NMR (400 MHz, $CDCl_3$) spectrum of compound **3b**.

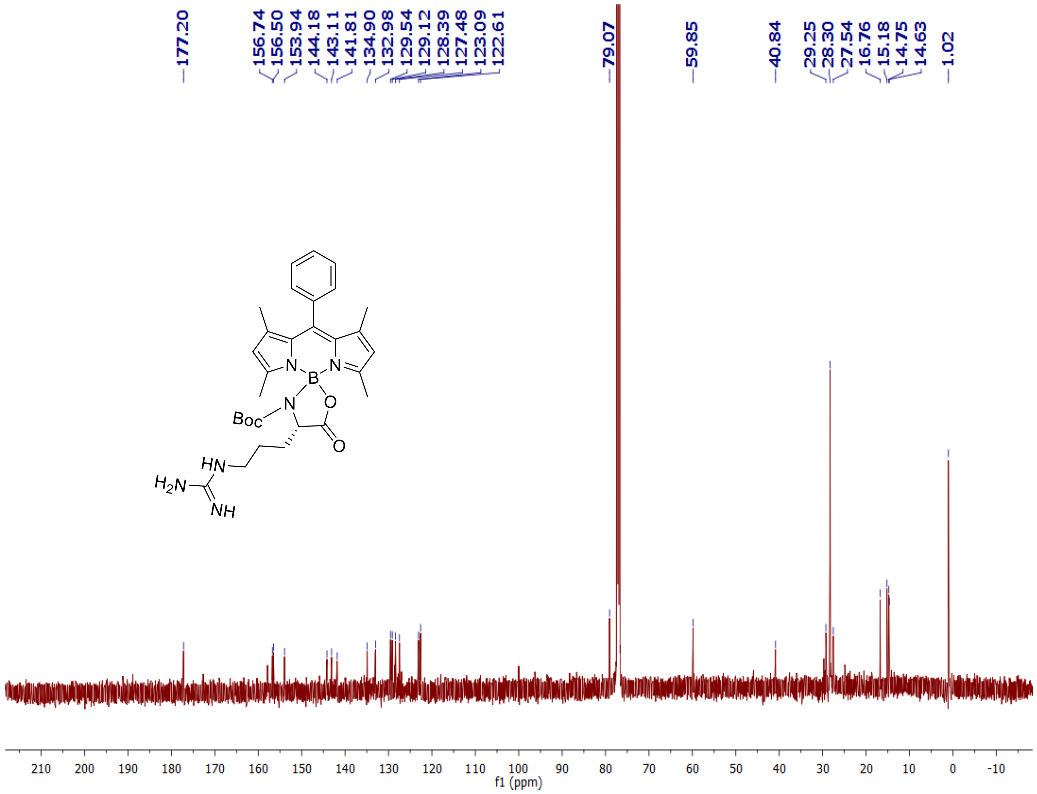

Figure S86. $^{13}C\{^{1}H\}$ NMR (100 MHz, $CDCl_3$) spectrum of compound **3b**.

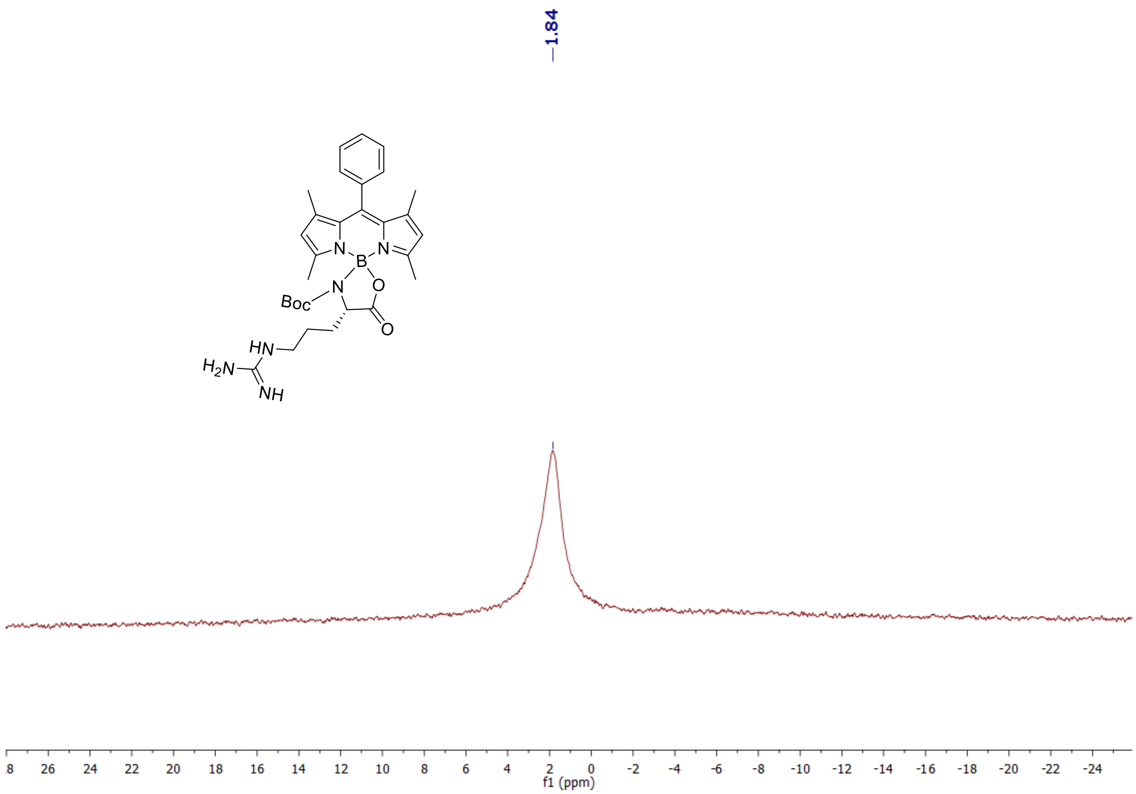

Figure S87. ^{11}B NMR (128 MHz, $CDCl_3$) spectrum of compound **3b**.

Compound 3c

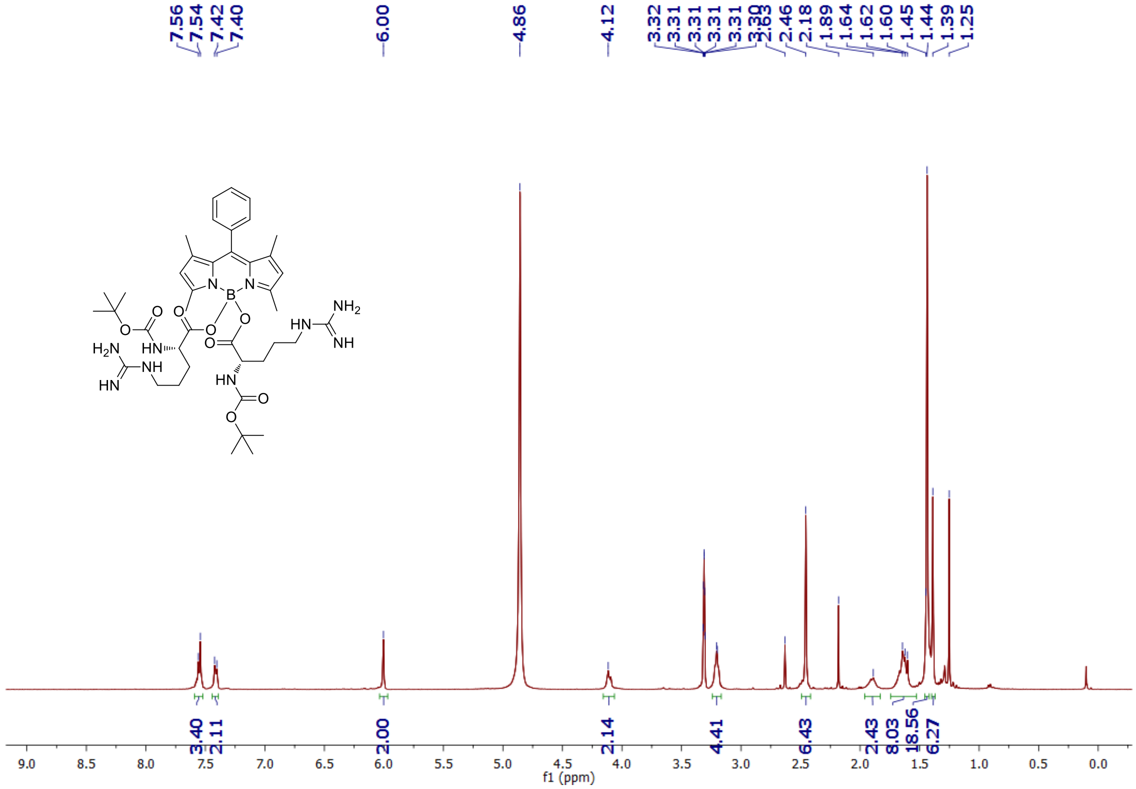

Figure S88. ^{1}H NMR (400 MHz, CD_3OD) spectrum of compound **3c**.

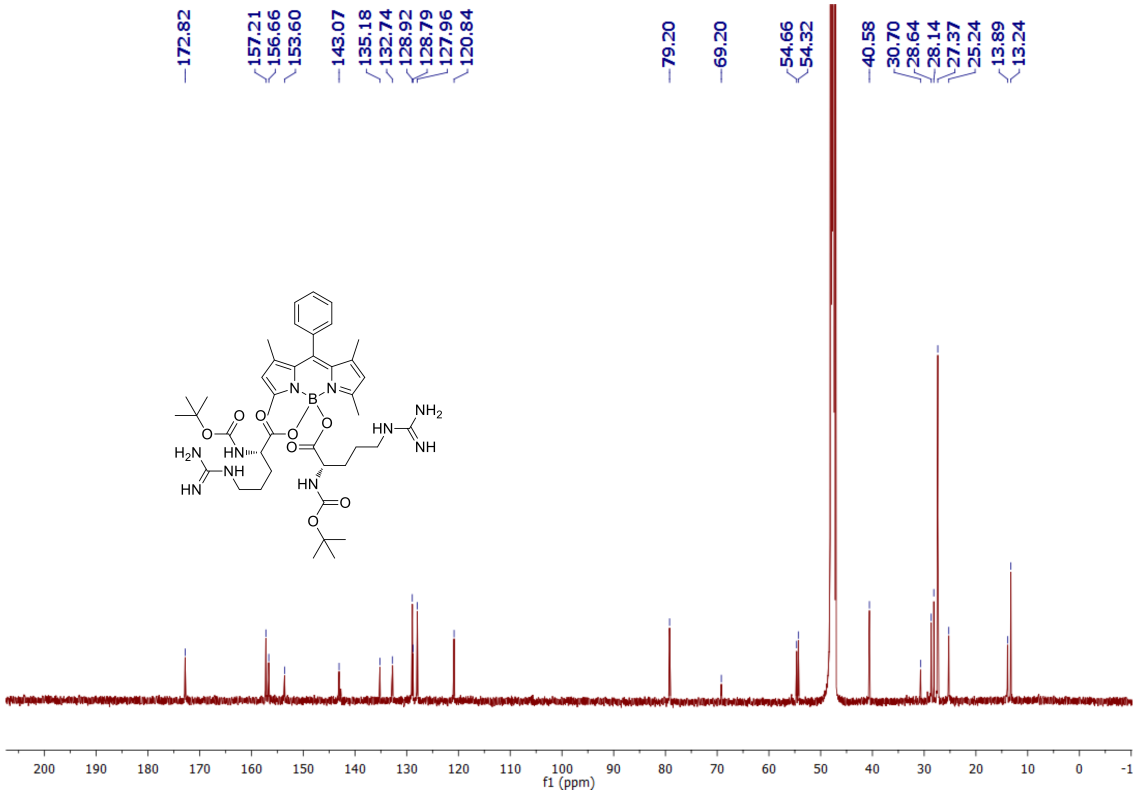

Figure S89. $^{13}C\{^{1}H\}$ NMR (126 MHz, CD_3OD) spectrum of compound **3c**.

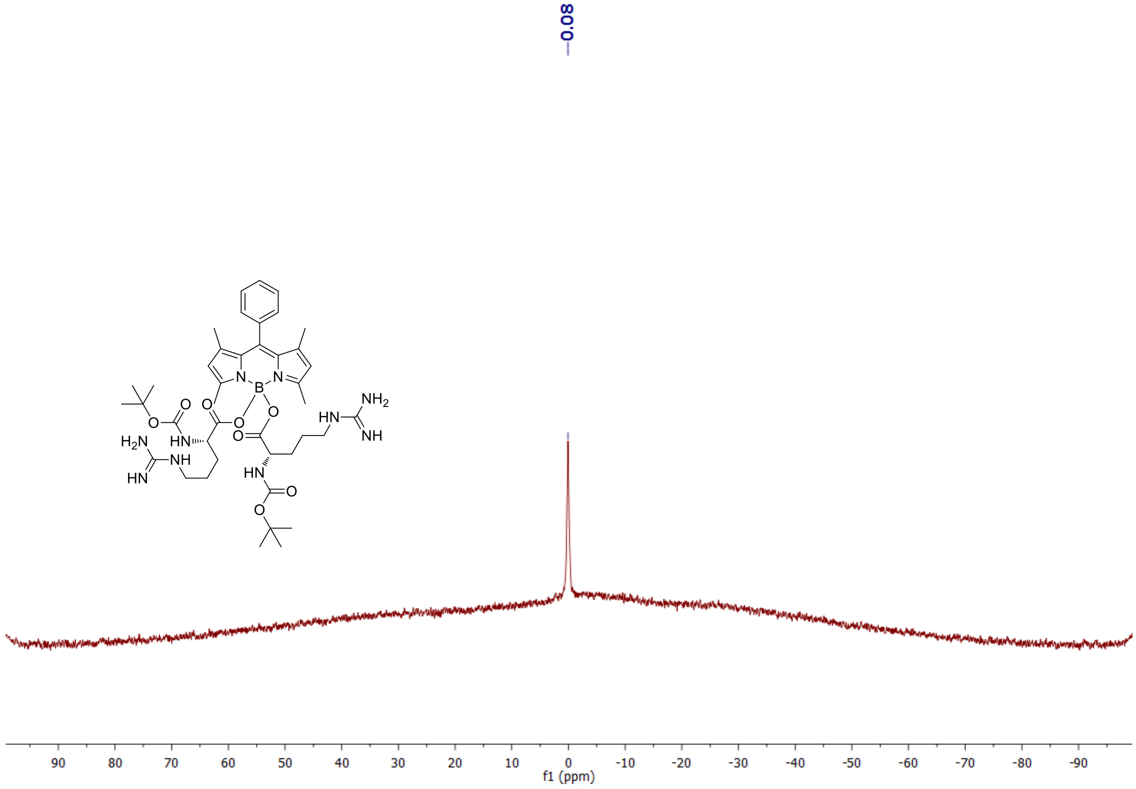

Figure S90. ^{11}B NMR (128 MHz, CD_3OD) spectrum of compound **3c**.

Compound 4b

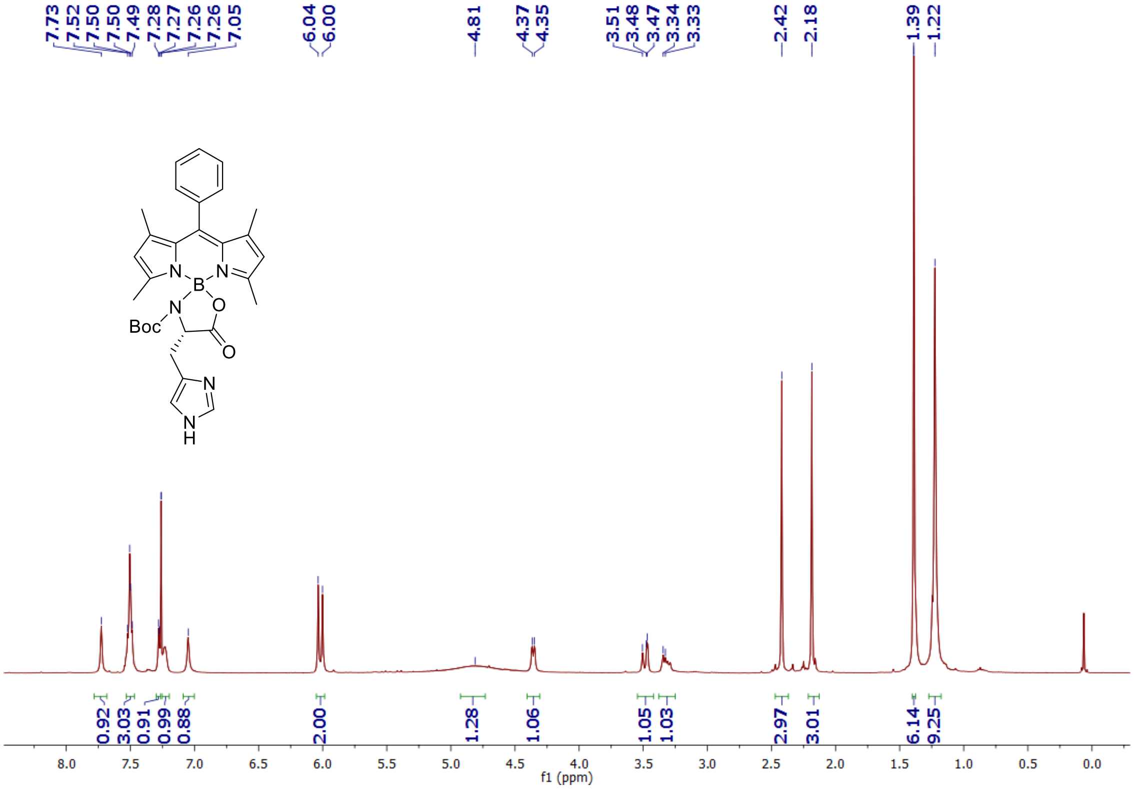

Figure S91. ^{1}H NMR (400 MHz, $CDCl_3$) spectrum of compound **4b**.

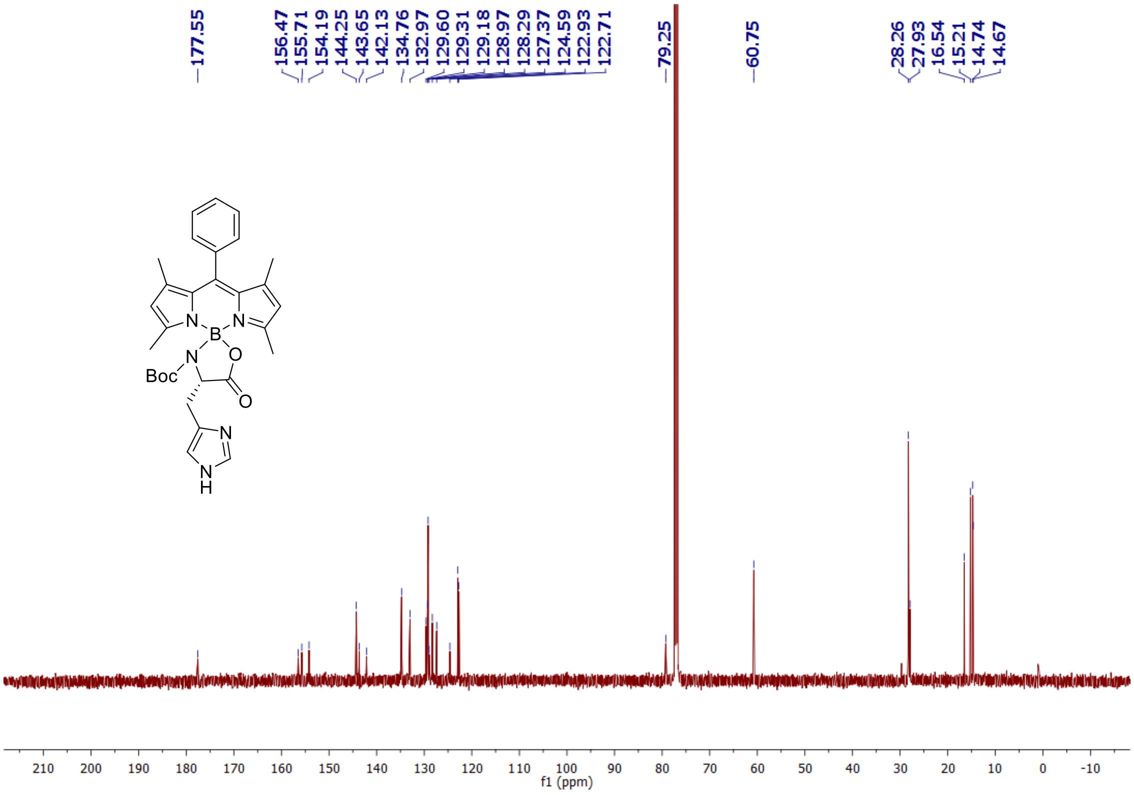

Figure S92. $^{13}C\{^{1}H\}$ NMR (100 MHz, $CDCl_3$) spectrum of compound **4b**.

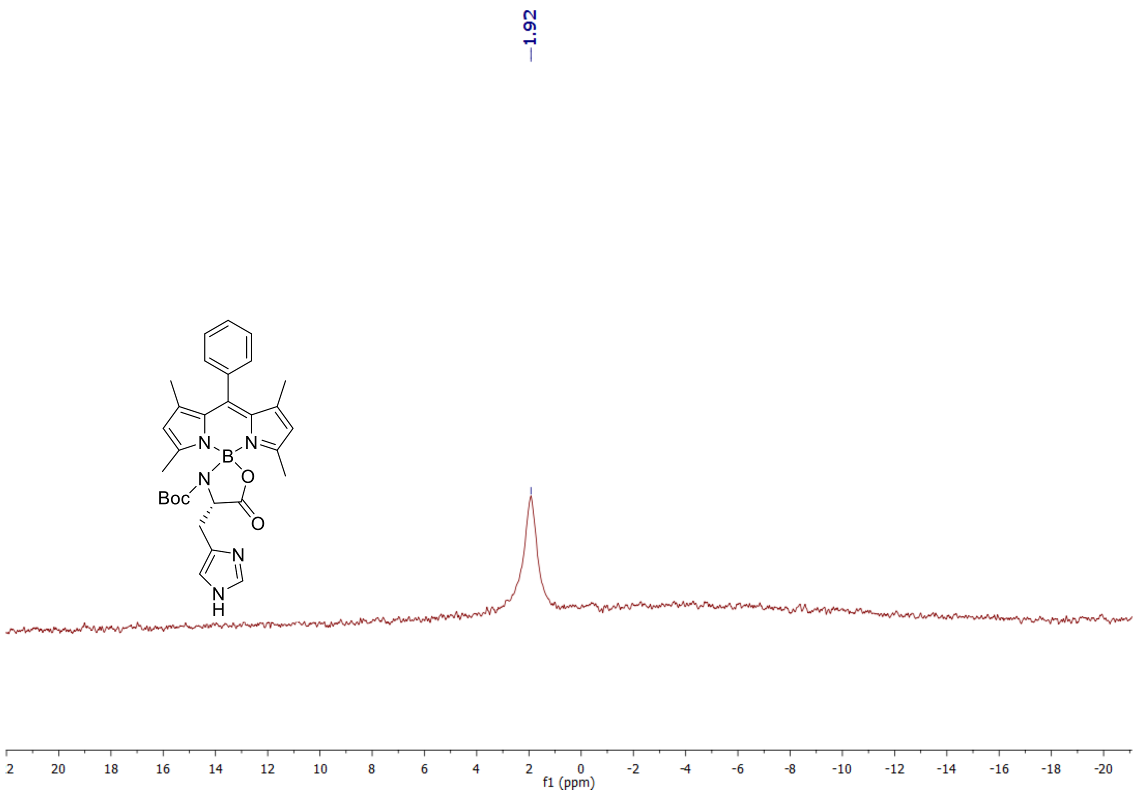

Figure S93. ^{11}B NMR (128 MHz, $CDCl_3$) spectrum of compound **4b**.

Compound 4c

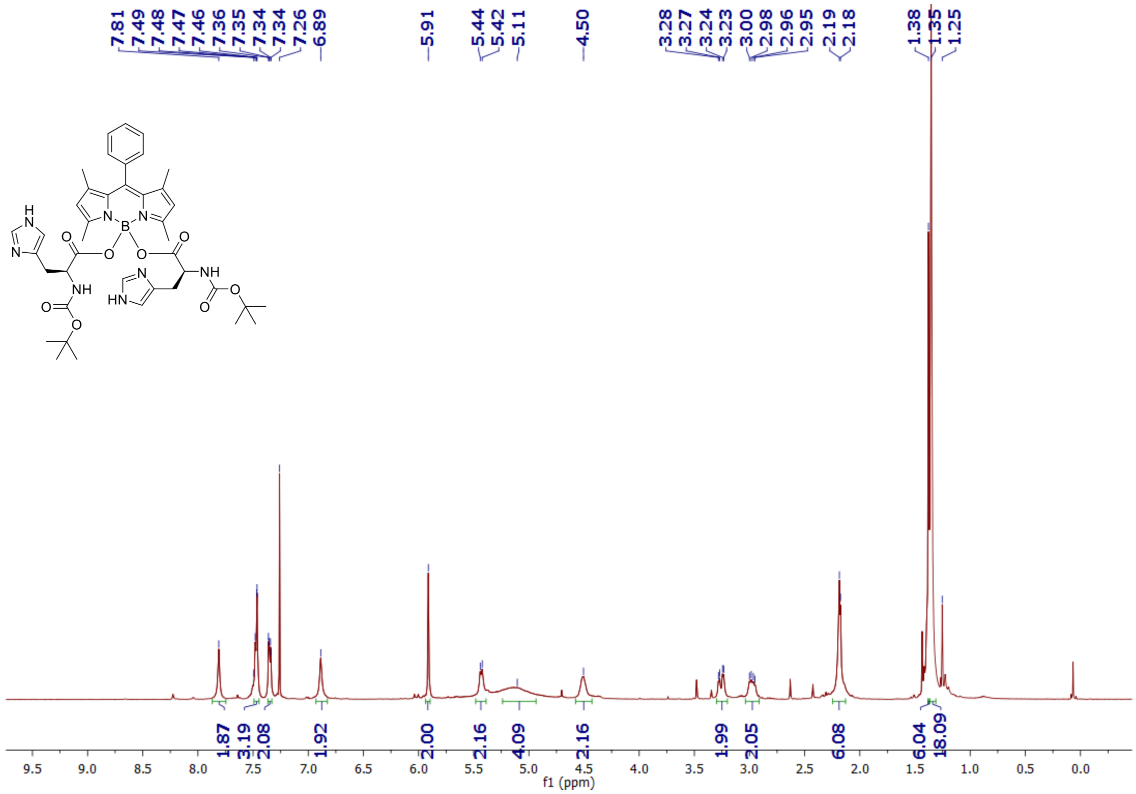

Figure S94. ^{1}H NMR (400 MHz, $CDCl_3$) spectrum of compound **4c**.

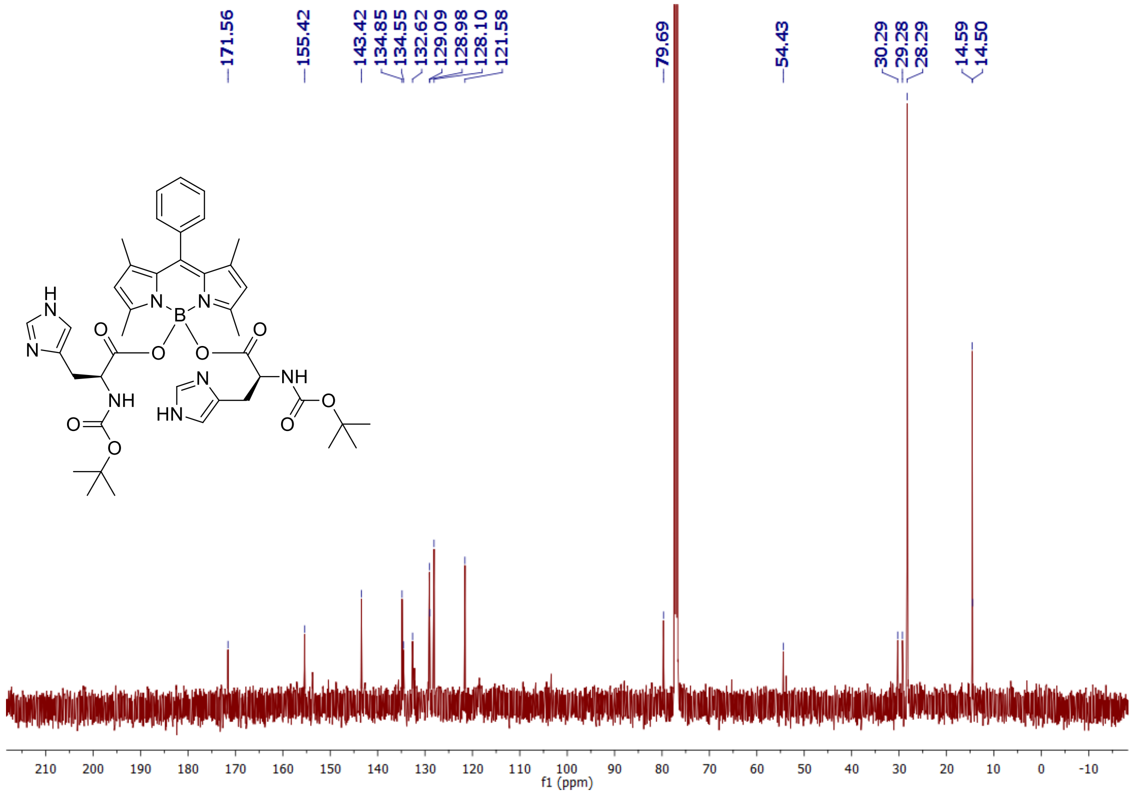

Figure S95. $^{13}C\{^{1}H\}$ NMR (100 MHz, $CDCl_3$) spectrum of compound **4c**.

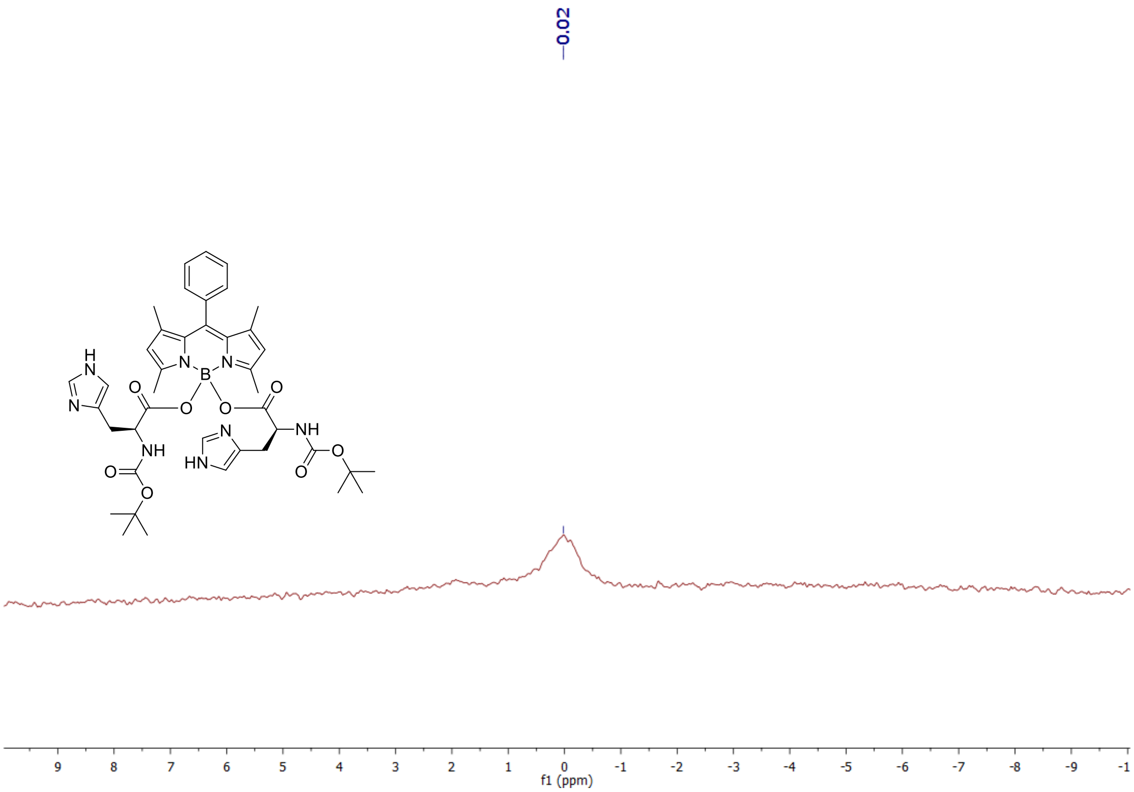

Figure S96. ^{11}B NMR (128 MHz, $CDCl_3$) spectrum of compound **4c**.

Compound 7b

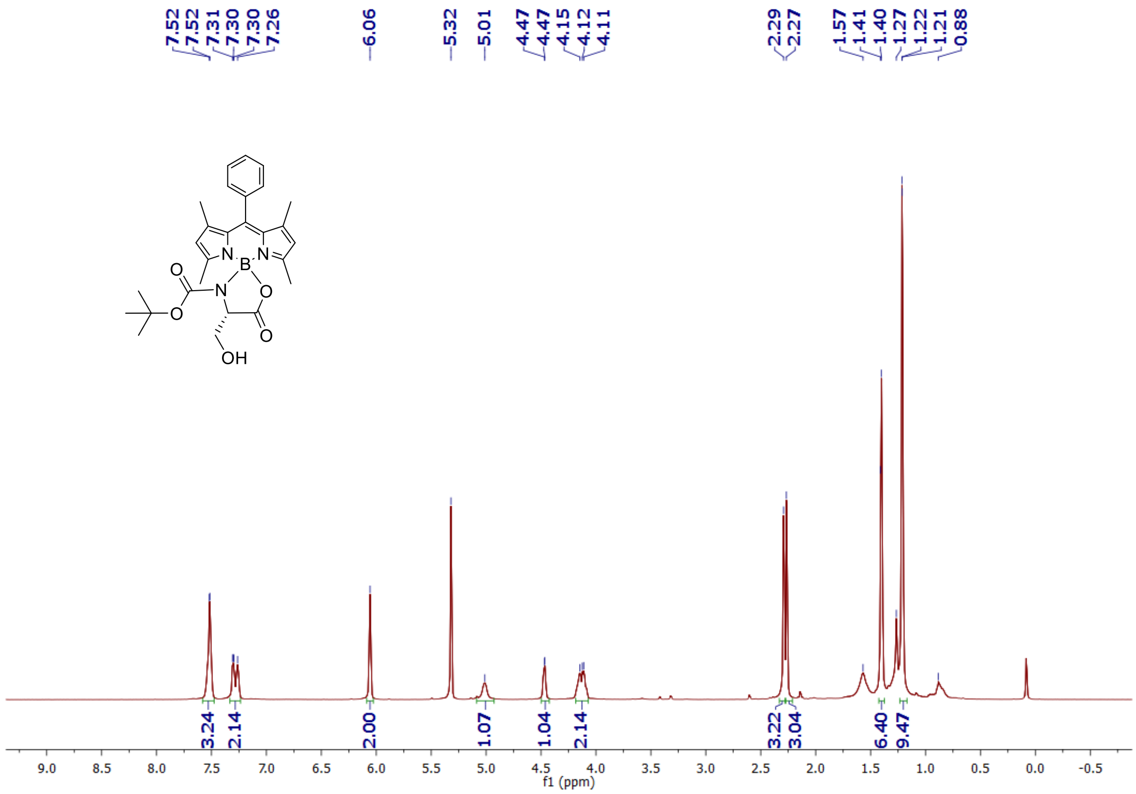

Figure S97. ^{1}H NMR (500 MHz, CD_2Cl_2) spectrum of compound **7b**.

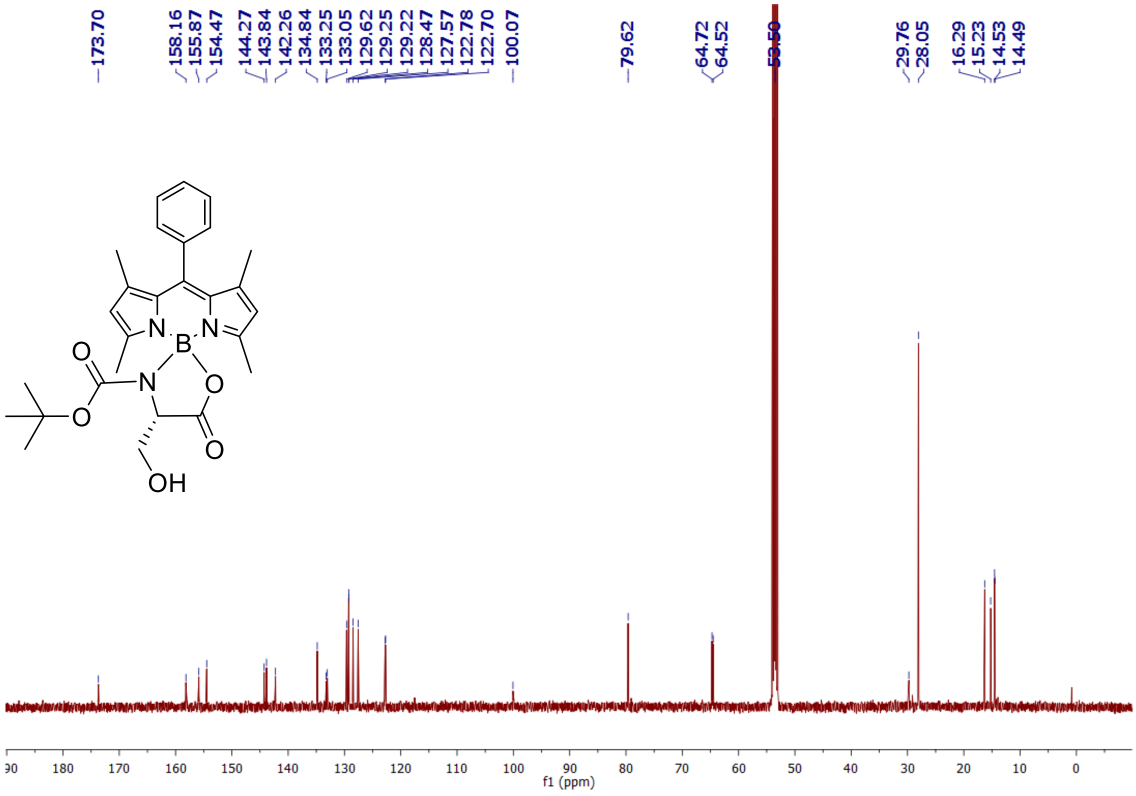

Figure S98. $^{13}C\{^{1}H\}$ NMR (126 MHz, CD_2Cl_2) spectrum of compound **7b**.

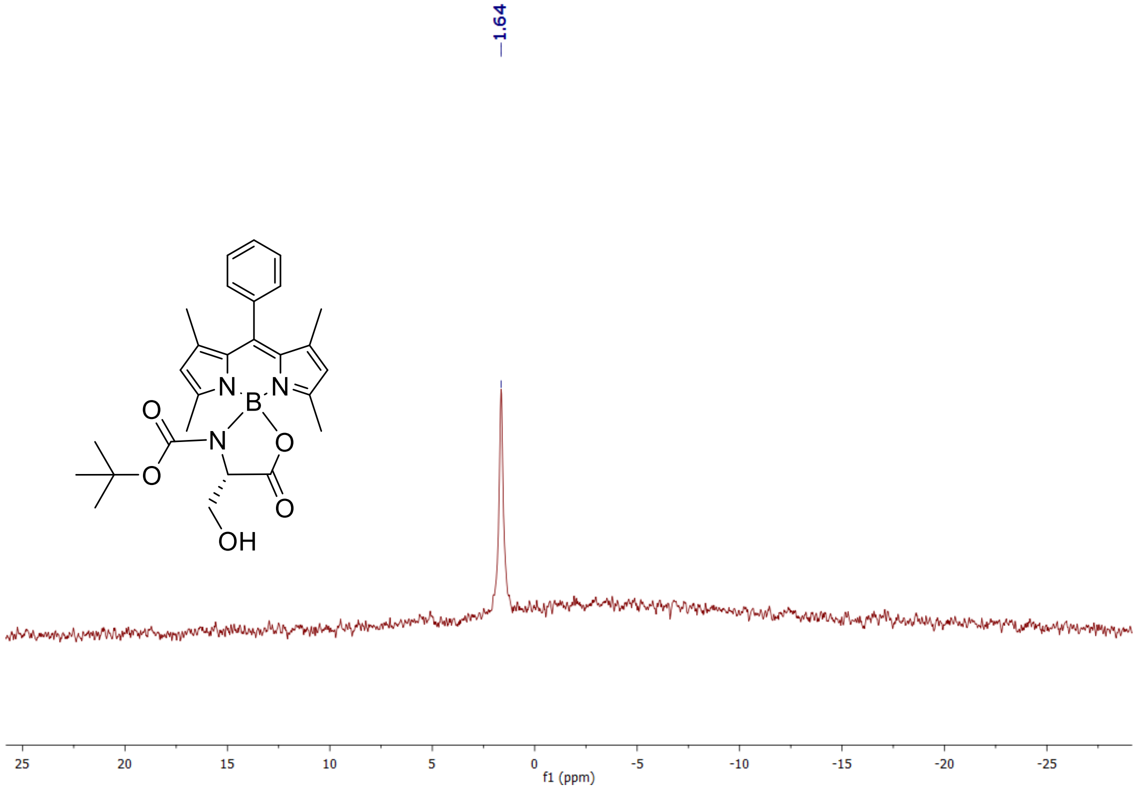

Figure S99. ^{11}B NMR (126 MHz, CD_2Cl_2) spectrum of compound **7b**.

Compound 7c

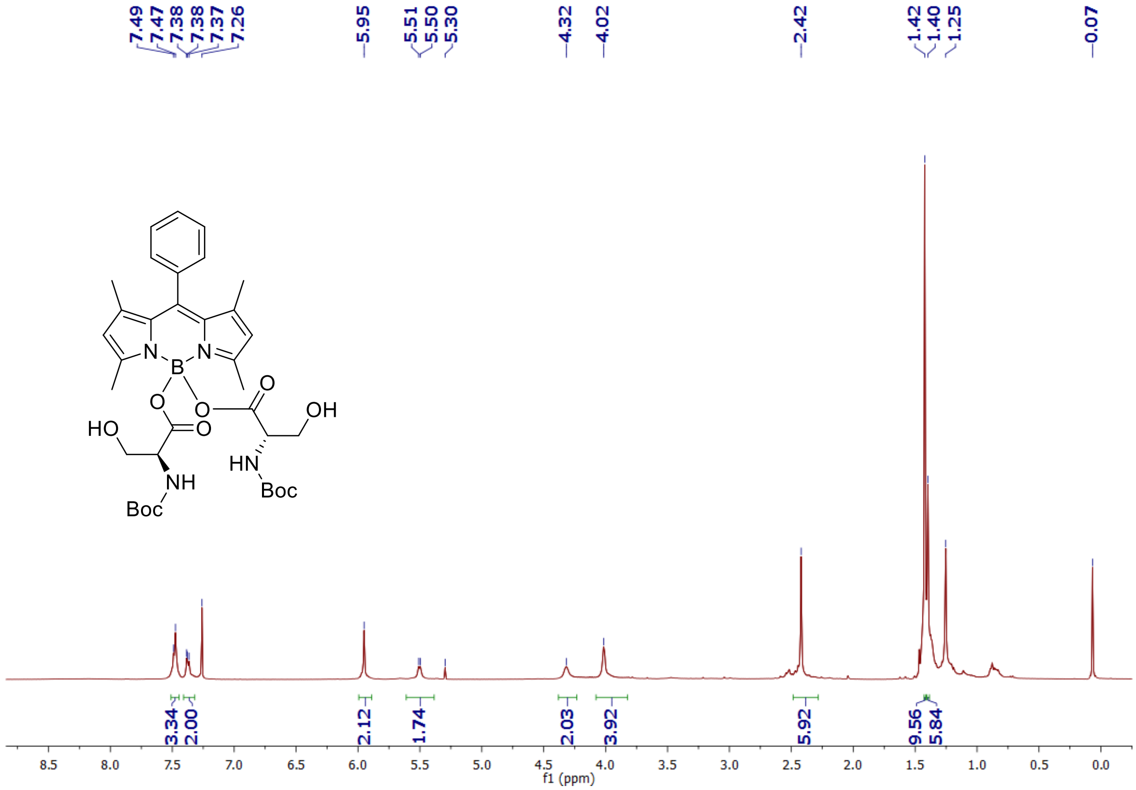

Figure S100. ^{1}H NMR (400 MHz, $CDCl_3$) spectrum of compound **7c**.

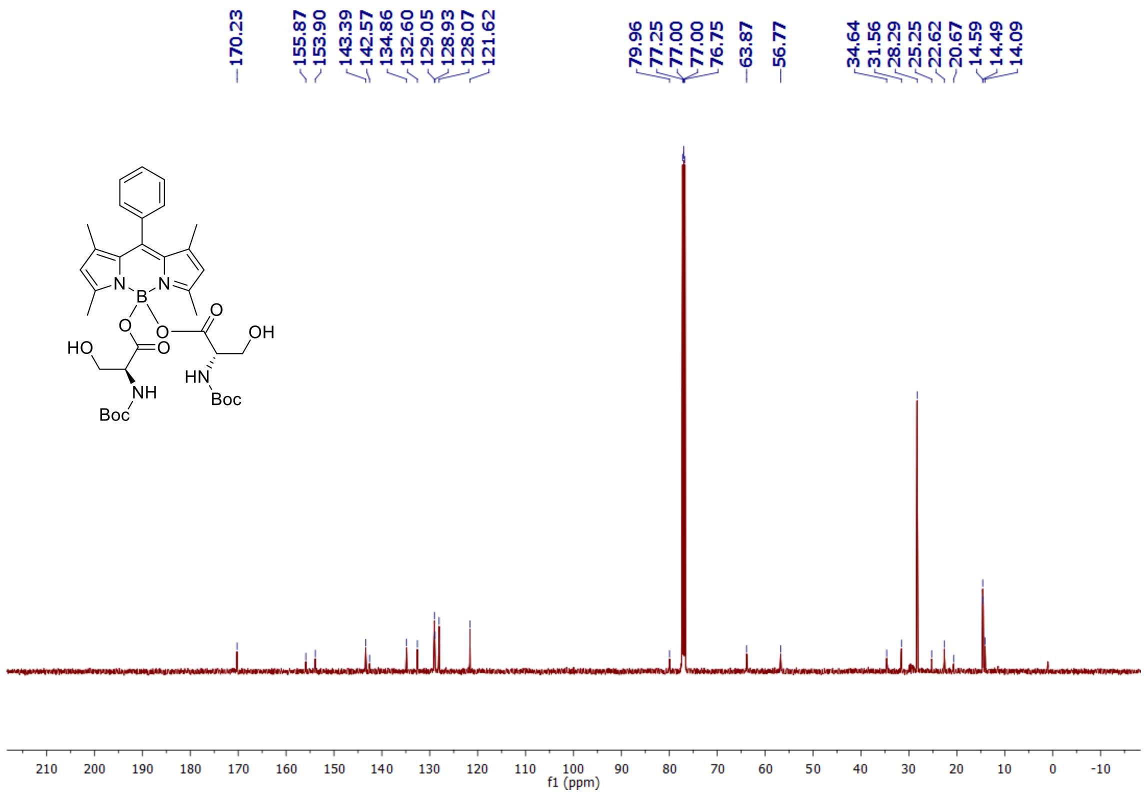

Figure S101. $^{13}C\{^1H\}$ NMR (100 MHz, $CDCl_3$) spectrum of compound **7c**.

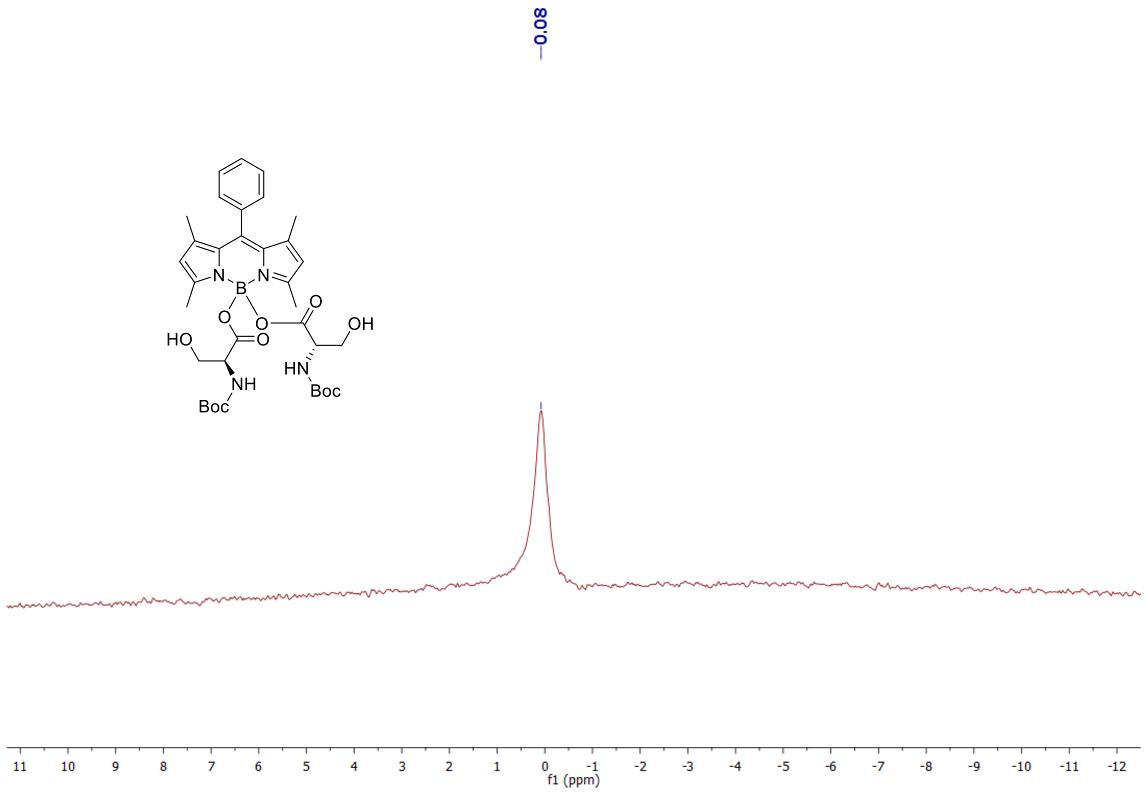

Figure S102. ^{11}B NMR (128 MHz, $CDCl_3$) spectrum of compound **7c**.

Computational Modeling

Table S1. B3LYP/6-31+G(d,p) calculated parameters for BODIPYs **2**-**7**: dipole moments, Gibbs energies relative to the mono-substituted compounds **A**, and optimized bond lengths. All parameters are calculated in dichloromethane.

BODIPY	Dipole (D)	Δ*G* (kcal/mol)	$r_{BN(BODIPY)}$ (Å)	$r_{BN(BODIPY)}$ (Å)	r_{BO} (Å)	$r_{BN(Boc)}$ (Å)
**2a (Asp)**	2.9	0	1.539	1.538	1.505	-
**2b**	15.4	15.9	1.551	1.556	1.504	1.545
**2c**	6.5	16.4	1.538	1.537	1.491	-
**3a (Arg)**	5.0	0	1.537	1.537	1.509	
**3b**	10.3	15.4	1.555	1.563	1.499	1.547
**3c**	7.9	15.6	1.538	1.538	1.498	
**4a (His)**	7.1	0	1.540	1.541	1.499	
**4b**	11.1	18.0	1.554	1.558	1.498	1.541
**4c**	11.7	18.1	1.540	1.540	1.494	
**5A (Tyr)**	4.5	0	1.540	1.540	1.500	
**5B**	11.0	16.1	1.553	1.559	1.500	1.541
**5C**	0.5	21.3	1.539	1.539	1.495	
**6A (Met)**	5.9	0	1.539	1.539	1.504	
**6B**	11.1	13.6	1.553	1.559	1.502	1.541
**6C**	1.1	17.8	1.539	1.538	1.495	
**7a (Ser)**	3.4	0	1.539	1.539	1.500	
**7b**	12.0	17.6	1.552	1.557	1.501	1.541
**7c**	0.9	18.8	1.538	1.537	1.496	

Table S2. TD-DFT M06-2X/6-31+G(d,p) spectroscopic properties of α-amino acid-BODIPYs All parameters are calculated in acetonitrile. The leading transition is HOMO→LUMO for all.

Compound	λ_{abs} (nm)	Oscillator Strength	λ_{em} (nm)
**1**	431	0.63	472
**2b**	436	0.49	473
**2c**	434	0.50	483
**3b**	436	0.49	476
**3c**	433	0.48	470
**4b**	436	0.49	475
**4c**	435	0.50	485
**5B**	436	0.49	488
**5C**	433	0.49	473
**6B**	436	0.49	477
**6C**	434	0.50	467
**7b**	436	0.50	473
**7c**	434	0.51	466

Frontier Orbitals

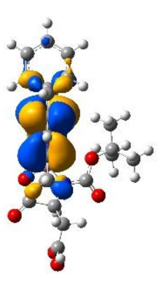 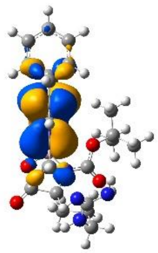 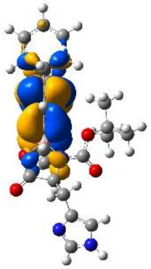 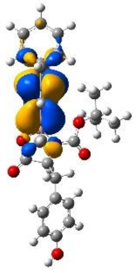 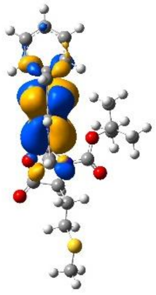 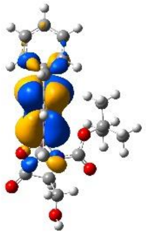

-2.23	-2.20	-2.12	-2.20	-2.21	-2.22

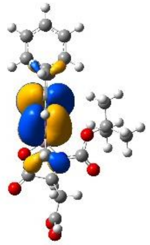 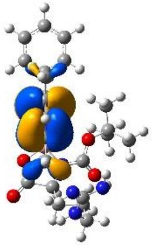 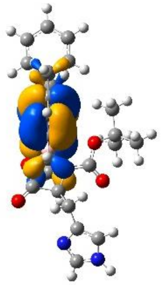 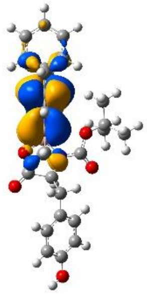 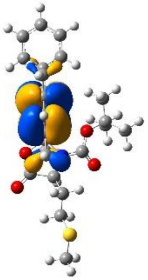 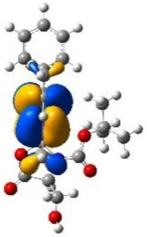

-7.00<br>BODIPY **2b** (Asp)	-6.96<br>BODIPY **3b** (Arg)	-6.91<br>BODIPY **4b** (His)	-6.97<br>BODIPY **5B** (Tyr)	-6.98<br>BODIPY **6B** (Met)	-6.99<br>BODIPY **7b** (Ser)

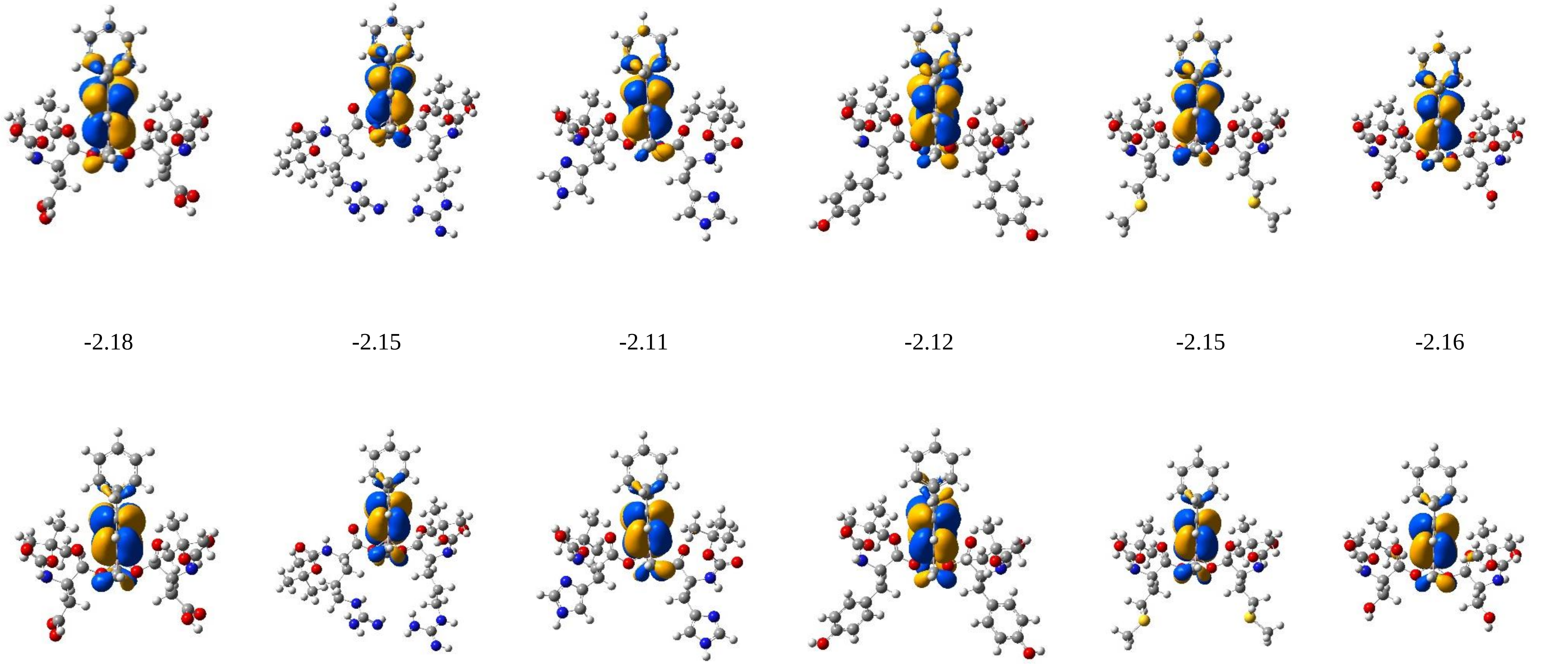

-6.96	-6.93	-6.89	-6.91	-6.93	-6.94
BODIPY **2c** (Asp)	BODIPY **3c** (Arg)	BODIPY **4c** (His)	BODIPY **5C** (Tyr)	BODIPY **6C** (Met)	BODIPY **7c** (Ser)

Figure S103. Frontier orbitals of BODIPYs **2b-7c**. Orbital energies in eV.

Cytotoxicity

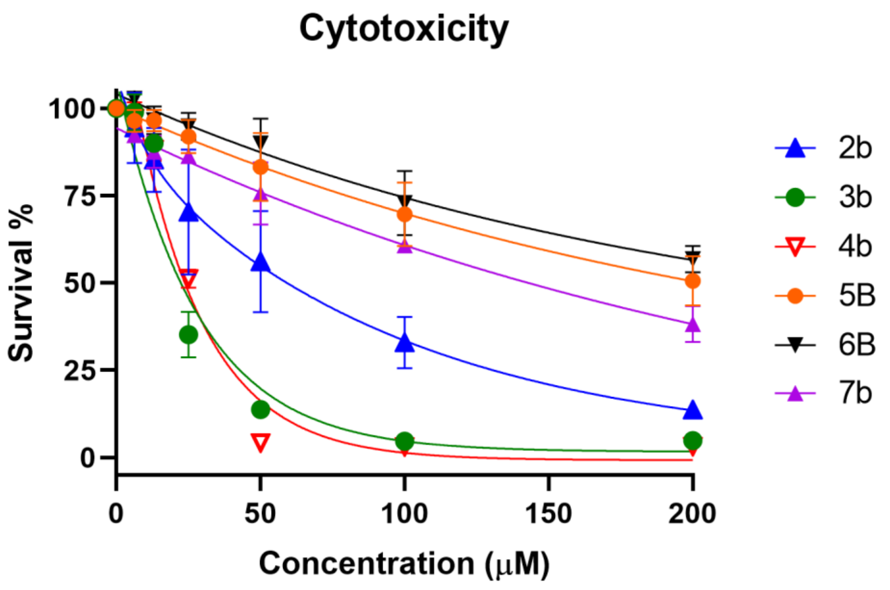

Figure S104. Cytotoxicity of the spiro-compounds **2b-7b** in HEp-2 cells at 6.25 µM, 12.5 µM, 25 µM, 50 µM, 100 µM and 200 µM.

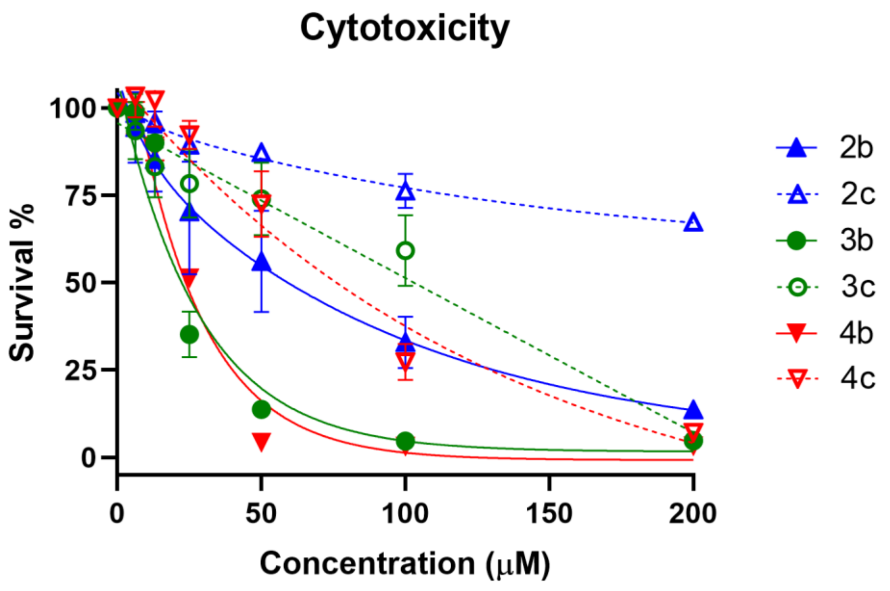

Figure S105. Cytotoxicity of spiro-compounds **2b-4b** (solid) and di-compounds **2c-4c** (dash) in HEp-2 cells at 6.25 µM, 12.5 µM, 25 µM, 50 µM, 100 µM and 200 µM.
